# Lipid droplets promote aberrant liquid-liquid phase separation of alpha-synuclein impairing energy homeostasis

**DOI:** 10.1038/s44319-026-00856-8

**Published:** 2026-07-02

**Authors:** Jose Cevallos, Elena Eubanks, Sunghoo Jung, Yiming Huang, Elyse Guadagno, Nora Jaber, Yuzhou Xia, Jinying Wang, Huan Wang, Neeharika Rao Suvvari, Aryan Doshi, Nitya Ravinutala, Alejandro Mosera, Timothy Hsu, Jiya Mody, Benjamin Sacks, Anvi Narayan, Breanna Smith, Maia Wang, Meghana Gottapu, Heba Alnakhala, Nagendran Ramalingam, Arati Tripathi, Tim Bartels, Ulf Dettmer, Zheng Shi, Wei Dai, Eleanna Kara

**Affiliations:** 1 Department of Neurology, Robert Wood Johnson Medical School, Institute for Neurological Therapeutics at Rutgers, Rutgers Biomedical and Health Sciences, Piscataway, NJ USA; 2 Department of Cell Biology and Neuroscience & Institute for Quantitative Biomedicine, Rutgers University, Piscataway, NJ USA; 3 Department of Chemistry and Chemical Biology, Rutgers University, Piscataway, NJ USA; 4 Ann Romney Center for Neurologic Diseases, Brigham and Women’s Hospital, Boston, MA USA; 5 Harvard Medical School, Boston, MA USA; 6 UK Dementia Research Institute, University College London, London, UK; 7 Present Address: Department of Chemistry, Stanford University, Stanford, CA USA; 8 Present Address: Wu Tsai Neurosciences Institute and ChEM-H Institute, Stanford University, Stanford, CA USA; 9 Present Address: Bowles Center for Alcohol Studies, School of Medicine, University of North Carolina, Chapel Hill, NC USA; 10 Present Address: Department of Molecular Biophysics and Biochemistry, Yale University, New Haven, CT USA; 11 Present Address: Department of Radiology, Athinoula A. Martinos Center for Biomedical Imaging, Massachusetts General Hospital, Charlestown, MA USA; 12 Present Address: Byrd Alzheimer’s Center & Research Institute, University of South Florida, Tampa, FL USA; 13 Present Address: Department of Molecular Medicine, USF Morsani College of Medicine, Tampa, FL USA

**Keywords:** Metabolism, Neuroscience

## Abstract

Alpha-synuclein (αSyn) inclusions are a defining neuropathological feature of Parkinson’s disease, but the cellular events that initiate their formation and promote neurotoxicity remain incompletely understood. Aberrant liquid–liquid phase separation has emerged as a potential early step in αSyn dysregulation, yet the physiological triggers and functional consequences of this process are unclear. Here, we show that lipid droplets promote the spontaneous phase separation of wild-type and E46K mutant αSyn into condensates. These condensates sequester lipid droplets and impair their turnover, indicating disruption of cellular lipid homeostasis. Mitochondria in close proximity to αSyn condensates exhibit reduced membrane potential and increased mitophagy. Correlative light and electron microscopy further reveals αSyn oligomers associated with mitochondrial membranes displaying structural abnormalities. Together, these findings identify lipid droplets as drivers of aberrant αSyn phase separation and suggest that lipid droplet-rich condensates contribute to mitochondrial dysfunction and impaired energy homeostasis. Given the enrichment of lipid droplets within neuromelanin-containing dopaminergic neurons of the substantia nigra, this mechanism may be relevant to the selective neuronal vulnerability observed in Parkinson’s disease.

## Synopsis

Lipid droplets promote aberrant phase separation of alpha-synuclein into biomolecular condensates that disrupt lipid metabolism and potentially mitochondrial homeostasis, suggesting links between lipid droplets, alpha-synuclein dysregulation, and cellular dysfunction in Parkinson’s disease.

- Lipid droplets induce the formation of alpha-synuclein condensates by wild-type and E46K mutant alpha-synuclein, supporting a role for lipid metabolism in aberrant phase separation.
- Alpha-synuclein condensates sequester lipid droplets and impair their turnover, suggesting disruption of cellular energy homeostasis.
- Mitochondria adjacent to alpha-synuclein condensates are depolarized and undergo increased mitophagy, while correlative light and electron microscopy reveals alpha-synuclein oligomers associated with structurally abnormal mitochondrial membranes.

## Abstract (web summary)

Lipid droplets promote aberrant phase separation of alpha-synuclein into biomolecular condensates that disrupt lipid metabolism and potentially mitochondrial homeostasis, suggesting links between lipid droplets, alpha-synuclein dysregulation, and cellular dysfunction in Parkinson’s disease.

## Graphical abstract

**Figure:**
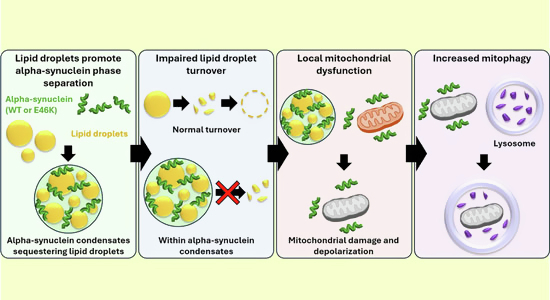

**Figure:**
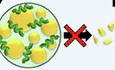

## Introduction

It is thought that aberrant liquid–liquid phase separation (LLPS) is the first step in the process leading to the formation of proteinaceous inclusions that are involved in the pathogenesis of neurodegenerative diseases. The amyloidogenic proteins involved in these diseases either independently phase separate or partition (get recruited) into a pre-existing condensate that is formed by other proteins (Visser et al, 2024). These condensates then undergo a liquid-to-solid transition because of locally increased protein concentration leading to aggregation (Dai et al, 2024; Putnam et al, 2023). This process eventually leads to the formation of the characteristic proteinaceous inclusions seen in neurodegenerative diseases. This phenomenon has been well studied for tau (Boyko et al, 2019; Hochmair et al, 2022; Wegmann et al, 2018) and TAR DNA-binding protein 43 (TDP-43) (Schmidt and Rohatgi, 2016; Yan et al, 2025) and has also been observed for amyloid-beta (Morris et al, 2024).

There is recent evidence suggesting that aberrant LLPS of alpha-synuclein (αSyn) is involved in its dysregulation in Parkinson’s disease (PD) and Dementia with Lewy bodies (DLB), eventually leading to the formation of Lewy bodies (Agarwal et al, 2024; Dada et al, 2023; Eubanks et al, 2025; Hardenberg et al, 2021; Mukherjee et al, 2023; Ray et al, 2020; Wang et al, 2024). αSyn is an intrinsically disordered protein that contains two low complexity domains in its N-terminus and non-amyloid beta component (NAC) region and an intrinsically disordered C-terminus that are predicted to increase its proclivity to phase separate (Mukherjee et al, 2023). In vitro experiments have shown that αSyn phase separates when there is increased molecular crowding (Ray et al, 2020), though the concentrations required are non-physiologically high. Post-translational modifications, mutations, pH changes, metal ions and binding partners such as the R-SNARE protein VAMP2 (synaptobrevin-2) and synapsin facilitate its phase separation (Agarwal et al, 2024; Dada et al, 2023; Hoffmann et al, 2021; Huang et al, 2022; Sawner et al, 2021; Wang et al, 2024; Xu et al, 2022). Aberrant LLPS of αSyn leads to a metastable porous hydrogel stage that eventually transitions into solid β-sheet-rich fibrillar aggregates (Hardenberg et al, 2021; Piroska et al, 2023; Ray et al, 2020). It has also been found that intermixed steps of LLPS and aggregation lead to the formation of αSyn inclusions (Eubanks et al, 2025).

Lipids are also involved in this process, in keeping with the nature of αSyn as a membrane-binding protein and with the fact that at least a proportion of Lewy bodies is rich in vesicles, membranes and lipids (Moors et al, 2021; Shahmoradian et al, 2019). Lipids influence the kinetics underlying the liquid-to-solid transition of αSyn: liposomes, short saturated lipids and water soluble lipids arrest the maturation of αSyn condensates at the porous hydrogel stage, whereas long-chain lipids, cholesterol and fluid anionic membranes have the opposite effect (Galvagnion et al, 2016; Hardenberg et al, 2021).

Lipid droplets are connected to the dysregulation of αSyn. αSyn overexpression is linked to lipid droplet accumulation, and αSyn binding to lipid droplets leads to the formation of proteolysis-resistant αSyn aggregates (Girard et al, 2021; Smith et al, 2023). αSyn can also bind to the surface of lipid droplets and reduce the turnover of triglycerides (Cole et al, 2002). This connection could be particularly relevant to the pathogenesis of PD because midbrain dopaminergic neurons in the substantia nigra, which is one of the most vulnerable brain regions in PD, contain neuromelanin that is rich in lipid droplets (Filimontseva et al, 2025). Indeed, it has been shown that αSyn aggregates onto lipids present within neuromelanin, and that changes in the lipids contained within neuromelanin precede the formation of Lewy bodies (Halliday et al, 2005).

Given these observations that aberrant LLPS, lipid dysregulation, lipid droplets and neuromelanin are all involved in αSyn dysregulation in PD, we propose an intriguing hypothesis: aberrant LLPS of αSyn upon binding to lipid droplets leads to the formation of hydrogel-like (semi-liquid/semi-solid) biomolecular condensates that are neurotoxic and cause neurodegeneration. In this manuscript, we tested this hypothesis, and our findings suggest a conceptual advance in our understanding of the pathogenesis of PD. We showed that αSyn condensates, particularly those formed by E46K mutant αSyn, a Mendelian autosomal dominant mutation causing familial PD, are cytotoxic and do not simply represent a metastable phase in the liquid-to-solid transition process. This toxicity occurs because they sequester lipid droplets and impede their turnover and consequent energy provision in the cell, as well as directly impair the function of neighbouring mitochondria.

## Results

### Oleic acid treatment and lipid droplets induce the formation of αSyn inclusions

Recapitulating αSyn inclusions in cultured cells is a conundrum as none of the currently available model systems form Lewy body-like inclusions in a physiologically relevant manner. Seeding with recombinant αSyn pre-formed fibrils (PFFs) or human postmortem brain-derived fibrils leads to the formation of inclusions with no or limited ultrastructural similarities to Lewy bodies (Mahul-Mellier et al, 2020); in addition, the structure of recombinant PFFs does not accurately recapitulate that of αSyn fibrils from human brain (Todd et al, 2024). αSyn that carries three glutamic acid (E) to lysine (K) mutations in the KTKEGV repeat motifs in αSyn (E35K, E46K, E61K, “3K” αSyn) forms inclusions that ultrastructurally recapitulate a proportion of Lewy bodies (Moors et al, 2021; Shahmoradian et al, 2019), in that they are rich in vesicles, lipids and membranes that are surrounded by amorphously aggregated αSyn (Dettmer et al, 2015a; Dettmer et al, 2015b; Dettmer et al, 2017; Ericsson et al, 2021; Eubanks et al, 2025). However, the reliance on two artificial mutations (E35K, E61K) to “amplify” the effect of the PD Mendelian E46K mutation raises concerns about the true physiological relevance of this model.

We sought to develop a model system for αSyn inclusions that is more physiologically relevant and builds upon αSyn’s well characterized ability to aberrantly phase separate (Agarwal et al, 2024; Dada et al, 2023; Hardenberg et al, 2021; Hoffmann et al, 2021; Huang et al, 2022; Piroska et al, 2023; Ray et al, 2020; Sawner et al, 2021; Wang et al, 2024; Xu et al, 2022). Previous evidence suggested a potential relevance of lipid droplets to this process: It was recently reported that binding to lipid droplets promotes the phase separation of intrinsically disordered proteins (Kamatar et al, 2024). In addition, in our recent study we showed that 3K αSyn spontaneously forms lipid droplet-rich inclusions, and that this ability is enhanced by the presence of increased numbers of lipid droplets (Eubanks et al, 2025). Finally, lipid droplets are enriched within the neuromelanin-rich dopaminergic neurons in the human substantia nigra, the most vulnerable brain region in PD (Filimontseva et al, 2025).

**Figure 1:**
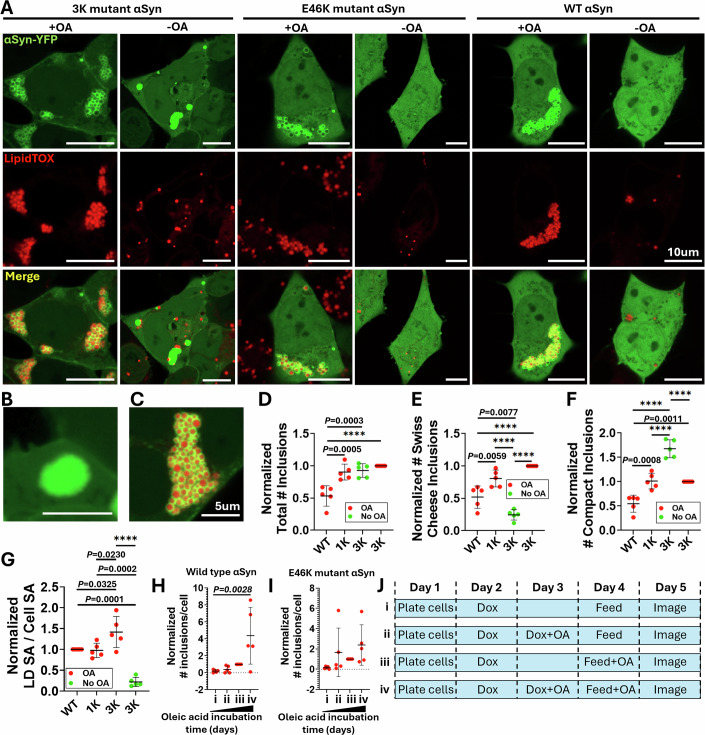
Lipid droplets induce the formation of αSyn inclusions. (**A**) Confocal images of M17D cells expressing wild-type (WT) or E46K mutant (1K) αSyn-Venus YFP after treatment with 600 μM of oleic acid (OA) for 16 h show the spontaneous formation of inclusions. Cells without oleic acid treatment did not form αSyn inclusions. Scale bar = 10 μm. (**B**) Magnified view of a “Compact” 3K αSyn inclusion, without lipid droplet entrapment. Scale bar = 5 μm. (**C**) Magnified view of a “Swiss cheese” 3K αSyn inclusion (in green), with lipid droplet entrapment as indicated through LipidTOX staining (in red). Scale bar = 5 μm. (**D**–**F**) The number of αSyn inclusions per cell was quantified in αSyn-YFP expression-matched cells after induction with doxycycline for 48 h followed by treatment with 600 μM of OA for 24 h. The total number of inclusions was quantified (**D**). Distinction was made between lipid droplet-rich “Swiss cheese” (**E**) and lipid droplet-poor “Compact” (**F**) inclusions. ×20 images were acquired for this experiment, and they correspond to the first imaging day of the oleic acid pulse-chase experiment described in Fig. 4. One-way ANOVA with Tukey’s multiple comparison test. (**G**) In the same αSyn-YFP expression-matched cells included in the analyses shown in (**D**–**F**), we quantified the surface area of lipid droplets which we divided with the surface area of the cytoplasm. One-way ANOVA with Tukey’s multiple comparison test. (**H**–**J**) Directional increase in the number of αSyn condensates depending on the amount of time oleic acid was in the culture media. (**J**) describes each of the four conditions shown in (**H**, **I**). From left to right: (i) No oleic acid; (ii) oleic acid was added 1 day after doxycycline (Dox) induction, removed the following day and the cells were imaged the day after; (iii) oleic acid was added 2 days after doxycycline induction and the cells were imaged the day after; (iv) oleic acid was added 1 day after doxycycline induction, re-added the following day, and the cells were imaged the day after. For this experiment, 3 × 3 ×20 tiling images were acquired. One-way ANOVA with test for linear trend. The results were significant for wild-type (WT) αSyn (**H**) and there was a trend in the same direction for 1K αSyn (**I**). All experiments in this figure and everywhere else in the manuscript were undertaken after 48 h of doxycycline induction unless otherwise stated. In addition, wherever cells were treated with OA, the concentration was 600 μM for 24 h, unless otherwise stated. All experiments were repeated independently five times, and the normalized values are shown in each plot. Data are represented as mean +/− SD. *P* values: ****≤0.0001.

We treated M17D neuroblastoma cells stably expressing doxycycline-inducible αSyn-YFP constructs (wild-type (WT), E46K (referred to as 1K, indicating a single lysine substitution), and the engineered 3K mutant (E35K/E46K/E61K)) with oleic acid to promote lipid droplet formation, a well-established approach (Papadopoulos et al, 2015). The M17D cell line was selected due to its neuronal origin and prior use in studies of αSyn dysregulation (Dettmer et al, 2017; Ericsson et al, 2021; Imberdis et al, 2019). In the absence of oleic acid, WT and 1K αSyn did not form inclusions, whereas 3K αSyn readily formed inclusions. Upon oleic acid treatment, both WT and 1K αSyn formed lipid droplet-rich inclusions (Fig. 1A). Close up views of a “Compact” inclusion that does not contain lipid droplets and a “Swiss Cheese” inclusion rich in lipid droplets are shown in Fig. 1B and  1C, respectively. The E46K (1K) mutant was included as a clinically relevant, single-point mutation that increases αSyn’s interaction with lipid membranes, providing an intermediate condition between WT and 3K variants.

We next quantified inclusion formation across conditions. To ensure that the observed differences are due to the conditions being tested and not to unequal αSyn abundance between cells, we controlled for the differences in αSyn expression: we analyzed cells with matched protein levels, determined by the single-cell mean fluorescence intensity (MFI) of αSyn-YFP. Under these controlled conditions, 1K αSyn in the presence of oleic acid and 3K αSyn (with or without oleic acid) formed comparable numbers of inclusions per cell. In contrast, WT αSyn formed inclusions at approximately half this frequency (Fig. 1D).

We further distinguished between lipid droplet-rich (“Swiss cheese”) (Fig. 1E) and lipid droplet-poor (“compact”) inclusions (Fig. 1F). In 3K-expressing cells, oleic acid treatment increased the frequency of Swiss cheese inclusions while reducing compact inclusions. WT αSyn formed significantly fewer inclusions of both types compared to 1K and 3K variants. Notably, under oleic acid treatment, 1K and 3K αSyn formed Swiss cheese inclusions at similar frequencies. However, oleic acid-treated 1K αSyn produced more Swiss cheese inclusions and fewer compact inclusions compared to untreated 3K αSyn (Fig. 1E,F). Of note, oleic acid treatment induced lipid droplet formation to a similar extent across WT, 1K, and 3K cell lines, and was marginally more efficient in 3K cells (Fig. 1G). These findings suggest that the efficiency of inclusion formation is modulated by the increased positive charge of αSyn introduced by additional lysine residues.

The number of inclusions formed by WT (Fig. 1H) or E46K mutant αSyn (Fig. 1I) was proportional to the amount of time oleic acid was present in the culture media; the effect also diminished when oleic acid-containing media was replaced for 24 h. Experimental design is shown in Fig. 1J.

### WT, 1K, and 3K αSyn inclusions exhibit properties consistent with biomolecular condensates

**Figure 2:**
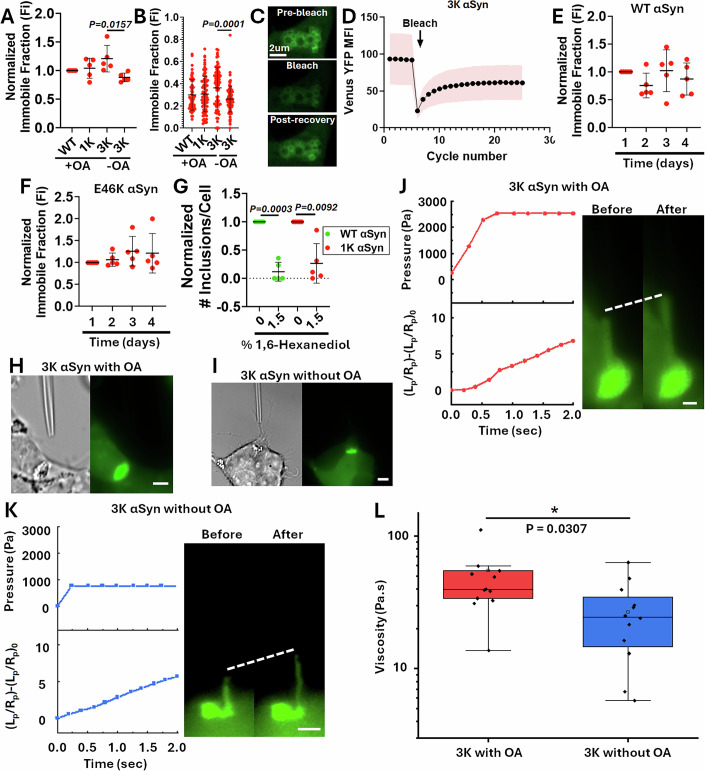
WT, 1K, and 3K αSyn inclusions exhibit properties consistent with biomolecular condensates. (**A**, **B**) FRAP on WT, 1K and 3K αSyn inclusions with and without oleic acid (OA) treatment. (**A**) shows the normalized data, whereas (**B**) is the unnormalized version of the same data (N: 3K no OA = 87; 3K OA = 75; 1K OA = 81; WT OA = 63). The no OA treatment condition applies only to 3K αSyn because it is the only one that spontaneously forms inclusions without lipid droplets. All conditions showed similar immobile fractions (Fi) indicating that they have similar liquid and solid proportions (liquid > solid). One-way ANOVA with Tukey’s post hoc correction. (**C**) Representative FRAP images of a 1K αSyn inclusion. (**D**) Representative FRAP recovery plot for 3K αSyn inclusions without OA treatment. The standard deviation (SD) is shown in pink. (**E**,** F**) Timecourse FRAP experiments for WT and 1K αSyn inclusions formed after induction with doxycycline for 48 h followed by treatment with 600 μM of OA for 24 h. Day 1 is the first day after the 24 h incubation of OA was completed. FRAP was measured once every 24 h over 4 days. One-way ANOVA with test for linear trend. (**G**) Treatment with 1.5% of 1,6-Hexanediol results in the significant reduction in the number of 1K and WT αSyn inclusions. One sample *t* tests. All experiments were repeated independently five times, and the normalized values are shown in each plot, apart from (**B**) that contains the unnormalized pooled values from all independent experiments. (**H**, **I**) Representative cell images of 3K with/without OA acquired during the MAPAC experiments. Cells were measured after 48 h of doxycycline induction, followed by treatment with 600 μM of OA for 24 h. Scale bars = 5 µm. (**J**, **K**) Representative MAPAC measurement of an αSyn condensate in 3K cells with/without OA. Left top: Applied aspiration pressure as a function of time. Left bottom: Corresponding normalized deformation length ((L_p_/R_p_)-(L_p_/R_p_)_0_) over time. Right: Fluorescence images on the right show the condensates before and after aspiration. Scale bars = 2μm. The dashed line indicates the position of aspirated condensate inside the micropipette after pressure application. It is used to visually mark the extent of condensate deformation/climbing into the pipette under the applied aspiration pressure. (**L**) Viscosity of αSyn condensate (3K with OA, *n* = 13; and 3K without OA, *n* = 12. Each condensate has 2–3 tests to get the average viscosity measured by MAPAC. The center line indicates the median; the box bounds represent the 25th and 75th percentiles; the whiskers extend to the most extreme data points within 1.5× the interquartile range; and each point represents an independent measurement. *P* values determined using two sample *t* test. Data are represented as mean +/− SD.

To determine the material properties of αSyn inclusions, we performed fluorescence recovery after photobleaching (FRAP) experiments. WT and 1K αSyn inclusions formed in the presence of oleic acid exhibited similar immobile fractions (Fi ~0.35) to 3K αSyn inclusions under the same conditions (Fig. 2A,B), indicating a predominantly liquid-like state. Interestingly, 3K αSyn inclusions that formed spontaneously, in the absence of oleic acid, were significantly more dynamic than those formed following oleic acid treatment, suggesting that increased lipid droplet association reduces internal molecular mobility (Fig. 2A,B). Representative FRAP images are shown in Fig. 2C and FRAP recovery plot in Fig. 2D. In order to characterize the maturation of αSyn condensates over time, we performed a 4-day timecourse experiment. FRAP measurements conducted every 24 h showed that the immobile fractions of WT (Fig. 2E) and 1K (Fig. 2F) αSyn condensates remain relatively stable, though the 1K variant shows a trend toward increased immobility over time. In our previous work, a similarly designed experiment for 3K αSyn showed a significant increase in Fi over 4 days (Eubanks et al, 2025). To further probe the physical nature of these assemblies, we treated cells with 1.5% 1,6-hexanediol, a disruptor of weak hydrophobic interactions characteristic of biomolecular condensates. This treatment led to a significant reduction in the number of WT and 1K αSyn inclusions (Fig. 2G), consistent with our previous observations for 3K αSyn inclusions (Eubanks et al, 2025). Together, these results indicate that lipid droplet-associated WT and 1K αSyn inclusions share key material properties with biomolecular condensates.

To directly quantify the material properties of intracellular αSyn condensates, we performed micropipette aspiration and whole-cell patch clamp (MAPAC) measurements (Wang et al, 2025) on 3K αSyn condensates formed in M17D cells in the presence or absence of oleic acid. In this assay, a single condensate was gently drawn into the micropipette by stepwise aspiration pressure two to three times, and its deformation was quantified as the change in normalized aspiration length over time (Fig. 2H,I). Representative measurements showed progressive condensate entry into the pipette under applied pressure in both conditions, consistent with an apparent viscous response rather than purely elastic deformation (Fig. 2J,K).

Quantification of viscosity revealed that 3K αSyn condensates formed in the presence of oleic acid exhibited significantly higher viscosity than condensates formed without oleic acid (*P* = 0.0307) (Fig. 2L). Thus, oleic acid shifts 3K αSyn condensates toward a more viscous, less fluid material state. This result is consistent with the FRAP data showing that oleic acid treatment increases the immobile fraction of 3K αSyn condensates, indicating a transition toward a more solid-like or less dynamic condensate state. Together, these data support the conclusion that lipid-droplet-rich 3K αSyn condensates display increased resistance to flow under aspiration.

### Lipid droplets modulate the number, type and S129 phosphorylation status of αSyn condensates

We sought to determine whether there is a dose response relationship between the oleic acid concentration in the culture media, the amount of lipid droplets and the number, size and type of αSyn condensates. We induced the formation of lipid droplets using two orthogonal methods: treatment with oleic acid or linoleic acid. We used the same concentrations and serial dilutions for each to enable robust comparisons of the results. Oleic acid and linoleic acid induce the formation of lipid droplets through separate mechanisms. Oleic acid is a monounsaturated omega-9 fatty acid; it is esterified and converted into triglycerides that incorporate into lipid droplets (Nakajima et al, 2019), or binds to the FFAR4 receptor on the cell surface that activates a signaling pathway including phosphoinositide 3-kinase and phospholipase D, which leads to the formation of new lipid droplets (Rohwedder et al, 2014). On the other hand, linoleic acid is a polyunsaturated, omega-6 fatty acid that is activated through esterification (Whelan and Fritsche, 2013) and incorporates into neutral lipids that are synthesized in the endoplasmic reticulum from which the lipid droplets eventually bud off.

**Figure 3:**
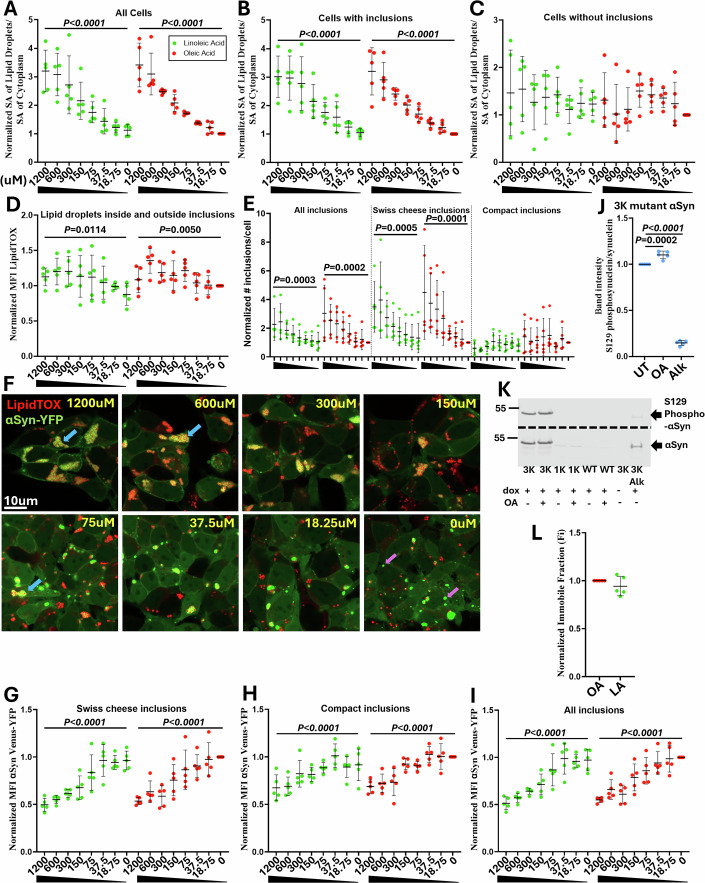
Lipid droplets modulate 3 K αSyn condensates. (**A**–**C**) Dose response experiments for Linoleic acid (LA) and oleic acid (OA) showed that higher concentrations are significantly associated with an increased surface area (SA) of lipid droplets relatively to the total surface area of the cytoplasm, and that this association is driven by the cells containing αSyn condensates. The same concentrations were used in all dose response experiments for both lipids. One-way ANOVA with test for linear trend. (**D**) Dose response experiments for the mean fluorescence intensity (MFI) of the lipid droplets stained through LipidTOX far red showed that the MFI significantly increased as the concentrations of LA and OA increased. One-way ANOVA with test for linear trend. (**E**) Dose response experiments for LA and OA showed that the number of Swiss cheese condensates significantly increased with higher concentrations of LA and OA. There was no effect on the number of compact αSyn condensates. The same OA and LA concentrations were used as in (**A**–**C**). One-way ANOVA with test for linear trend. (**F**) Representative confocal images for cells treated with the dose response of OA. Shown in blue arrows are Swiss cheese and in purple arrows compact αSyn condensates. (**G**–**I**) The MFI of αSyn condensates (Swiss cheese (**G**), compact (**H**) and all condensates (**I**)) significantly decreased as the concentration of OA and LA increased. One-way ANOVA with test for linear trend. (**J**, **K**) Western blot for S129 phosphorylated αSyn and total αSyn. 600 μM of OA treatment for 24 h at 2 days after doxycycline induction significantly increased the S129 phosphorylation of 3K αSyn. Alkaline phosphatase (Alk) was used as a positive control. 1K and WT αSyn show no phosphorylation consistent with our previous report (Eubanks et al, 2025). UT indicates cells without OA treatment. One-way ANOVA with Tukey’s post hoc correction. (**L**) FRAP experiments for 3K αSyn formed after treatment with 600 μM of LA or OA showed no difference in their immobile fractions (Fi). One sample *t* test. Concentrations in all plots are in μM. LA: green dots, OA: red dots. All experiments were repeated independently five times, and the normalized values are shown in each plot. Data are represented as mean +/− SD. SA surface area.

We studied this process on 3K αSyn. Higher concentrations of oleic acid and linoleic acid resulted in a significantly increased amount of lipid droplets as determined by the quantification of their surface area in relation to the total surface area of the cell within the same optical section; there was no difference between the same concentrations for these two treatments (Fig. 3A). This dose response effect was seen only for cells that contained αSyn condensates (Fig. 3B), which drove the results seen for the entire population of cells (Fig. 3A), but not for cells without (Fig. 3C). In addition, higher concentrations of linoleic acid and oleic acid correlated with increased fluorescence intensity of LipidTOX (staining neutral lipids within lipid droplets) (Fig. 3D). These results indicate that oleic acid and linoleic acid induce the formation of lipid droplets in a dose response manner and that there is a relationship between the amount of lipid droplets and the presence of condensates. In other words, it indicates that lipid droplets facilitate the formation of αSyn condensates.

To further corroborate the latter point, we quantified the number and type of αSyn condensates. As we previously showed, 3K αSyn condensates are of two different types: Swiss cheese-like that incorporate lipid droplets, and compact that do not (Eubanks et al, 2025). Our results indicate that both treatments significantly increase the total number of αSyn condensates and that this is driven by the increase in the number of Swiss cheese condensates; there is no difference seen for the compact condensates (Fig. 3E,F). Finally, higher concentrations of linoleic acid and oleic acid correlated with lower fluorescence intensity of αSyn-YFP within Swiss cheese (Fig. 3G) and compact condensates (Fig. 3H). The same results were observed when considering all types of inclusions together (Fig. 3I). These results confirm that oleic and linoleic acid, and consequently lipid droplets, induce the formation of αSyn condensates and there is no difference in the efficiency associated with either treatment. The increase in the number of αSyn condensates is accompanied by decrease in their fluorescence intensity, indicating that the same total amount of αSyn is distributed among a larger number of condensates.

**Figure EV1:**
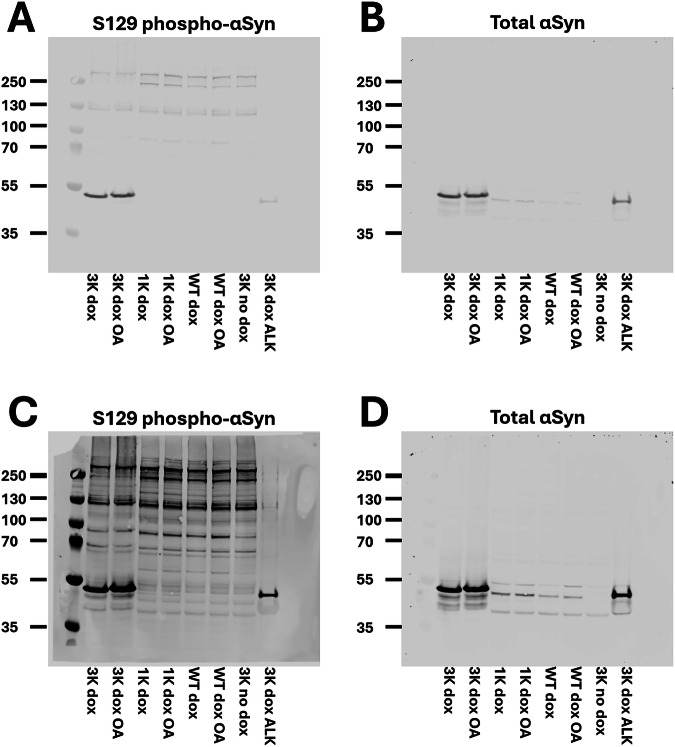
Oleic acid increases S129 phosphorylation of αSyn. (**A**, **B**) Uncropped versions of the Western blots in Fig. 3K. (**C**, **D**) Higher-exposure versions of the same western blots, demonstrating the presence of total αSyn bands in WT and 1K cells and confirming the absence of a detectable αSyn-YFP band in 3K cells without doxycycline (dox) induction. WT and 1K αSyn do not show a detectable band corresponding to S129 phosphorylation under these experimental conditions, although phosphorylation levels may be below the detection limit of the assay. The 3K αSyn bands exhibit reduced electrophoretic mobility, consistent with the extensive S129 phosphorylation previously reported for this variant (Eubanks et al, 2025; Imberdis et al, 2019; Ramalingam et al, 2023a). Total αSyn protein levels are higher in 3K cells than in WT and 1K cells. OA, oleic acid. (Supplementary Fig. 1 in BioImage Archive repository).

We assessed the S129 phosphorylation status of the αSyn condensates, as it is the most widely used marker for pathological αSyn and particularly αSyn aggregation. However, whether S129 phosphorylation induces aggregation, is a secondary event following aggregation, or inhibits further aggregation and toxicity is controversial (Ghanem et al, 2022; Oueslati, 2016). Treatment with oleic acid significantly increased the Fi of 3K αSyn condensates, thereby making them more solid (Fig. 2A,B). It also resulted in a small but significant increase in 3K αSyn S129 phosphorylation (Figs. 3J,K and EV1A–D). These findings suggest that increased lipid droplets induce the transformation of αSyn into a pathological S129 phosphorylated form. It is possible that lipid droplets act as a nidus on which αSyn binds (Eubanks et al, 2025) and congregates, resulting in its aggregation and secondary phosphorylation due to locally increased concentration. The pS129 αSyn signal detected in WT and 1K cells is probably approaching the sensitivity limits of the assay. While it is therefore difficult to definitively distinguish between absence of phosphorylation and levels below detection, this observation is consistent with prior reports indicating that S129 phosphorylation is enriched in the 3K variant (Imberdis et al, 2019; Ramalingam et al, 2023a) and in more aggregated or pathological αSyn states, which are exemplified by the 3K variant in this work.

FRAP experiments showed that the Fi of αSyn condensates formed after treatment with 600 μM of either linoleic acid or oleic acid was the same (Fig. 3L). This indicates that comparable levels of lipid droplets, regardless of their path of induction and formation, result in the formation of αSyn condensates of similar material properties.

### Lipid droplets promote the formation of reversible αSyn condensates

**Figure EV2:**
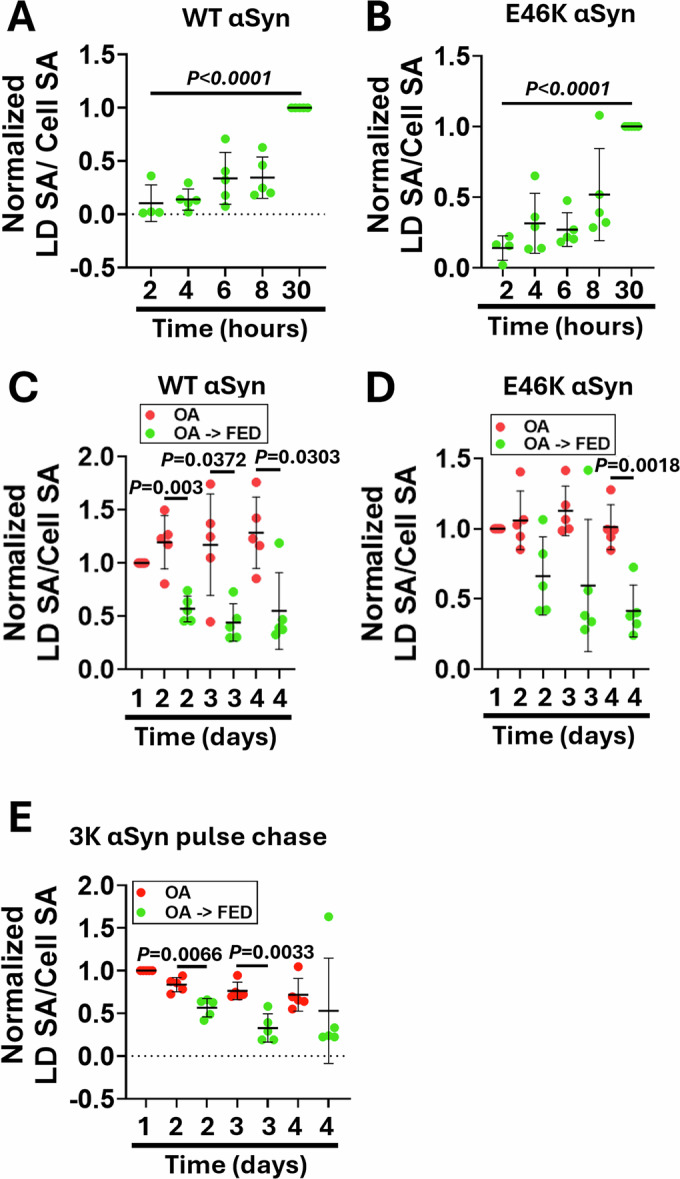
Lipid droplets reduce over time in an oleic acid pulse-chase experiment. (**A**, **B**) WT and 1K cells were induced with doxycycline and imaged at 2, 4, 6, 8 and 30 h post-induction to quantify the surface area (SA) of lipid droplets in relation to the surface area of the cell. One-way ANOVA with test for linear trend. (**C**–**E**) WT, 1K and 3K cells were induced with doxycycline for 48 h, treated with oleic acid (OA) for 24 h and imaged (day 1); subsequently, their media was replaced with media without OA and imaged every 24 h over 3 days. The surface area of lipid droplets was quantified in relation to the surface area of the cytoplasm. Unpaired *t* test with Bonferroni correction. All experiments were repeated independently five times, and the normalized values are shown in each plot. Data are represented as mean +/− SD. (Supplementary Fig. 2 in BioImage Archive repository).

**Figure 4:**
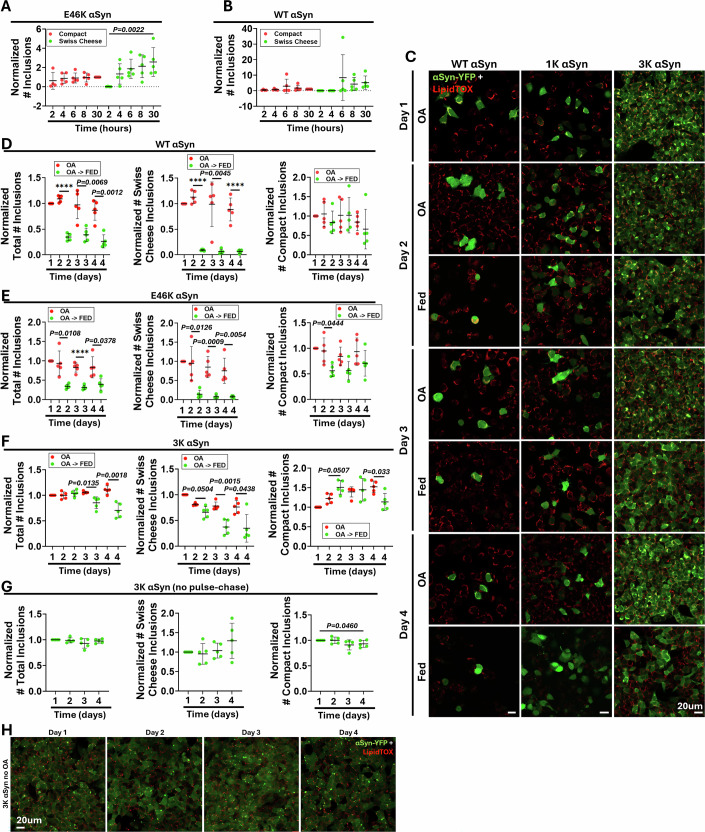
Lipid droplets promote the formation of reversible αSyn condensates. (**A**, **B**) Timecourse experiment in which 1K (**A**) and WT (**B**) cells were imaged at 2, 4, 6, 8 and 30 h post-doxycycline induction to quantify αSyn inclusions and lipid droplets. One-way ANOVA with test for linear trend. (**C**) Representative images of a pulse chase experiment in which WT, 1K and 3K cells were induced with doxycycline for 48 h, pulsed in 600 μM oleic acid (OA) for 24 h and imaged (day 1), and subsequently chased in media without oleic acid over an additional 3 days. As a comparison, cells that were maintained in oleic acid-supplemented media were imaged in parallel. (**D**–**F**) Compact, Swiss cheese and total αSyn condensates were quantified over 4 days in the cells that were treated with oleic acid and chased in media without oleic acid (OA- > FED), as well as in cells that were maintained in oleic acid-supplemented media throughout (OA). Unpaired *t* tests with Bonferroni correction. (**G**) 3K αSyn cells were imaged over 4 days without oleic acid supplementation. One-way ANOVA with test for linear trend. (**H**) Representative images for (**G**). All experiments were repeated independently five times, and the normalized values are shown in each plot. Data are represented as mean +/− SD. *P* value: ****≤0.0001.

To examine the relationship between lipid droplets and αSyn inclusions at early stages of formation, we performed a time-course experiment over 30 h following doxycycline induction. WT and 1K αSyn-expressing cells were imaged at 2, 4, 6, 8, and 30 h. Both cell lines exhibited a progressive increase in lipid droplets over time (Fig. EV2A,B). In parallel, 1K αSyn showed a significant increase in lipid droplet-rich (“Swiss cheese”) inclusions (Fig. 4A), whereas WT αSyn showed a similar trend that did not reach significance (Fig. 4B). In contrast, lipid droplet-poor (“compact”) inclusions remained unchanged over time (Fig. 4A,B). These findings indicate that increasing lipid droplet abundance is associated with the preferential formation of lipid droplet-rich αSyn inclusions, with a stronger effect observed for the more positively charged 1K variant. While we did not directly assess the material state of these early inclusions, at later timepoints, these assemblies exhibit condensate-like properties based on FRAP and MAPAC measurements (Fig. 2A,B,L).

To test whether αSyn condensates are reversible, we performed a pulse-chase experiment. Cells were treated with oleic acid for 24 h (pulse; day 1), followed by replacement with oleic acid-free media and imaging every 24 h for an additional 3 days (chase; days 2–4). Parallel conditions included cells continuously maintained in oleic acid, as well as 3K αSyn-expressing cells without oleic acid treatment. Upon withdrawal of oleic acid, lipid droplet abundance progressively decreased over 4 days in all 3 cell lines (Fig. EV2C–E).

Consistent with this reduction, the total number of αSyn condensates declined significantly over time in WT and 1K cells. This decrease was primarily driven by a reduction in Swiss cheese condensates, whereas compact condensates remained relatively stable (Fig. 4C–E). In 3K-expressing cells, the decrease in total condensates was more modest and delayed, becoming apparent mostly at days 3 and 4, and was similarly driven by loss of Swiss cheese condensates (Fig. 4C,F). In contrast, 3K αSyn in the absence of oleic acid remained largely stable over the 4-day period, with only a minor reduction in compact condensates (Fig. 4G,H).

Together, these results demonstrate that αSyn condensates are reversible and dynamically regulated by lipid droplet availability, supporting a causal relationship in which lipid droplets promote the formation and maintenance of αSyn condensates.

### αSyn condensates induce local mitochondrial depolarization

**Figure 5:**
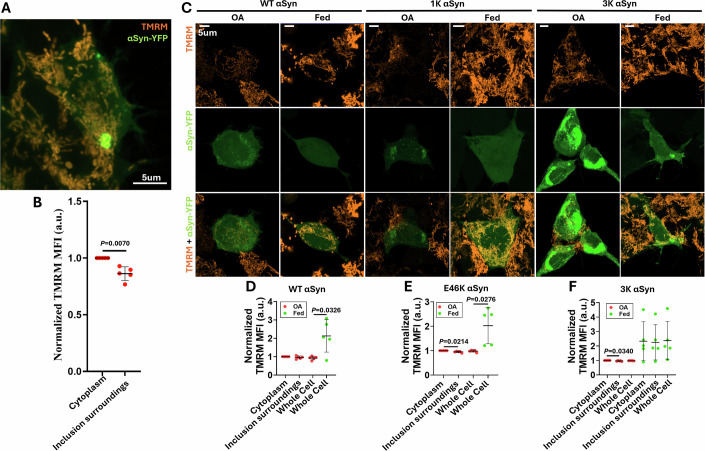
αSyn condensates induce local mitochondrial depolarization. (**A**) Multiple intensity projection image generated from z-stack images of M17D cells expressing 3K αSyn-YFP showing that mitochondria closely approximate αSyn condensates. (**B**) Mean fluorescence intensity (MFI) of tetramethylrhodamine methyl ester (TMRM) showing that mitochondria located within a 7-pixel radius around 3K αSyn condensates are significantly depolarized. One-sample *t* test. a.u. arbitrary units. Mitochondrial membrane potential was measured at 48 h post-doxycycline induction. (**C**) Representative maximum intensity projection images generated from z-stacks of WT, 1K, and 3K cells stained with TMRM. Cells were imaged on day 4 of the pulse-chase experiment shown in Fig. 4C–F (i.e., 4 days post-oleic acid treatment). (**D**, **E**) Mitochondrial membrane potential was quantified by measuring the mean fluorescence intensity (MFI) of TMRM in WT and 1K cells chased in media without oleic acid (OA) compared to cells maintained in OA. Imaging and quantification were performed on day 4 of the pulse-chase experiment (Fig. 4C–F). In the absence of OA, most αSyn condensates were lost, resulting in sparse inclusions that precluded localized measurements of mitochondrial membrane potential. Therefore, TMRM MFI was quantified at the whole-cell level and compared across conditions. Unpaired *t* tests with Bonferroni correction. (**F**) Mitochondrial membrane potential was measured in 3K cells on day 4 of the pulse-chase experiment. Measurements were undertaken at the whole cell level and around condensates in both conditions. Unpaired *t* tests with Bonferroni correction. All experiments were repeated independently five times, and the normalized values are shown in each plot. Data are represented as mean +/− SD.

In our previous work, we showed that overexpression of 3K αSyn induces mitochondrial depolarization (Eubanks et al, 2025), consistent with extensive evidence linking αSyn dysregulation to mitochondrial dysfunction (Choi et al, 2022; Ludtmann et al, 2018). Here, we observed that mitochondria closely surround 3K αSyn condensates (Fig. 5A), raising the possibility that these assemblies directly perturb mitochondrial function.

To test this, we measured mitochondrial membrane potential (Δψ) in live cells using tetramethylrhodamine methyl ester (TMRM) in redistribution mode. Z-stack imaging was performed, and Δψ was quantified locally within a defined region surrounding αSyn condensates (7-pixel radius) and compared to the remaining cytoplasm (Esteras et al, 2017). Mitochondria in close proximity to 3K αSyn condensates were significantly depolarized relative to those located elsewhere in the cytoplasm (Fig. 5B), indicating a spatially restricted effect.

**Figure EV3:**
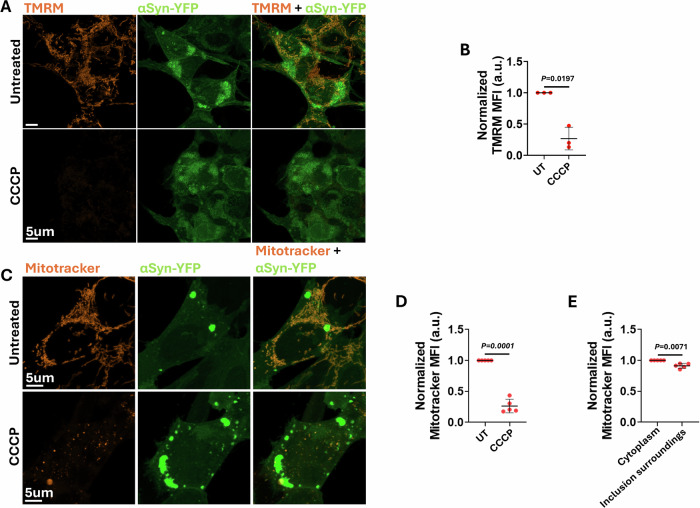
Validation of mitochondrial experiments. (**A**, **B**) Treatment of 3K cells with carbonyl cyanide m-chlorophenyl hydrazone (CCCP) results in mitochondrial depolarization, as quantified by the reduction of the mean fluorescence intensity (MFI) of tetramethylrhodamine methyl ester (TMRM). One-sample *t* test. (**C**, **D**) MFI of MitoTracker CMXRos was used to measure the mitochondrial membrane potential. Treatment with CCCP resulted in a significant reduction in MitoTracker CMXRos MFI. One-sample *t* test. (**E**) MitoTracker CMXRos showed reduced MFI in a 7-pixel radius around 3K αSyn condensates. One-sample *t* test. All experiments were repeated independently five times, and the normalized values are shown in each plot. Data are represented as mean +/− SD. a.u. arbitrary units. (Supplementary Fig. 3 in BioImage Archive repository).

To validate the robustness of our measurements, we used carbonyl cyanide m-chlorophenyl hydrazone (CCCP), a mitochondrial uncoupler, as a positive control. CCCP treatment led to a marked reduction in Δψ, confirming the sensitivity of the TMRM assay (Fig. EV3A,B). As a secondary validation of our membrane potential measurements, we employed an independent approach using MitoTracker CMXRos (Alam et al, 2022). This dye similarly demonstrated CCCP-induced depolarization and confirmed that mitochondria adjacent to 3K αSyn condensates exhibit reduced membrane potential (Fig. EV3C–E). Together, these controls establish the reliability of our mitochondrial measurements.

We next extended this analysis to WT and 1K αSyn and compared them to 3K. Cells were induced with doxycycline for 48 h, followed by oleic acid treatment for 24 h, and Δψ was measured 96 h (4 days) later. Parallel conditions included cells subjected to oleic acid withdrawal (pulse-chase) (representative images are shown in Fig. 5C, WT αSyn in Fig. 5D, 1K αSyn in Fig. 5E and 3K αSyn in Fig. 5F). At the whole-cell level, withdrawal of oleic acid led to recovery of mitochondrial membrane potential across all three variants. In contrast, under continuous oleic acid treatment, mitochondria surrounding αSyn condensates in 1K and 3K cells were significantly depolarized compared to the surrounding cytoplasm. This localized depolarization was not observed in WT cells. Notably, in 3K cells subjected to oleic acid withdrawal, residual condensates persisted; however, mitochondria in their vicinity were no longer significantly depolarized (Fig. 5C–F).

These findings indicate that αSyn condensates can induce localized mitochondrial depolarization, with a stronger effect observed for the more positively charged 1K and 3K variants. This suggests a direct interaction between condensates and mitochondria, potentially mediated by the release of toxic αSyn species. While WT condensates do not produce a comparable local effect, all three variants exhibit some degree of global mitochondrial depolarization at the cellular level. Importantly, both condensate burden and mitochondrial dysfunction are reversible upon lipid droplet depletion (Figs. 4D–F and 5C–F). Due to the near-complete loss of condensates in WT and 1K cells following oleic acid withdrawal, recovery of local mitochondrial polarization could only be assessed in 3K cells.

Finally, we note that our analysis does not distinguish between lipid droplet-rich and lipid droplet-poor condensates, and therefore reflects the overall impact of αSyn condensates on mitochondrial function.

### αSyn condensates promote localized mitophagy

Damaged mitochondria are selectively removed via mitophagy. Based on our observation that mitochondria surrounding 1K and 3K αSyn condensates are locally depolarized, we hypothesized that these mitochondria would undergo increased mitophagy compared to those associated with WT αSyn condensates.

**Figure 6:**
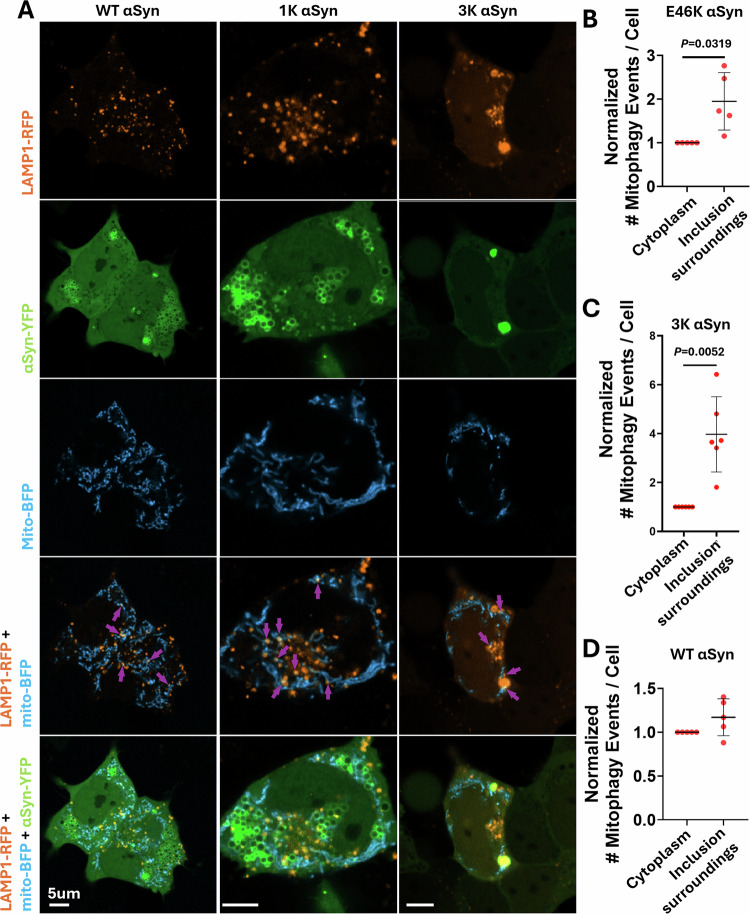
αSyn condensates promote localized mitophagy. (**A**) Cells were transfected with mito-BFP and LAMP1-RFP constructs to visualize the mitochondria and lysosomes, respectively. Late mitophagy events were determined by the colocalization of RFP with BFP. (**B**–**D**) Mitophagy events were significantly more frequent within a 25-pixel radius around 1K (**B**) and 3K (**C**) αSyn condensates than in the remaining cytoplasm, but there was no difference seen for WT condensates (**D**). Mitophagy events are indicated with purple arrows. One-sample *t* tests. All experiments were repeated independently five times for WT and 1K cells and six times for 3K cells, and the normalized values are shown in each plot. Data are represented as mean +/− SD. a.u. arbitrary units.

To test this, we quantified mitophagy by measuring colocalization events between lysosomes and mitochondria following co-transfection with LAMP1-RFP and mito-BFP, respectively. This approach captures mitophagy events independent of specific pathway markers (Berezhnov et al, 2016). We compared mitochondria within a 25-pixel radius of αSyn condensates to those in the remaining cytoplasm. Mitochondria proximal to 1K (Fig. 6A,B) and 3K (Fig. 6A,C) αSyn condensates exhibited significantly higher levels of mitophagy compared to distal mitochondria, whereas no such difference was observed for WT αSyn condensates (Fig. 6A,D). Mitophagy events were observed both in proximity to αSyn condensates and throughout the cytoplasm, indicating that mitophagy is not exclusively spatially restricted to inclusion-containing regions. However, quantitative analysis revealed a significant enrichment of mitophagy events in the vicinity of αSyn condensates compared to distal cytoplasmic regions.

These findings are consistent with our TMRM measurements, which showed localized mitochondrial depolarization around 1K and 3K but not WT αSyn condensates (Fig. 5C–F), supporting the conclusion that 1K and 3K condensates induce mitochondrial damage that triggers mitophagy.

**Figure EV4:**
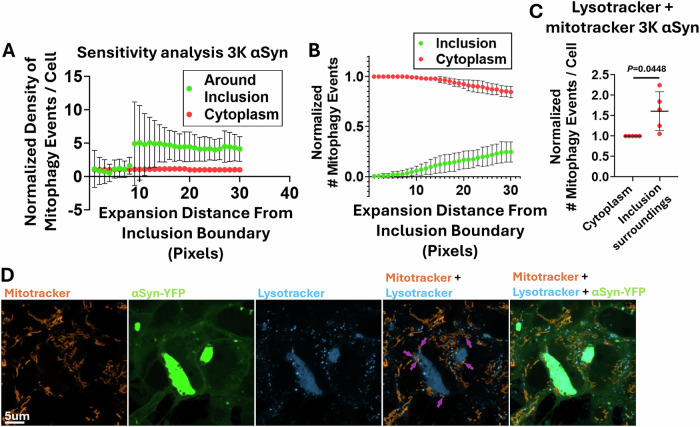
Validation of mitophagy experiments. (**A**) The mitophagy images from Fig. 6C (3K cells) were re-analyzed in sensitivity format. αSyn condensates were dilated by 1-pixel radius at a time. Mitophagy events were quantified within the dilated rim surrounding the condensates as well as in the remaining cytoplasm. The number of mitophagy events was normalized to the surface area (SA) of the dilated rim or the cytoplasm, respectively. No statistical analysis was undertaken. 0.071 µm/pixel. (**B**) The data from (**A**) are shown without normalization to surface area. (**C**, **D**) Live 3K cells were stained with Lysotracker Blue and Mitotracker red, followed by colocalization analysis to identify mitophagy events using the same workflow as in Fig. 6. Bleedthrough was present from the green channel (αSyn-YFP) to the blue channel (Lysotracker), which was subtracted through Python code during analysis. Mitophagy events are shown in purple arrows. One-sample *t* test. All experiments were repeated independently six times for the sensitivity analysis on the 3K cells and five times for lysotracker+mitotracker experiment, and the normalized values are shown in each plot. Data are represented as mean +/− SD. (Supplementary Fig. 4 in BioImage Archive repository).

To validate our spatial analysis, we performed a sensitivity assessment of the radial cutoff. Quantification of mitophagy events around 3K αSyn condensates across radii ranging from 1 to 30 pixels revealed a plateau beginning at ~9 pixels, confirming that our 25-pixel radius provides a robust and inclusive measure of local effects (Fig. EV4A,B). As an independent validation, we used Lysotracker and Mitotracker staining and observed significantly increased colocalization around 3K αSyn condensates compared to the surrounding cytoplasm (Fig. EV4C,D), consistent with results obtained using the LAMP1-RFP/mito-BFP system.

### Mitochondrial damage precedes mitophagy

Two mechanisms could explain the observed coupling between mitochondrial depolarization and increased mitophagy near αSyn condensates. In one scenario, αSyn condensates, by virtue of their dynamic, viscous nature, release toxic αSyn species that directly impair mitochondrial function, leading to depolarization and subsequent mitophagy. Alternatively, lysosomes may target αSyn condensates for degradation and, in doing so, nonspecifically engulf nearby mitochondria, resulting in secondary mitochondrial damage.

**Figure EV5:**
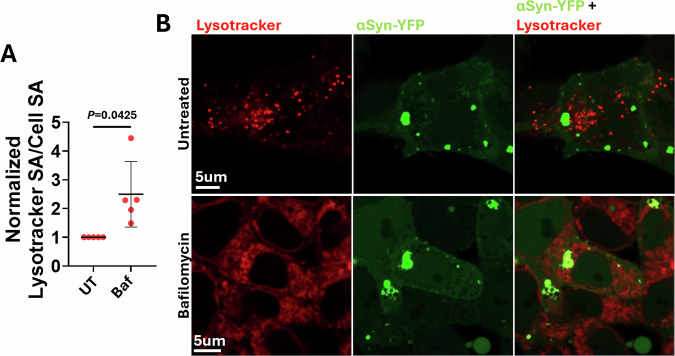
Bafilomycin A1 treatment increases the lysosomal area. (**A**, **B**) 3K cells were treated with 100 nM of Bafilomycin A1 for 24 h and stained with Lysotracker Deep Red to visualize the lysosomes. The surface area (SA) of the lysosomes was divided with the SA of the cells on a cell-by-cell basis. Comparison was made between Bafilomycin-treated and untreated cells. One-sample *t* test. Data are represented as mean +/− SD. Baf=Bafilomycin A1. (Supplementary Fig. 5 in BioImage Archive repository).

**Figure 7:**
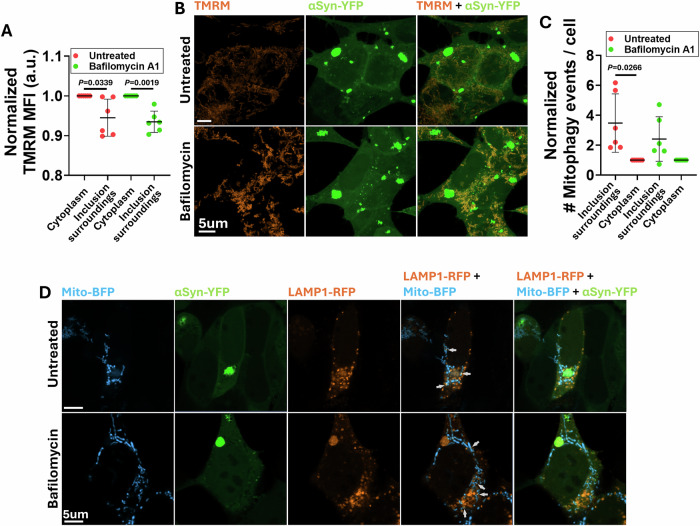
Mitochondrial damage precedes mitophagy. (**A**, **B**) 3K cells were treated with 100 nM of Bafilomycin A1 for 24 h and compared to untreated cells that were stained with tetramethylrhodamine methyl ester (TMRM). The mean fluorescence intensity (MFI) of TMRM was quantified in the region surrounding the αSyn condensates and in the remaining cytoplasm. One-sample *t* tests. (**C**, **D**) 3K cells were transfected with Mito-BFP and LAMP1-RFP. Mitophagy events were quantified in the region surrounding the αSyn condensates and in the remaining cytoplasm. One-sample *t* tests. All experiments were repeated independently six times, and the normalized values are shown in each plot. Data are represented as mean +/− SD. a.u. arbitrary units.

To distinguish between these possibilities, we treated 3K αSyn-expressing cells with Bafilomycin A1, an inhibitor of autophagic flux. Bafilomycin treatment led to a significant enlargement of lysosomal compartments, as quantified by Lysotracker staining, confirming effective pathway inhibition (Fig. EV5A,B). Under these conditions, mitochondria in proximity to αSyn condensates remained significantly depolarized (Fig. 7A,B). In contrast, the elevated mitophagy observed near condensates was abolished by Bafilomycin treatment (Fig. 7C,D).

These findings indicate that mitochondrial depolarization occurs independently of mitophagy and therefore precedes it. If lysosome-driven off-target degradation were the primary cause of mitochondrial damage, inhibition of autophagy would be expected to rescue both mitophagy and mitochondrial depolarization. Instead, the persistence of depolarization despite blocked mitophagy supports a model in which αSyn condensates directly induce mitochondrial damage, followed by secondary clearance through mitophagy.

**Figure 8:**
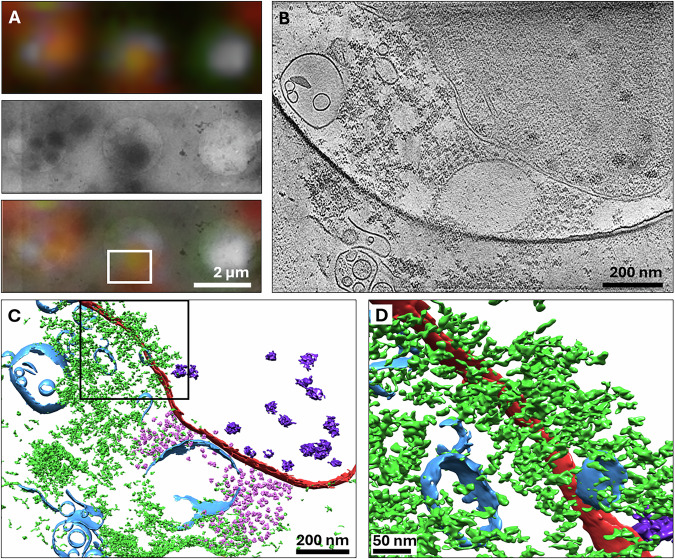
Correlative light and electron microscopy (CLEM) and cryo-electron tomography (cryo-ET) reveal direct interactions between αSyn and the mitochondrial outer membrane in cell lysates from 3K M17D cells. (**A**) Cryo-fluorescence (top), low-magnification cryo-EM (middle), and correlated overlay (bottom) images of αSyn (green) and mitochondria (red) on EM grids of cell lysates. (**B**, **C**) Tomographic slice (**B**) and 3D annotated (**C**) views of the boxed region in (**A**), showing αSyn associated with the mitochondrial membrane and inducing membrane deformation. (**D**) Zoomed-in and tilted view of the boxed region in (**C**), highlighting the mitochondrial membrane and sites of αSyn association. The mitochondrial membrane is shown in red, αSyn in green, intramitochondrial granules in purple, other vesicular structures in blue, and ribosomes in pink.

**Figure EV6:**
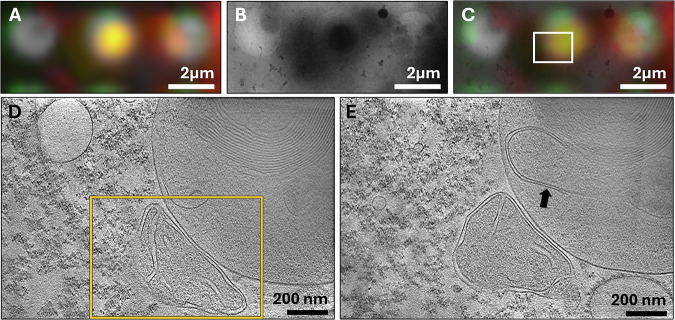
Correlative light and electron microscopy (CLEM) and cryo-electron tomography (cryo-ET) reveal intensive direct interactions between αSyn structures and the mitochondrial outer membrane in cell lysates from 3K M17D cells. (**A**–**C**) CLEM analysis showing cryo-fluorescence (**A**), low-magnification cryo-EM (**B**), and correlated overlay (**C**) of αSyn (green) and mitochondria (red) in cell lysates from 3K M17D cells. (**D**) Slice view of a tomogram from the boxed area in (**C**), depicting αSyn association on the outer membrane of a mitochondrion and inducing pronounced morphological alterations (orange box). (**E**) A different slice from the same tomogram, highlighting a multivesicular body enclosing a mitochondrion (black arrow).

**Figure EV7:**
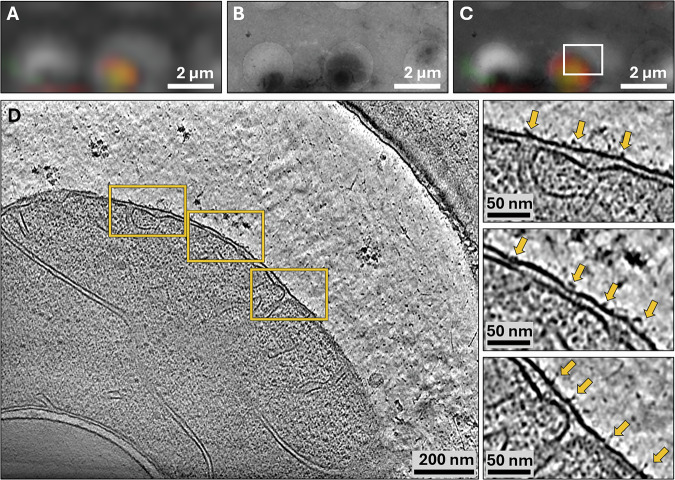
CLEM and cryo-ET shows association of small putative αSyn species on the outer mitochondrial membrane. (**A**–**C**) CLEM analysis showing cryo-fluorescence (**A**), low-magnification cryo-EM (**B**), and correlated overlay (**C**) of αSyn (green) and mitochondria (red) in cell lysates from 3K M17D cells. (**D**) Slice view of a tomogram from the boxed area in (**C**), showing smaller αSyn species, suggestive of oligomers, associated on the outer membrane of a mitochondrion. Boxed insets highlight zoomed-in regions with αSyn species and areas of mitochondrial membrane roughness (orange arrows).

**Figure EV8:**
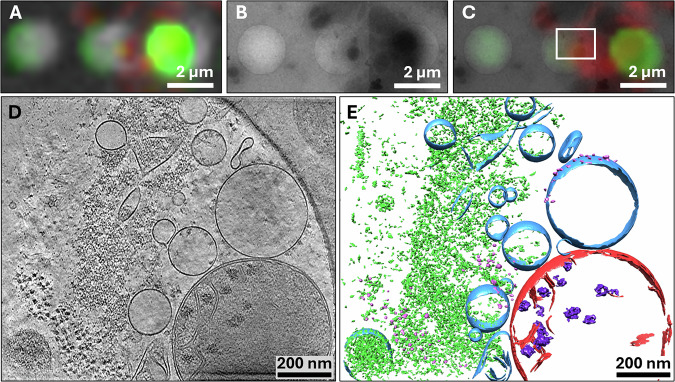
CLEM and cryo-ET reveal αSyn species and mitochondrial structures in cell lysates from 3K M17D cells. (**A**–**C**) CLEM analysis showing cryo-fluorescence (**A**), low-magnification cryo-EM (**B**), and correlated overlay (**C**) of αSyn (green) and mitochondria (red) in cell lysates from 3K M17D cells. The image in (**B**) is a stitched tiles of low-magnification EM images. The vertical line represents the boundary between the two images. (**D**, **E**) Tomographic slice (**D**) and 3D annotated view (**E**) of a tomogram from the boxed region in (**C**), showing an αSyn network in proximity to a mitochondrion. The αSyn network does not directly interact with the mitochondrial membrane. The mitochondrion exhibits a representative smooth, spherical morphology. Cellular vesicular structures embedded within the αSyn network display signs of membrane deformation. The mitochondrial membrane is shown in red, αSyn in green, intramitochondrial granules in purple, other vesicular structures in blue, and ribosomes in pink.

To further probe this mechanism, we performed correlative light and electron microscopy (CLEM) and cryo-electron tomography (cryo-ET) on 3K αSyn samples as previously described (Eubanks et al, 2025). αSyn condensates were identified via their YFP signal, and mitochondria were labeled with Mitotracker for CLEM studies. Tilt series were collected from regions where the two signals were in close proximity or overlapped. In tomograms of cell lysate, αSyn appears as networks of amorphous structures, suggestive of higher-order assemblies of oligomers. These networks were often observed with ribosomes interspersed or entangled within them, suggesting potential direct interactions between the two. Mitochondria in direct contact with these large αSyn networks often exhibited clear morphological alterations, including irregular membrane contours and deviations from typical mitochondrial morphology (Figs. 8A–D and EV6A–E; Movie EV1). Mitochondria were also observed enclosed within multivesicular body-like compartments. In some tomograms, mitochondria were observed with smaller, putative αSyn oligomeric species attached to the outer membrane. These attachments were often associated with pronounced membrane roughness, and in some cases, localized membrane disruption (Fig. EV7A–D). In contrast, mitochondria without direct interactions with αSyn condensates retained intact morphology (Fig. EV8; Movie EV2).

Together, these data support a model in which αSyn condensates generate or concentrate toxic αSyn species that associate with mitochondrial membranes, causing structural damage and depolarization. This primary mitochondrial injury subsequently triggers mitophagy. Due to the higher abundance and brightness of condensates in 3K-expressing cells, CLEM analysis was feasible only in this condition.

### αSyn condensates entrap lipid droplets and impair their turnover

We and others have shown that αSyn condensates frequently entrap lipid droplets (Ericsson et al, 2021; Eubanks et al, 2025). Lipid droplets play a key role in cellular energy homeostasis by storing neutral lipids and releasing fatty acids through lipophagy for mitochondrial utilization under energy demand (Rambold et al, 2015; Singh et al, 2009). We therefore hypothesized that sequestration of lipid droplets within αSyn condensates impairs their turnover and limits their contribution to cellular energy metabolism.

To test this, we employed a pulse-chase assay to monitor lipid droplet turnover (Rambold et al, 2015). Cells were pulsed overnight with BODIPY 558/568 C12, a fluorescent fatty acid analogue that incorporates into lipid species. The following day, cells were chased overnight in dye-free media containing 600 μM oleic acid (WT, 1K, and 3K) or without oleic acid (3K only), and subsequently stained with LipidTOX Far Red prior to imaging. This approach enables tracking of C12 redistribution and lipid remodeling following withdrawal from the media.

**Figure 9:**
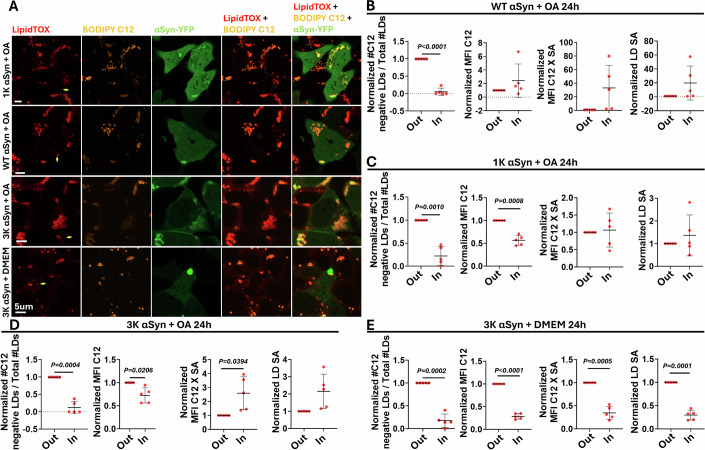
αSyn condensates entrap lipid droplets and reduce their turnover. (**A**–**E**) A BODIPY C12 pulse-chase assay was used to quantify the turnover of lipid droplets inside (In) and outside (Out) of αSyn condensates. Lipid droplets were detected through LipidTOX Deep Red staining. Several parameters were quantified: number of C12+ lipid droplets, total number of lipid droplets (LipidTOX + ), mean fluorescence intensity (MFI) of C12, surface area (SA) of lipid droplets, and were used to compute the following metrics, all on a cell-by-cell basis, only for the cells expressing αSyn: Number of C12+ lipid droplets/total number of lipid droplets, MFI of C12, integrated MFI of C12 (C12 MFI multiplied with surface area of lipid droplets). Lipid droplets that are not entrapped in αSyn condensates show a loss of BODIPY 558/568 C12 staining more frequently than lipid droplets that are entrapped in WT (**B**), 1K (**C**) and 3K (**D**, **E**) αSyn condensates chased in media supplemented with oleic acid (OA) (**B**–**D**) or not (**E**). Yellow arrows indicate lipid droplets that have lost C12 staining. One-sample *t* tests. All experiments were repeated independently five times, and the normalized values are shown in each plot. Data are represented as mean +/− SD.

Lipid droplets within αSyn condensates were consistently double-positive for C12 and LipidTOX, whereas cytoplasmic lipid droplets frequently lost C12 labeling over time. This pattern was observed across WT (Fig. 9A,B), 1K (Fig. 9A,C), and 3K αSyn (Fig. 9A,D,E). Notably, the mean fluorescence intensity (MFI) of C12 in lipid droplets entrapped within condensates was significantly reduced compared to cytoplasmic droplets in 1K (Fig. 9A,C) and 3K cells (Fig. 9A,D,E), but not in WT (Fig. 9A,B). This indicates that droplets within condensates are relatively homogeneous, retaining low but uniform C12 signal, whereas cytoplasmic droplets are more heterogeneous, with subsets showing either strong retention or complete loss of C12. These findings are consistent with active turnover of cytoplasmic lipid droplets, in contrast to impaired turnover of those sequestered within condensates.

Consistent with this, in 3K cells maintained in oleic acid, the integrated C12 signal (MFI × droplet surface area) was significantly higher in lipid droplets within condensates compared to those in the cytoplasm, reflecting preferential accumulation and retention. This effect was less pronounced in 1K (Fig. 9A,C) and WT cells (Fig. 9A,B), suggesting a stronger sequestration phenotype in the more highly charged 3K variant (Fig. 9A,D). In contrast, 3K cells chased in the absence of oleic acid showed a reversal of this pattern, consistent with reduced lipid droplet availability and altered condensate association dynamics (Fig. 9A,E).

Overall, across all conditions examined, lipid droplets entrapped within αSyn condensates consistently differed from those in the cytoplasm in both C12 retention and fluorescence intensity. These results support a model in which αSyn condensates sequester lipid droplets and impair their normal turnover, potentially limiting lipid mobilization for cellular energy needs. WT and 1K cells were not analyzed under oleic acid-free pulse conditions, as they do not form inclusions in the absence of lipid droplet induction.

## Discussion

**Figure 10:**
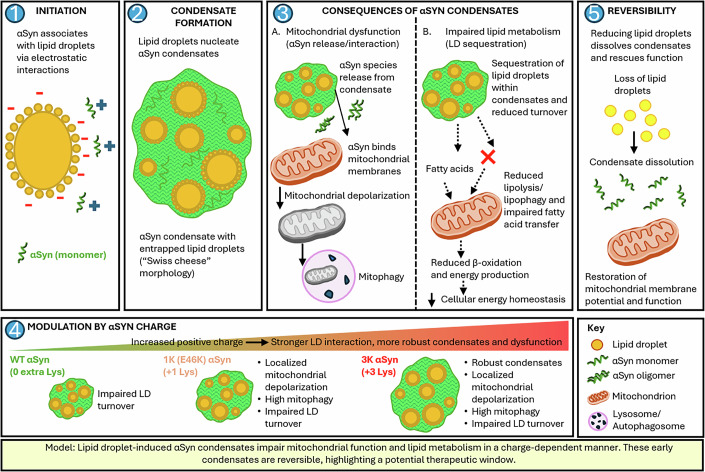
Proposed model for lipid-driven αSyn condensates and organelle dysfunction. αSyn associates with lipid droplets through electrostatic interactions, triggering aberrant phase separation and the formation of biomolecular condensates. Lipid droplets nucleate the formation of αSyn condensates and get entrapped within them during the process. These condensates exert two major effects: (1) they promote mitochondrial dysfunction by releasing or presenting αSyn species that interact with mitochondrial membranes, leading to depolarization and subsequent mitophagy, and (2) they sequester lipid droplets, impairing their turnover and limiting fatty acid availability for mitochondrial β-oxidation. The magnitude of these effects is modulated by αSyn charge, with additional lysine residues enhancing condensate formation and functional impact. Importantly, both condensate formation and mitochondrial dysfunction are reversible upon reduction of lipid droplet abundance. Dashed arrows indicate proposed relationships that were not directly tested in this study.

There is growing but underexplored evidence implicating lipid droplets and aberrant LLPS in αSyn dysregulation. This interaction may be particularly relevant in the microenvironment of midbrain dopaminergic neurons in the substantia nigra, which are enriched in lipid droplet-containing neuromelanin (Filimontseva et al, 2025). Using a neuronal cell model, we show that αSyn undergoes aberrant phase separation upon association with lipid droplets, forming biomolecular condensates that impair cellular energy homeostasis by disrupting both lipid droplet turnover and mitochondrial function. Notably, these effects are modulated by αSyn charge, with variants containing additional positively charged lysine residues exhibiting more pronounced dysfunction (Fig. 10).

Our data indicate a novel pathway for the formation of early αSyn inclusions. The positively charged KTKEGV repeat motifs of αSyn promote its interaction with lipid droplets, which are surrounded by a monolayer of negatively charged phospholipids (Eubanks et al, 2025; Fanning et al, 2020). This interaction drives aberrant LLPS and the rapid formation of condensates within hours. Importantly, these condensates are largely reversible upon reduction of lipid droplet abundance, suggesting that they represent an early and potentially targetable stage of αSyn dysregulation.

At first glance, the observation that lipid droplets both promote αSyn phase separation and are subsequently incorporated into αSyn condensates may appear paradoxical. However, we propose a bidirectional model in which lipid droplets initially act as nucleation platforms that locally concentrate αSyn and promote phase separation. Once condensates are formed, they in turn sequester the lipid droplets through sustained protein-lipid interactions. This creates a self-reinforcing cycle whereby lipid droplets facilitate condensate formation, and condensates progressively trap lipid droplets, ultimately impairing their turnover.

Interestingly, lipid droplet-rich 3K αSyn condensates exhibit reduced fluorescence recovery compared to those formed in the absence of lipid-rich enrichment. While this could in part reflect increased density due to the incorporation of lipids, we favor a model in which lipid droplets actively modulate condensate material properties. Lipid interfaces provide a scaffold that enhances multivalent αSyn-lipid and αSyn-αSyn interactions, thereby increasing viscosity and promoting a transition toward a more gel-like state. Thus, the observed reduction in liquidity likely reflects an interaction-driven change in material state.

Mechanistically, our data supports a model in which αSyn condensates directly perturb mitochondrial function. Their dynamic, liquid-like properties enable the generation or release of αSyn species that associate with mitochondrial membranes, leading to localized membrane damage and depolarization. Mitophagy is subsequently activated as a secondary response to remove damaged mitochondria. This interpretation is consistent with prior studies showing that lipid-rich environments can stabilize αSyn condensates in intermediate material states that act as reservoirs for oligomeric species (Hardenberg et al, 2021; Kumar et al, 2018), as well as extensive evidence linking αSyn oligomers to mitochondrial dysfunction. For example, αSyn can associate with cardiolipin-containing membranes, impair complex I activity, and promote reactive oxygen species production (Choi et al, 2022), while oxidation of mitochondrial proteins such as the ATP synthase β-subunit contributes to permeability transition and neurotoxicity (Ludtmann et al, 2018). Notably, mitochondrial dysfunction has also been observed during Lewy body formation (Mahul-Mellier et al, 2020), and similar inclusion-mitochondria interactions have been described in other neurodegenerative diseases, including Huntington’s disease (Bauerlein et al, 2017), suggesting a potentially generalizable mechanism. Importantly, the mitochondrial defects observed here are reversible at the condensate stage, as lipid droplet depletion restores mitochondrial membrane potential.

In parallel, αSyn condensates impair cellular energy metabolism by sequestering lipid droplets and limiting their turnover. Lipid droplets normally serve as reservoirs of fatty acids that can be mobilized through lipolysis or lipophagy and transferred to mitochondria for β-oxidation (Rambold et al, 2015). Because efficient fatty acid utilization requires close spatial coupling between lipid droplets and mitochondria, sequestration of lipid droplets within αSyn condensates is likely to disrupt this process and restrict energy supply. Our findings are consistent with impaired turnover of entrapped lipid droplets and support a model in which condensates interfere with lipid mobilization. Future studies could focus on a more granular assessment and quantification of this impairment through β-oxidation measurements.

The extent of these effects depends on αSyn charge. WT αSyn condensates show limited impact on mitochondrial membrane potential, although they still affect lipid droplet turnover. In contrast, E46K and 3K variants, which contain additional positively charged lysines, exhibit stronger interactions with lipid droplets and membranes and induce both localized mitochondrial depolarization and increased mitophagy. These observations suggest that electrostatic interactions are a key determinant of condensate behavior and toxicity.

Our study also establishes a novel cellular model for investigating early αSyn inclusion formation through lipid droplet-induced LLPS. Induction of lipid droplets via oleic acid treatment drives condensate formation by WT and E46K αSyn, consistent with multiple independent studies implicating aberrant phase separation in early αSyn assembly (Agarwal et al, 2024; Hardenberg et al, 2021; Piroska et al, 2023; Ray et al, 2020; Wang et al, 2024). These findings further support the relevance of the 3K model, which, despite incorporating artificial mutations, recapitulates key features of disease-associated inclusions, including ultrastructural similarities to Lewy bodies (Eubanks et al, 2025; Moors et al, 2021; Shahmoradian et al, 2019). Notably, we find that 3K condensates closely resemble those formed by the disease-associated E46K mutant in both behavior and functional consequences.

We observed two morphologically distinct types of αSyn condensates: lipid droplet-rich “Swiss cheese” condensates and more compact condensates lacking visible lipid inclusions. We propose that these represent different types of αSyn-lipid interaction. Swiss cheese condensates likely arise in lipid-rich environments where αSyn binds to and stabilizes lipid droplets, leading to their incorporation into the condensate. In contrast, compact condensates may form under conditions of lower lipid availability or altered interaction strength, where protein-protein interactions dominate. This interpretation is supported by our dose-response data, where increasing lipid droplet abundance selectively expands the Swiss cheese population without significantly affecting compact condensates.

The role of S129 phosphorylation in αSyn biology remains complex and context-dependent. While phosphorylation at S129 has been reported to reduce fibrillization kinetics in some systems, it is also strongly associated with pathological inclusions in vivo (Ghanem et al, 2022; Oueslati, 2016). In our model, lipid droplet-rich 3K αSyn condensates exhibit increased S129 phosphorylation. There are several explanations for this observation. It is possible that the lipid droplets act as a nidus to which αSyn binds and phase separates, which in turn facilitates aggregation and subsequent S129 phosphorylation. Alternatively, αSyn associated with lipid droplets may be more accessible to kinases, leading to preferential phosphorylation within these condensates (Ramalingam et al, 2023b). This increase in S129 phosphorylation may contribute to the observed shift toward a more viscous, less dynamic material state in lipid droplet-rich condensates. However, these assemblies remain in a semi-solid regime rather than progressing to fully solid aggregates (Hardenberg et al, 2021).

More broadly, our results highlight a potential role for lipid droplets in neurodegeneration. Lipid droplet accumulation has been observed under oxidative stress conditions and is linked to intercellular lipid transfer processes involving microglia and apolipoproteins (Liu et al, 2017; Liu et al, 2015; Moulton et al, 2021). Dysregulation of lipid droplet homeostasis is also implicated in hereditary spastic paraplegias (Kara et al, 2016). The enrichment of lipid droplets within neuromelanin-containing dopaminergic neurons in the substantia nigra may therefore create a microenvironment that promotes αSyn condensate formation and early vulnerability in PD. Given the known heterogeneity of Lewy bodies in both protein and lipid composition (Lashuel, 2020; Shahmoradian et al, 2019; Wakabayashi et al, 2007), it is plausible that lipid-rich αSyn condensates represent a distinct subclass of early inclusions that exhibit arrested maturation and sustained cellular dysfunction.

Recent work has also linked αSyn condensates to alterations in lipid metabolism, including changes in triglyceride homeostasis (Zhang et al, 2025). While conceptually related, our findings identify lipid droplets as an early driver of αSyn condensate formation and demonstrate that these condensates exert direct, localized effects on organelle function, including impaired lipid droplet turnover and mitochondrial depolarization. Thus, our work provides a cellular and organelle-level mechanistic framework that complements and extends these observations.

A limitation of our study is the use of a YFP tag on αSyn, which could in principle influence its biophysical behavior, including LLPS. This is particularly relevant given that αSyn is substantially smaller than YFP, and protein size and valency can modulate LLPS. However, multiple independent studies have demonstrated that αSyn undergoes phase separation across a range of labeling strategies, including GFP/YFP-tagged constructs as well as minimally labeled or untagged protein in vitro, in vivo, and in cultured cells (Agarwal et al, 2024; Hardenberg et al, 2021; Wang et al, 2016). These findings support that LLPS is an intrinsic property of αSyn rather than an artifact of fluorescent tagging. Importantly, in our system, condensate formation is strongly modulated by lipid droplet abundance and αSyn mutation/charge state, arguing against a dominant contribution from the fluorescent tag. While we cannot fully exclude effects of the YFP tag on condensate properties, the consistency of our observations with prior studies and the internal modulation of the phenotype support the robustness of our conclusions.

We note that the present study is conducted in a cell line-based system, which enables controlled interrogation of αSyn condensate formation and material properties. While this model provides experimental tractability, it does not fully recapitulate the cellular complexity of primary neurons or human brain tissue. In particular, neuronal-specific factors such as lipid composition, and synaptic activity may influence αSyn behavior. Validation in iPSC-derived dopaminergic neurons or primary neuronal systems will therefore be important to assess the generalizability of these findings. However, the mechanistic insights described here, particularly the relationships between lipid droplets, condensate dynamics, and mitochondrial perturbations, provide a framework that can be tested in more physiologically relevant models in future studies.

## Methods

**Table:** Reagents and tools table

Reagent/resource	Source	Identifier
**Antibodies**		
Rabbit anti-α-synuclein (phosphoS129)	Abcam	Cat #:ab168381
Mouse anti-α-synuclein	BD Biosciences	Cat #:610786
IRDye 680RD goat anti-rabbit	LiCor	Cat #:925-68071
IRDye 800CW goat anti-mouse	LiCor	Cat #:925-32210
**Chemicals, enzymes and other reagents**		
Dulbecco’s Modified Eagle Medium (DMEM)	Thermofisher	Cat #:11965118
Fetal Bovine Serum (FBS)	Thermofisher	Cat #:16000044
Glutamax	Thermofisher	Cat #:35050061
Penicillin–Streptomycin	Thermofisher	Cat #:15140122
Trypsin	Thermofisher	Cat #:25300054
Dulbecco’s Modified Eagle Medium (DMEM) without phenol	Thermofisher	Cat #:31053028
Oleic Acid-Albumin from bovine serum	Sigma	Cat #:O3008-5mL
8 well coverglass bottom chamberslides	Ibidi	Cat #:80807
doxycycline hyclate	Sigma	Cat #:D9891-1G
Opti-MEM reduced serum medium (OMEM) without phenol	Thermofisher	Cat #:11058021
Lipofectamine RNAiMAX Transfection Reagent	Thermofisher	Cat #:13778150
Lipofectamine™ 3000 Transfection Reagent	Thermofisher	Cat #:L3000015
Hoechst 33342 trihydrochloride trihydrate nuclear dye	Thermofisher	Cat #:H3570
1,6-Hexanediol	Sigma	Cat #:240117-50 G
Sterile distilled water	Gibco	Cat #:15230162
HCS LipidTOX™ Deep Red Neutral Lipid Stain	Thermofisher	Cat #:H34477
Tetramethylrhodamine, Methyl Ester, Perchlorate (TMRM)	Thermofisher	Cat #:T668
Dulbecco’s phosphate-buffered saline (DPBS), calcium, magnesium	Gibco	Cat #:14040133
Dulbecco’s phosphate-buffered saline (DPBS), no calcium, no magnesium	Thermofisher	Cat #: 14190144
Phosphate-buffered saline (PBS)	Sigma	Cat #:P3813-10PAK
Linoleic Acid-Albumin from bovine serum albumin	Sigma	Cat #: L9530-5ML
RIPA Buffer (10X) #9806	Cell Signaling	Cat #: 9806S
Protease inhibitor cocktail	Sigma	Cat #: 11697498001
Halt™ Phosphatase Inhibitor Single-Use Cocktail	Thermofisher	Cat #: 78420
FastAP Thermosensitive Alkaline Phosphatase (1 U/μL)	Thermofisher	Cat #: EF0651
Pierce BCA Protein Assay Kit, 500 mL	Thermofisher	Cat #: 23227
NuPAGE LDS Sample Buffer, 4X (NP0007)	Thermofisher	Cat #: NP0007
NuPAGE Sample Reducing Agent, 10X (NP0009)	Thermofisher	Cat #: NP0009
NuPAGE™ 4 to 12%, Bis-Tris, 1.0–1.5 mm, Mini Protein Gels	Thermofisher	Cat #: NP0321BOX
Amersham Protran Premium 0.2 NC 150 mm×4 m 1 roll/PK	Cytiva	Cat #: 10600014
Odyssey Blocking Buffer	LiCor	Cat #: 927-60001
NuPAGE MES SDS Running Buffer, 20× (NP0002)	Thermofisher	Cat #: NP0002
PageRuler Plus Prestained Protein Ladder, 10 to 250 kDa (26619)	Thermofisher	Cat #: 26619
Intercept Antibody Diluent	LiCor	Cat #: 927-65001
Fisher BioReagents™ Microbiology Media: LB Agar, Miller (Powder)	Fisher Scientific	Cat #:BP1425-500
Fisher BioReagents™ Microbiology Media: LB Broth, Miller	Fisher Scientific	Cat #:BP1426-500
Ampicillin	Sigma	Cat #:A0166-5G
Kanamycin	Sigma	Cat #:K1377-1G
FastAP Thermosensitive Alkaline Phosphatase (1 U/μL)	Thermofisher	EF0651
BODIPY 558/568 C12	Thermofisher	Cat #: D3835
BODIPY 493/503	Thermofisher	Cat #: D3922
One Shot™ MAX Efficiency™ DH5α-T1R Competent Cells	Thermofisher	Cat #:12297016
Bafilomycin A1 (Baf-A1) V-ATPase inhibitor	Selleck Chemicals	Cat #: S1413
Lysotracker Deep Red	Thermofisher	Cat #: L12492
Lysotracker Blue DND-22	Thermofisher	Cat #: L7525
Mitotracker Red CMXRos	Thermofisher	Cat #: M7512
HiSpeed Plasmid Maxi Kit (25)	Qiagen	Cat #:12663
MycoAlert® PLUS Mycoplasma Detection Kit (30 Tests)	Lonza	Cat #:LT07-703/NC0529908
**Experimental models: cell lines**		
M17D 3K αSyn cell line, male origin	Drs. Ulf Dettmer and Tim Bartels (Dettmer et al, 2017)	N/A
M17D E46K (1K) αSyn cell line, male origin	Drs. Ulf Dettmer and Tim Bartels	N/A
M17D wild-type αSyn cell line, male origin	Drs. Ulf Dettmer and Tim Bartels	N/A
**Recombinant DNA**		
Mito-BFP construct	Addgene	Cat #: 49151
LAMP1-RFP construct	Addgene	Cat #: 1817
**Software**		
Python	https://www.python.org/downloads/	N/A
Fiji ImageJ 2.16.0	https://imagej.net/software/fiji/	N/A
Zen Lite	https://www.zeiss.com/microscopy/us/products/software/zeiss-zen-lite.html	N/A
GraphPad Prism version 10	https://www.graphpad.com/scientific-software/prism/	N/A

*Note:* The above list of reagents significantly overlaps the list recently published by us (Eubanks et al, 2025) due to the methodological overlap of the studies.

### Methods and protocols

#### Cell line generation

Synthetic human aS-WT::YFP and aS-E46K::YFP cDNA fragments were digested with BamHI and EcoRI and inserted into the corresponding restriction sites of the pLVX-TetOne-Puro lentiviral vector (Clontech/TaKaRa, Mountain View, CA). Silent mutations were introduced to BamHI and EcoRI recognition sites within the *SNCA* coding sequence. The recombinant plasmid was sequence-verified and subsequently used for lentiviral particle production, following previously described protocols (Dettmer et al, 2017). Early-passage human M17D neuroblastoma cells were transduced with aS-WT::YFP or aS-E46K::YFP lentiviral particles and selected for stable integration using puromycin. Cells expressing the YFP-tagged transgene were further enriched by fluorescence-activated cell sorting (FACS) after doxycycline induction. Resulting cell pools were expanded, tested again for expression upon doxycycline induction, and absence of mycoplasma was confirmed.

#### Tissue culture

*Cell maintenance*: M17D neuroblastoma cells stably overexpressing E35K + E46K + E61K mutant (3K) αSyn, E46K mutant (1K) or wild-type (WT) αSyn fused with Venus YFP on their C-terminus, kindly shared by Profs Ulf Dettmer and Tim Bartels were used in this study. Cells were maintained in Dulbecco’s Modified Eagle Medium (DMEM) (Thermofisher, Cat #:11965118) supplemented with 10% fetal bovine serum (FBS) (Thermofisher, Cat #: 16000044), 1% Penicillin–streptomycin (Thermofisher, Cat #:15140122) and 1% Glutamax (Thermofisher Cat #:35050061). Cells were kept at 37 °C, >90% humidity and 5% CO_2_. At 70–80% confluency, the cells were trypsinized (Thermofisher Cat #:25300054) and seeded in new flasks. Cells were periodically tested for mycoplasma contamination with the MycoAlert PLUS Mycoplasma Detection Kit (Lonza, Cat #:LT07-703). For imaging experiments, the cells were switched to DMEM without phenol red (Cat #:31053028) supplemented with FBS, glutamax and penicillin–streptomycin as described above, after plating.

*Cell plating*: Cells were seeded in eight-well chamber slides or six-well plates depending on experimental needs, at densities of 1.2 × 10^5^ cells/mL (3K) or 0.4 × 10^5^ cells/mL (WT, 1K). In total, 300 μL/well and 2 mL/well were seeded in the eight-well chamber slides and six-well plates, respectively.

*Doxycycline induction*: Twenty-four hours after seeding, cells were treated with 1 μg/mL doxycycline hyclate (#D9891-1G, Sigma) in DMEM without phenol red supplemented with FBS, penicillin/streptomycin and glutamax, to induce expression of αSyn-YFP. Forty-eight hours later, the media was switched to media without doxycycline. Experiments were conducted either on the same day or later.

*1,6-Hexanediol*: Stock 1,6-Hexanediol solution was prepared as we previously described (Eubanks et al, 2025). In all, 1.5% concentration that was incubated for 30 min was used in our experiments.

*Bafilomycin A1 treatment*: Cells were treated with 100 nM of Bafilomycin A1 (Baf-A1) V-ATPase inhibitor (#S1413, Selleck Chemicals) for 24 h. Lysosomal surface area was quantified after staining with 150 nM of Lysotracker Deep Red (#L12492, Thermofisher) for 3 h.

All experiments apart from the electron microscopy ones were repeated independently 5–6 times, as per standard practice.

#### αSyn inclusion quantification through tiling images

For each condition, we acquired 3 × 3 tiling images at ×20 for the YFP channel with 488 nm excitation. We segmented whole cells using Cellpose. Within each labeled cell, we detected inclusions using adaptive Otsu thresholding. For each cell, we recorded the count of inclusions. Results were exported to Excel tables.

#### αSyn inclusion quantification through ×63 images

3K αSyn cells were treated with doxycycline. Forty-eight hours later, they were treated with dose-response concentrations of Oleic acid-albumin (Sigma Cat #:O3008-5mL) and Linoleic acid-albumin (Sigma Cat #: L9530-5ML). The concentration of the stock solution was determined through the specific lot number of the bottle as per manufacturer instructions. An initial concentration of 1200 μM was prepared in DMEM without phenol red+FBS+penstrep+glutamax, which was then used for serial dilutions: 600, 300, 150, 75, 37.5, 18.75 μM. An untreated control (0 μM) was also used. Lipid droplets were visualized by staining with LipidTOX deep red (Thermofisher Cat #:H34477) at a concentration of 1:500 for 1 h.

We quantified the number and types (Swiss cheese, compact) of αSyn inclusions, MFI of αSyn inclusions, MFI and surface area of lipid droplets and the surface area of the cytoplasm through Python code. Micro SAM is a finetuned version of SAM (Segment Anything Model). Micro SAM extends SAM by fine-tuning for microscopy images to segment cells, organelles, and other objects typically found in biological images. We finetuned the pretrained vit-b-lm model on 442 annotated images to segment the αSyn inclusions to improve segmentation accuracy (Sacks et al, 2026). We used 48 images to evaluate the improvement in accuracy from the previous process which resulted in an improvement from 40% to 70% using the IoU metric. The code separates the image into its two separate channels, YFP and deep red. It starts by first preprocessing the YFP channel by applying a Gaussian blur to reduce noise, enhancing contrast using a sigmoid adjustment, and normalizing the image from 0 to 1. This image is then input into the pretrained machine learning model to segment the inclusions. Next, the code generates a labelled image of the cells from the YFP channel using cellpose. The lipid droplets are segmented into their own masks using an intensity threshold. The code then does a loop through each cell in the labelled cell mask and extracts the inclusions and lipid droplets in each cell. It then classifies each inclusion as either Swiss cheese or compact by checking if it has any overlap with a lipid droplet. Afterwards, it counts how many inclusions are in each cell, how many Swiss cheese or compact, and the lipid droplets in either the cytoplasm or inclusions. To measure the Mean Fluorescence Intensity (MFI), the code performs segmentation of inclusions, cells, and lipid droplets from the green and deep red channels. It loops through each cell in the labelled cell mask and extracts the inclusions and lipid droplets that overlap with each cell. Inclusions are then classified as either Swiss cheese or compact, and lipid droplets are categorized based on whether they are located inside or outside the inclusions. Using these segmented masks, the code retrieves the corresponding regions from the original image to access pixel intensity values. The MFI is calculated by summing the fluorescence signal within a mask and dividing by the number of pixels in that mask. This process is repeated to compute the MFI of lipid droplets and αSyn inclusions. Finally, the computed data for each cell is compiled and exported into an Excel spreadsheet.

#### Oleic acid pulse-chase experiment

WT, 1K and 3K cells were plated in eight-well coverglass bottom chamberslides. 24 h later they were treated with 1 μg/mL doxycycline hyclate for 48 h, followed by treatment with 600 μM of oleic acid for 24 h (“pulsed”). This concentration falls within the range commonly used in the literature to induce lipid droplets (Papadopoulos et al, 2015). At that point, one well for each cell line was stained with LipidTOX Deep Red at a concentration of 1:500 for 1 h and imaged. In half of the remaining wells, the media was replaced with media that did not contain oleic acid (“chased”). At 24 h intervals, one well for the pulsed condition and one well for the chased condition for each cell line was stained with LipidTOX deep red and imaged. This was done for a total of 4 days. On each day, FRAP measurements were also undertaken for the WT and 1K cells that were not chased in media without oleic acid. On the fourth day, one well that was pulsed in media not containing oleic acid and one that still contained oleic acid were treated with 40 nM of TMRM for 3 h and imaged through z-stacks to quantify mitochondrial membrane potential as described below.

For the quantifications in Fig. 1D–G, the binned analysis was done for the following ranges of MFI of the YFP channel:

**Table:** 

Experiment	YFP MFI range
1/12/26	1500–6000
1/19/26	1500–8000
1/27/26	2500–8500
02/03/26	2000–10,000
02/18/26	2000–9000

Data from the pulse-chase experiment is shown in Fig. 1D–G (inclusion and lipid droplet quantification in expression-matched cells), 2E-F (FRAP), 4C-H (inclusion quantification), EV 2C-E (lipid droplet quantification), 5C-F (TMRM).

#### Quantification of early αSyn inclusions

WT and 1K cells were plated in eight-well coverglass bottom chamberslides. Twenty-four hours later, they were treated with 1 μg/mL doxycycline hyclate for 48 h. Subsequently, they were treated with 600 μM of oleic acid. At the 2, 4, 6, 8 and 30 h timepoints post-oleic acid treatment, separate wells were treated with 1:500 of LipidTOX Deep Red plus 600 μM of oleic acid and imaged.

*Note*: All experiments in this manuscript were undertaken after 48 h of doxycycline induction unless otherwise stated. In addition, wherever cells were treated with OA, the concentration was 600 μM for 24 h, unless otherwise stated.

#### Mitophagy experiments

*Transfections*: The following constructs were used for transfections: mito-BFP was a gift from Gia Voeltz (Addgene plasmid # 49151; http://n2t.net/addgene:49151; RRID:Addgene_49151) (Friedman et al, 2011). LAMP1-RFP was a gift from Walther Mothes (Addgene plasmid # 1817; http://n2t.net/addgene:1817; RRID:Addgene_1817) (Sherer et al, 2003). Cells were transfected 48 h after doxycycline induction and imaged 2 days later. RNAiMAX (#13778150, Thermofisher) was used for the 3K M17D cells as we previously described (Eubanks et al, 2025). Lipofectamine 3000 (Cat #:L3000015) was used for the 1K and WT M17D cells because of reduced transfection efficiency with RNAiMax.

Mitophagy events were determined by the colocalization between mito-BFP and LAMP1-RFP puncta, as analyzed through Python code. First, it separates the image into its three separate channels (BFP, YFP, RFP). Then it preprocesses the green channel by applying a Gaussian blur to reduce noise, enhancing contrast using a sigmoid adjustment, and normalizing the image from 0 to 1. This image is then input into a pretrained machine learning model to segment the inclusions from the green images. Next, the code generates a labeled image of the cells from the YFP channel using cellpose. The orange and blue channels’ desired objects are then segmented using an intensity threshold. The orange thresholding mask is then filtered to exclude signal overlapping with the YFP channel, which represents either bleedthrough or true signal due to incorporation of lysosomal fragments in αSyn inclusions (Dettmer et al, 2017). The YFP mask is then dilated by a radius of 25 pixels. This mask is then cut down by removing the original inclusion objects, resulting in a “donut” mask surrounding the inclusions. The code then loops through each cell within the labelled cell mask. It extracts the YFP+ inclusions, RFP+ lysosomes, and BFP+ mitochondria within each cell, creating a separate mask for each of those. It also obtains the “donut” mask within each cell and extracts the BFP+ and RFP+ objects specifically within the donut mask region and outside the dilated inclusion region. Finally, it counts the mitophagy events in the donut region and outside the dilated area by counting how many RFP+ objects touch or overlap with a BFP+ object. This data is then exported as an Excel datasheet.

As an alternative method for quantifying mitophagy, cells were treated with 150 nM of Lysotracker Blue DND-22 (#L7525, Thermofisher) and 50 nM of Mitotracker Red CMXRos (#M7512, Thermofisher) for 3 h. Data analyses were completed as described above.

#### Pulse chase assay for lipid droplet turnover

This assay was undertaken as previously described (Rambold et al, 2015). Cells were stained overnight with 1 µM of BODIPY 558/568 C12 (Thermofisher, Cat #: D3835), which is a fluorescent precursor to various phospholipids. They were then chased in DMEM without phenol red + FBS + glutamax + penstrep for 24 h, which was supplemented with 600 μM of oleic acid for the 1K and WT cells. This was followed by staining with LipidTOX deep red (Thermofisher Cat #: H34477) 1:500 for 1 h and then by imaging. BODIPY 558/568 C12 was excited with the 561 nm laser, YFP with 488 nm and LipidTOX deep red with 640 nm.

Data was analysed through Python code. First, it separates the image into its three separate channels (LipidTOX deep red, C12, and YFP). It starts off by first preprocessing the YFP channel by applying a Gaussian blur to reduce noise, enhancing contrast using a sigmoid adjustment, and normalizing the image from 0 to 1. This image is then input into a pretrained machine learning model to segment the inclusions from the YFP images (Sacks et al, 2026). Next, the code generates a labelled image of the cells from the YFP channel using Cellpose. The deep red and C12 objects are segmented into their own masks using an intensity threshold. Then a separate mask is created for each of the deep red and C12 channels. If an object in the deep red mask is not overlapping or touching with a C12 mask, then it is added to the deep red-only mask, which represents the lipid droplets that do not exhibit C12 staining. The code then loops through each cell within the labelled cell mask. Within each cell, it extracts the inclusions. Then, for each individual cell, the code analyses the spatial relationship between inclusions and lipid droplets across different masks. For each category (total, deep red-only), the script determines how many lipid droplets are located within inclusions versus outside.

#### Fluorescence recovery after photobleaching (FRAP)

FRAP experiments were undertaken using either a Zeiss LSM880 confocal with AiryScan with a ×63 oil-immersion objective or a Zeiss LSM900 confocal with a ×63 oil-immersion objective, as we previously described (Eubanks et al, 2025). Cells were imaged 48 h after doxycycline induction. For the comparison between linoleic acid and oleic acid-treated 3K cells, treatment with 600 μM of each lipid for 16 h was used. Laser settings (power and number of frames) were optimized to ensure less than 10% background bleaching during the timecourse experiment. A region of interest (ROI) was drawn around the αSyn inclusion of interest. For 3K αSyn inclusions, 5 images were taken pre-bleach, followed by bleaching at 100% transmission with the 488 nm laser for 8 iterations and 35 post-bleaching cycles. For 1K and WT αSyn inclusions, 3 images were taken pre-bleach, followed by 12 images post-bleach. Of note, the total duration of the timecourse was the same in all these experiments. For analysis, ROIs were drawn for the cytoplasm within the same cell and the black background outside cells. All downstream steps, described in detail in our previous public publication (Eubanks et al, 2025) were undertaken through a Python script.

First, the data from the three ROIs are imported into the script through an Excel sheet. On top of this, the script requires some preliminary information from the user, such as the time of the bleaching and the timeframe. Every number in the columns is truncated to two decimal places. With the truncated data, it now computes several data points, such as the average numbers in each ROI before being bleached, and specifically for ROI2 (cytoplasm), it calculates the average of the numbers after being bleached. With these values, the script proceeds to calculate the normalized fluorescence intensity for ROI1. This is done by subtracting the corresponding ROI3 value (background) from the ROI1 value and dividing the result by the post-bleach average of ROI2. This normalized value represents the fluorescence recovery trend over time and is recorded in a new column. The script then fits these normalized values to an exponential recovery model. Once it fits, the code extracts the plateau value. Using the plateau and the average of the normalized values before bleaching, it further calculates the mobile fraction and the immobile fraction. All of the data is exported as an Excel spreadsheet. After this, a smooth exponential recovery curve is generated based on the fitted parameters from the FRAP analysis. It creates 1000 evenly spaced time points from 0 to the final recorded time, and uses the fitted exponential model to compute the corresponding fluorescence intensity values for each time point. These values are stored in a new DataFrame along with their normalized percentage values. The DataFrame is then exported as an Excel. Finally, the script generates a graph for the raw data, plotting the numbers of all three ROIs over time. A second graph is produced for the normalized values of ROI1, showing both the actual data points and the fitted exponential recovery curve. Lastly, a third graph is created to display the fitted curve in terms of percentage recovery, with a dashed line marking 100% as a reference for complete fluorescence restoration. All three plots are formatted for clarity and saved together as a single .jpg image to provide a comprehensive visual summary of the FRAP analysis. Quality control for the analysis of each individual inclusion was undertaken manually.

#### Mitochondrial membrane potential measurement

Cells were incubated for 40 min with 1 μg/mL Hoechst 33342 (Thermofisher Cat #:H3570) and 100 nM tetramethylrhodamine methyl ester (TMRM) (Thermofisher Cat #:T668) at 48 h after doxycycline induction as we previously described (Eubanks et al, 2025). Z-stacks were acquired for the Hoechst (excitation 405 nm), YFP (excitation 488 nm) and TMRM (excitation 561 nm) channels.

For analysis, cells were segmented through Cellpose (cyto3 model) as follows. The YFP and nuclear channels were stacked, we ran the 2D model on each slice and then enabled cross-stitching to link masks with sufficient overlap between adjacent slices. To accommodate size variability, we swept the diameter parameter and returned the first non‑empty segmentation. Nuclei were segmented through Cellpose. A mitochondria binary mask was derived from the orange channel using Otsu thresholding. Inclusions were detected independently within each labeled cell and slice from the green channel using an adaptive threshold method. Then we filtered the inclusions to keep the ones with area 10–10,000 pixels, then we removed the ones with circularity index <= 0.1. For each cell we defined 4 ROIs: Cell, Inclusions, Inclusion surroundings (dilation radius = 7), and Cell without Inclusions and Surroundings. For each ROI per slice, we calculated the TMRM MFI inside the thresholded mitochondria binary mask in the ROI. Values were averaged across the *z* axis to yield per-cell results.

As an alternative method for measuring mitochondrial membrane potential, cells were treated with 50 nM of Mitotracker Red CMXRos (#M7512, Thermofisher) for 3 h. The remaining procedure and analysis were done as described above for TMRM.

Carbonyl cyanide m-chlorophenyl hydrazone (CCCP) 20 μM for 3 h was used as a positive control.

Note for data analyses through Python: cyto3 model was used for all analyses apart from linoleic acid and mitophagy analyses, which instead loaded cyto.

#### Micropipette aspiration and whole-cell patch-clamp (MAPAC)

Micropipette aspiration and whole-cell patch-clamp (MAPAC) experiments were carried out in M17D cells expressing fluorescently labeled αSyn 3K condensates with and without oleic acid. Micropipettes were pulled by a MP-1000 micropipette puller (RWD) to obtain cylindrical tips with inner diameters around 1 μm and were subsequently bent to an angle of ~45° using microforge (DMF1000, World Precision Instruments). Pipette tips were filled with intracellular solution containing 140 mM KCl, 10 mM HEPES, 1 mM MgCl_2_, 10 mM EGTA and 3 mM Mg-ATP (pH 7.2, 270–290 mOsm; Sigma-Aldrich).

The pipette was mounted on a headstage connected to an Axon 700B amplifier (Molecular Devices) and a custom pressure-recording system consisting of an Arduino UNO R3(ELEGOO) and a pressure sensor (FTVOGUE, B07N8SX347; 100 Pa resolution). Patch pipettes typically have a resistance of 25–80 megaohms. A small positive pressure (~ 3 kPa) was applied during pipette approaching the cell to prevent tip clogging. After the pipette tip was in contact with the cell membrane adjacent to the target condensate, negative pressure was applied to establish a gigaohm seal on the cell membrane. Whole-cell access was achieved in current-Clamp mode (with I = 0) by applying brief suction pulses greater than 10 kPa. We did not observe a stable resting from these cells. Therefore, the intracellular environment was likely perturbed during MAPAC measurements.

To probe condensate mechanical behavior in cells, controlled suction pressure was applied under whole-cell configuration to aspirate part of the target condensate into the pipette tip. This enabled in situ characterization of condensate material properties. Electrical signals were filtered at 2 kHz, digitized at 10 kHz, and recorded using Clampex 10.2 software (Molecular Devices).

#### Western blot

3K, 1K and WT cells were plated at 2.2 × 10^5^ cells/mL density in six-well plates. After doxycycline induction for 48 h, the cells were treated with 600 μM of oleic acid for 16 h. They were then trypsinized and pelleted. The pellet was lysated for 15 min on ice through RIPA buffer (Cell Signaling, Cat#: 9806S) containing protease (Sigma, Cat#: 11697498001) and phosphatase inhibitors (Thermofisher, Cat#: 78420). The latter was omitted in the sample that would be used for the Alkaline phosphatase treatment (positive control). The samples were centrifuged at 10,000× *g* at 4 °C for 15 min. The protein concentration of the supernatant was measured through BCA assay (Thermofisher, Cat#: 23227). The samples were mixed with LDS (Thermofisher, Cat#: NP0007) and reducing reagent (Thermofisher, Cat#: NP0009), ran on 4–12% Bis-Tris gels (Thermofisher, Cat#: NP0321BOX), transferred onto nitrocellulose membranes (Cytiva, Cat#: 10600014) overnight, blocked with Odyssey blocking buffer (LiCor, Cat#: 927-60001) and incubated with the primary antibody diluted in Intercept antibody diluent (LiCor Cat#: 927-65001) overnight. The following day, the membranes were washed with TBST four times and TBS one time, for 10 min each. Secondary antibodies were incubated for 1–2 h at room temperature. The membranes were washed and imaged on a LiCor Odyssey CLx. The following antibodies were used: Mouse anti-αSyn 1:2000 (BD Biosciences Cat#: 610786), Rabbit anti-α-synuclein (phosphoS129) 1:1000 (Abcam, Cat#: ab168381). IRDye 680RD goat anti-rabbit 1:2000 (LiCor, Cat#: 925-68071), IRDye 800CW goat anti-mouse 1:2000 (LiCor, Cat#: 925-32210). Images were analyzed in Fiji/ImageJ.

#### Experimental process and data analysis for correlative light and electron microscopy (CLEM) and cryo-ET experiments

*Cell lysis*: A T75 flask of 3K M17D cells stained with MitoTracker was pelleted. The pellet was suspended in 600 μl of a hypotonic lysis buffer composed of 20 mM Tris-Base, 10 mM NaCl and 3 mM MgCl2 that were dissolved in distilled water (final pH 7.4) and incubated on ice for 15 min. The suspension was passed through a 25 G needle 20 times and centrifuged at 3000 rpm at 4 °C for 5 min. The supernatant contained the cytosolic and the pellet the nuclear fraction. The nuclear fraction was resuspended in PBS, left to settle, and sample was collected right above the settled pellet for vitrification on EM grids.

*Vitrification of cell lysate*: Cell lysates were first mixed with 6 nm gold fiducials (Electron Microscopy Sciences) to facilitate tilt series alignment during data processing. An aliquot of 3.5 μL of sample was applied to freshly glow-discharged Quantifoil R2/2 100 Holey Carbon Au NH2 finder grids (Quantifoil Micro Tools GmbH) and then plunge frozen using a Leica EM GP plunger (Leica Microsystems) operating at 20 °C and 95% humidity. Front blotting was performed for 6 s prior to plunging the EM grid in liquid ethane.

*Correlative light and electron microscopy*: Cell lysate grids were loaded into a Leica DM6 FS microscope equipped with a ×50 objective and a DFC 365 FX camera. The microscope stage was kept at −195 °C with LN2 vapor. Fluorescence and corresponding bright field montage images of the grids were acquired using the Leica LASX software. Areas of the grid with YFP+ and MitoTracker signals were marked as targets. Pre-screened cell lysate grids were then loaded into a 300 kV Titan Krios microscope (Thermo Fisher Scientific) equipped with a post-column BioQuantum energy filter (slit width of 20 eV) and K3 direct electron detector (Gatan, Inc.). Areas of interest containing YFP+ and MitoTracker+ densities were identified by low-magnification overview maps (×740) and search maps (×3600) by correlating with fluorescence images. 2D projection images and tilt series of target areas were collected at ×26,000 magnification with a pixel size of 3.422 Å/pixel. Imaging settings used were spot size 5, 100 μm condenser aperture, 100 μm objective aperture, and approximately −8 μm defocus. Tilt series were collected continuously from −60° to 60° with 3° step increments with a total dose of ∼120–150 e/Å2 per tilt series.

*Cryo-ET data processing and visualization*: Tilt series alignment, reconstruction, and post-processing were done in EMAN2 (Tang et al, 2007). Membranes were automatically segmented using the TomoSegMemTV package (Martinez-Sanchez et al, 2014) and refined manually when necessary in UCSF Chimera (University of California, San Francisco) (Pettersen et al, 2004). αSyn aggregates were segmented manually. Ribosomes were either segmented manually on UCSF Chimera or by neural network segmentation using EMAN2 (Bell et al, 2018; Chen et al, 2017).

#### Statistical analysis

Statistical analyses were undertaken using GraphPad Prism version 10.3.0. Mean +/− standard deviation (SD) is shown on all plots. Statistical tests used include one sample *t* test, unpaired two-tailed *t* test, one-way ANOVA with post hoc Tukey’s correction for multiple testing, and one-way ANOVA with test for linear trend. Correction for multiple testing was undertaken as appropriate. It was assessed whether the data meet the assumptions of the test through Prism. After optimization, each experiment was repeated independently 5–6 times. The data was normalized within each experiment to a common control condition, followed by pooling the data and statistical analysis, unless otherwise stated in the figure legends. Only significant differences are indicated on the graphs. The statistical test, number of biological replicates and significance levels are indicated in the respective figure legends. The images shown in the figures of this paper are after contrast adjustment that was applied uniformly to the entire channel. Analysis of the images through Python code was done as described in the methods section. Data analyses were blinded to the experimental conditions.

## Supplementary information

Peer Review File

Movie EV1

Movie EV2

Expanded View Figures

## Data Availability

Python code used in data analysis in this manuscript has been posted publicly on GitHub: https://github.com/eleannakara/Kara-Lab/tree/main/analyses_of_alpha-synuclein_biomolecular_condensates and https://github.com/sunghoojung/segment-classify-pipeline. Tomograms of cell lysates from 3K cells have been deposited in the EMDB under accession codes EMD-76979, EMD-76980, EMD-76981, and EMD-76983, showing a mitochondrion with pronounced membrane deformation in direct contact with an extensive network of αSyn condensates, a deformed mitochondrion in close proximity to αSyn condensates, a mitochondrion with putative αSyn species associated with the outer membrane, exhibiting irregular, ragged morphology, and a mitochondrion located near αSyn condensates but without direct interaction, respectively. Microscopy images and western blots have been uploaded at BioImage Archive with accession number: S-BIAD3320. The source data of this paper are collected in the following database record: biostudies:S-SCDT-10_1038-S44319-026-00856-8.
