# Supplementary material for: Lipid droplets promote aberrant liquid-liquid phase separation of alpha-synuclein impairing energy homeostasis
Source: EMBO Rep. 2026 Jul 2;27(15):4299–331. doi: 10.1038/s44319-026-00856-8 (PMC13458734; doi:10.1038/s44319-026-00856-8)
Supplement: Supplementary file 1 [file 44319_2026_856_MOESM1_ESM.pdf]

EMBO reports

Lipid droplets promote aberrant liquid-liquid phase separation of alpha-synuclein impairing energy homeostasis

Jose Cevallos, Elena Eubanks, Sunghoo Jung, Yiming Huang, Elyse Guadagno, Nora Jaber, Yuzhou Xia, Jinying Wang, Huan Wang, Neeharika Suvvari, Aryan Doshi, Nitya Ravinutala, Alejandro Mosera, Timothy Hsu, Jiya Mody, Benjamin Sacks, Anvi Narayan, Breanna Smith, Maia Wang, Meghana Gottapu, Heba Alnakhala, Nagendran Ramalingam, Arati Tripathi, Tim Bartels, Ulf Dettmer, Zheng Shi, Wei Dai, and Eleanna Kara

Corresponding author(s): Eleanna Kara (ek851@rwjms.rutgers.edu)

Review Timeline:

Submission Date:	20th Nov 25
Editorial Decision:	5th Jan 26
Revision Received:	9th May 26
Editorial Decision:	11th Jun 26
Revision Received:	18th Jun 26
Accepted:	22nd Jun 26

Editor: Esther Schnapp

Transaction Report:

(Note: With the exception of the correction of typographical or spelling errors that could be a source of ambiguity, letters and reports are not edited. Depending on transfer agreements, referee reports obtained elsewhere may or may not be included in this compilation. Referee reports are anonymous unless the Referee chooses to sign their reports.)

1st Editorial Decision **5th Jan 2026**

Dear Eleanna,

Thank you for the submission of your manuscript to EMBO reports and for your proposed revision plan based on the 4 referee reports we shared. The full set of referee reports as well as referee cross-comments are also pasted below.

As we discussed, all referees together ask for a number of new experimentation and important controls, and all concerns will need to be addressed. Given that you think that you can address all concerns, I would like to invite you to revise your manuscript with the understanding that the referee concerns must be fully addressed and their suggestions taken on board. Please address all referee concerns in a complete point-by-point response.

Acceptance of the manuscript will depend on a positive outcome of a second round of review. It is EMBO reports policy to allow a single round of major revision only and acceptance or rejection of the manuscript will therefore depend on the completeness of your responses included in the next, final version of the manuscript.

We realize that it is difficult to revise to a specific deadline. In the interest of protecting the conceptual advance provided by the work, we recommend a revision within 3 months (7th Apr 2026). Please discuss the revision progress ahead of this time with me if you require more time to complete the revisions.

You can either publish the study as a short report or as a full article. For short reports, the revised manuscript should not exceed 29,000 characters (including spaces but excluding materials & methods and references) and 5 main plus 5 expanded view figures. The results and discussion sections must further be combined, which will help to shorten the manuscript text by eliminating some redundancy that is inevitable when discussing the same experiments twice. For a normal article there are no length limitations, but it should have more than 5 main figures and the results and discussion sections must be separate. In both cases, the entire materials and methods must be included in the main manuscript file.

IMPORTANT NOTE: we perform an initial quality control of all revised manuscripts before re-review. Your manuscript will FAIL this control and the handling will be DELAYED if the following APPLIES:
1) A data availability section providing access to data deposited in public databases is missing. If you have not deposited any data, please add a sentence to the data availability section that explains that.
2) Your manuscript contains statistics and error bars based on n=2. Please use scatter blots in these cases. No statistics should be calculated if n=2.

When submitting your revised manuscript, please review the submission guidelines (https://link.springer.com/journal/44319/submission-guidelines#cms-Revised-submissions) and carefully review the instructions that follow below. Failure to include requested items will delay the evaluation of your revision.

1) a .docx formatted version of the manuscript text (including legends for main figures, EV figures and tables). Please make sure that the changes are highlighted to be clearly visible.

2) individual production quality figure files as .eps, .tif, .jpg (one file per figure). See hhttps://media.springernature.com/original/springer-cms/rest/v1/content/27825798/data/v1 for more info on how to prepare your figures.

3) We replaced Supplementary Information with Expanded View (EV) Figures and Tables that are collapsible/expandable online. A maximum of 5 EV Figures can be typeset. EV Figures should be cited as 'Figure EV1, Figure EV2" etc... in the text and their respective legends should be included in the main text after the legends of regular figures.

- For the figures that you do NOT wish to display as Expanded View figures, they should be bundled together with their legends in a single PDF file called *Appendix*, which should start with a short Table of Content. Appendix figures should be referred to in the main text as: "Appendix Figure S1, Appendix Figure S2" etc. See 'Expanded View' section of the submission guidelines.

- Additional Tables/Datasets should be labeled and referred to as Table EV1, Dataset EV1, etc. Legends have to be provided in a separate tab in case of .xls files. Alternatively, the legend can be supplied as a separate text file (README) and zipped together with the Table/Dataset file.

4) a .docx formatted letter INCLUDING the reviewers' reports and your detailed point-by-point responses to their comments. As part of the EMBO Press transparent editorial process, the point-by-point response is part of the Review Process File (RPF), which will be published alongside your paper.

5) a complete author checklist, which you can download from our author guidelines. Please insert information in the checklist that is also reflected in the manuscript. The completed author checklist will also be part of the RPF.

6) Please note that all corresponding authors are required to supply an ORCID ID for their name upon submission of a revised manuscript (), which can be linked to your profile in the manuscript submission system.

7) Before submitting your revision, primary datasets produced in this study need to be deposited in an appropriate public database. Please remember to provide a reviewer password if the datasets are not yet public. The accession numbers and database should be listed in a formal "Data Availability" section placed after Methods. Please note that the Data Availability Section is restricted to new primary data that are part of this study. * Note - All links should resolve to a page where the data can be accessed. *

If your study has not produced novel datasets, please mention this fact in the Data Availability Section.

8) At EMBO Press we ask authors to provide source data for the main manuscript figures. You will receive a separate email with instructions for providing source data with your revised manuscript, including how to upload and organize the files.

9) Our journal also encourages inclusion of *data citations in the reference list* to directly cite datasets that were re-used and obtained from public databases. Data citations in the article text are distinct from normal bibliographical citations and should directly link to the database records from which the data can be accessed. In the main text, data citations are formatted as follows: "Data ref: Smith et al, 2001" or "Data ref: NCBI Sequence Read Archive PRJNA342805, 2017". In the Reference list, data citations must be labeled with "[DATASET]". A data reference must provide the database name, accession number/identifiers and a resolvable link to the landing page from which the data can be accessed at the end of the reference.

10) Regarding data quantification, the following points must be specified in each figure legend:

- the name of the statistical test used to generate error bars and P values,

- the number (n) of independent experiments (please specify technical or biological replicates) underlying each data point,

- the nature of the bars and error bars (s.d., s.e.m.),

- If the data are obtained from n Program fragment delivered error ``Can't locate object method "less" via package "than" (perhaps you forgot to load "than"?) at //ejpvfs23/sites23b/embor_www/letters/embor_decision_revise_and_review_sr_any.txt line 55.' 2, use scatter blots showing the individual data points.

Discussion of statistical methodology can be reported in the materials and methods section, but figure legends should contain a basic description of n, P and the test applied.

- Please also include scale bars in all microscopy images.

11) The journal requires a statement specifying whether or not authors have competing interests (defined as all potential or actual interests that could be perceived to influence the presentation or interpretation of an article). In case of competing interests, this must be specified in your disclosure statement.

12) All Materials and Methods need to be described in the main text using our 'Structured Methods' format, which is required for all research articles. According to this format, the Methods section includes a separate Reagents and Tools Table file (listing key reagents, experimental models, software and relevant equipment and including their sources and relevant identifiers) and a Methods and Protocols section describing the methods using a step-by-step protocol format. The aim is to facilitate adoption of the methodologies across labs. More information on how to adhere to this format as well as a downloadable template (.docx) for the Reagents and Tools Table can be found in our author guidelines.

We would also welcome the submission of cover suggestions, or motifs to be used by our Graphics Illustrator in designing a cover.

As part of the EMBO publication's Transparent Editorial Process, EMBO reports publishes online a Review Process File (RPF) to accompany accepted manuscripts. This File will be published in conjunction with your paper and will include the referee reports, your point-by-point response and all pertinent correspondence relating to the manuscript.

You are able to opt out of this by letting the editorial office know (emboreports@embo.org). If you do opt out, the Review Process File link will point to the following statement: "No Review Process File is available with this article, as the authors have chosen not to make the review process public in this case."

I look forward to seeing a revised form of your manuscript when it is ready.

Best regards,

Esther

Esther Schnapp, PhD
Senior Editor
EMBO reports

Referee #1:

The authors propose that α-synuclein undergoes LLPS on lipid droplets, impairing lipid droplet turnover and inducing mitochondrial dysfunction leading to mitophagy. However, their results and interpretations are too preliminary to be scientifically meaningful, owing to immature experimental design and insufficient supporting data. Most critically, the 3K, 1K, and WT cell lines show markedly different α-synuclein expression levels after doxycycline induction, confounding all mutation-dependent interpretations. Key controls are missing, and the evidence for mitochondrial dysfunction and mitophagy remains insufficient. More importantly, the lipid droplet-induced phase transition of α-synuclein into condensates is based on only very small differences in liquidity and phosphorylation, calling into question the central premise of the study-namely, the role of α-synuclein condensates.
Taken together, these issues indicate that the manuscript does not meet the standards required for a scientific publication. I therefore recommend rejecting the manuscript.

Comments to the Authors EMBOR-2025-63242V1
The manuscript investigates the role of lipid droplets in α-synuclein LLPS and proposes that such condensates impair lipid droplet turnover and induce mitochondrial dysfunction leading to mitophagy. While the study addresses an interesting hypothesis, several major concerns prevent the current version from supporting the authors' conclusions. These concerns are fundamental and would require extensive new experiments.
Major Concerns
1. Confounding differences in α-synuclein expression across cell lines
The Western blot in Fig. 2K shows that doxycycline-induced α-synuclein expression differs substantially among the 3K, 1K, and WT lines. It is unclear whether this reflects differences in protein stability caused by the mutations or inconsistencies in the generation of the cell lines. Quantification of α-synuclein mRNA levels would help clarify this.
More importantly, comparisons intended to reveal mutation-dependent phenotypes must use cell lines with matched expression levels. As presented, several key conclusions-such as the markedly increased inclusion numbers in 3K cells (Fig. 1B)-could simply result from higher α-synuclein expression rather than intrinsic properties of the mutation. This issue also affects the interpretation of S129 phosphorylation. These concerns undermine multiple central claims.
2. Lack of information on lipid droplet induction
Fig. 1B reports α-synuclein inclusions, yet it remains unclear whether oleic acid induces comparable numbers of lipid droplets across the three cell lines. Without this information, the interpretation of LD-associated α-synuclein condensates is incomplete.
3. Insufficient evidence supporting impaired lipid droplet turnover
The authors conclude that lipid droplets coated with α-synuclein exhibit reduced turnover. However, no direct metabolic analyses (e.g., β-oxidation measurements) are provided. Functional evidence is required to substantiate the proposed defect in lipid metabolism.
4. Missing essential experimental controls
Several key controls are absent:
• Fig. 1A lacks an OA- condition.
• In Fig. 1C, OA- and OA+ samples should be compared using identical doxycycline induction times, since α-synuclein levels are strongly time-dependent.
These omissions make it difficult to assess the specificity of the observed effects.
5. Interpretation of mitochondrial membrane potential and mitophagy (Fig. 3)
The TMRM decrease near α-synuclein condensates appears modest, and Fig. 3D shows mitophagy signals even in α-synuclein-negative regions. This suggests that the effect is not spatially restricted to α-synuclein condensates. A more direct reporter such as mito-Keima would be necessary to support the key claim that mitophagy occurs preferentially around α-synuclein condensates.
6. Reduced liquidity of lipid droplet-associated αSyn condensates (Fig.1H)
The authors suggest that lipid droplet-associated α-synuclein condensates show reduced liquidity, based on the observation that spontaneously formed 3K αSyn inclusions (without oleic acid) are more liquid than the lipid droplet-rich 3K inclusions formed after oleic acid treatment (Fig. 1H). However, the difference observed in Fig. 1H between OA- and OA+ conditions is relatively small. It remains unclear whether this minor difference reflects the early stage of a maturation process that might progress to a more solid state over longer periods. To rigorously support the claim of a solid-state transition, a biochemical assay such as sarkosyl fractionation or similar approaches would be valuable. Evaluating the time-dependence of condensate maturation could provide important mechanistic insight.
Overall Assessment
Due to these fundamental methodological and interpretational issues-particularly the confounding differences in α-synuclein expression levels-the main conclusions are not supported by the data. Resolving these concerns would require substantial re-design of core experiments, which goes beyond the scope of a revision. For these reasons, the manuscript is not suitable for

publication in EMBO Reports in its current form.

Referee #2:

In this manuscript, the authors provide compelling evidence for a mechanism linking lipid droplets, aberrant liquid-liquid phase separation (LLPS) of α-synuclein, and mitochondrial dysfunction in Parkinson's disease pathogenesis. This paper can reach the level of publication after a major revision.

Major concerns
The mechanism underlying mitochondrial toxicity remains unclear. The manuscript proposes two non-mutually exclusive models-release of toxic oligomers versus "off-target" mitophagy-but does not adequately distinguish between them. More direct mechanistic evidence is required, for example by testing whether inhibition of oligomer formation rescues mitochondrial depolarization. In addition, the choice of a 7-pixel radius appears somewhat arbitrary; a sensitivity analysis would strengthen the robustness of the conclusions. As an essential requirement for publication, the authors should more clearly differentiate between the proposed models of mitochondrial toxicity and provide experimental evidence supporting the dominant mechanism.

Minor concerns
1. Statistical considerations: Some experiments normalize data within each replicate before pooling-while this controls for batch effects, it may obscure absolute effect sizes. It would benefit from showing both normalized and raw data more consistently. Multiple comparison corrections are mentioned but not always clearly specified.
2. Temporal Dynamics: The time course of condensate formation → lipid droplet entrapment → mitochondrial dysfunction is unclear. Live-cell time-lapse imaging would help establish causality.
3. Reversibility: Can condensates dissociate if oleic acid is removed for longer periods? Is the mitochondrial dysfunction reversible? These questions impact whether this represents an early, potentially targetable stage.

Specific technical points
Figure 2: The dose-response experiments are excellent, but it would be helpful to know the physiological range of oleic acid concentrations in neurons.
Figure 3B: The mitochondrial depolarization around condensates is compelling, but TMRM in "redistribution mode" can be complex to interpret. Consider validating with alternative methods (e.g., Mito Tracker, JC-1).
Figure 4: The pulse-chase experiment is elegant, but the binary classification (C12+/− with LipidTOX+) may miss more subtle changes in lipid turnover rates.
Western blot (Fig 2J,K): The pS129 signal for WT and 1K appears very faint-are these truly negative or just below detection? Longer exposures or more sensitive detection might help.

Referee #3:

This study systematically investigates the role of lipid droplets in the aberrant liquid-liquid phase separation of α-synuclein and further reveals their impact on mitochondrial function and energy metabolism. Through cellular models, imaging techniques, and biochemical analysis, the authors propose that lipid droplets can serve as "nucleation sites" for abnormal αSyn aggregation, forming cytotoxic biomolecular condensates. This mechanism offers new insights into the early neuronal degeneration associated with Parkinson's disease (PD). The research is well-designed with comprehensive data and high-quality images, demonstrating strong innovation and mechanistic depth. However, certain experimental details, data interpretation, and logical coherence should be further refined.

1. All phase separation experiments in this study were conducted in cells, where the co-localization of lipid droplets with αSyn does not necessarily indicate that lipid droplets induce phase separation. It is also possible that αSyn aggregates "recruit" lipid droplets. To eliminate interference from the cellular environment, the authors should supplement their findings with relevant in vitro phase separation experiments.
2. In Fig. 1A, although the model is claimed to be more physiologically relevant, it lacks direct comparative validation with primary neurons or human brain tissues. It is recommended to supplement validation experiments in iPSC-differentiated dopaminergic neurons to enhance the generalizability of the results. Meanwhile，Fig. 1A needs to show a 3K confocal image without OA.
3. In Fig. 3, it is necessary to determine whether the same conclusion holds for 1K and WT α-Syn under OA treatment, especially since WT or E46K mutations are genuinely present in cells from both normal individuals and PD patients.
4. In Fig. 3A and B, why do α-Syn condensates cause depolarization of surrounding mitochondria? The underlying molecular mechanism requires further experimental investigation, and Fig 3D requires quantitative analysis.
5. In Fig. 3, the number of αsyn condensates is relatively low and they are clustered together, which differs from the condensates mentioned earlier in the text. Was this intentionally selected to investigate the impact of condensates on mitochondria? A description is required to explain this discrepancy.

6. The authors repeatedly describe αSyn condensates as "viscoelastic" or "gel-like" without providing any rheological measurements or mechanical characterization. These terms have precise physical meanings and should not be used based solely on FRAP immobile fractions or morphological appearance. Please remove or soften these physical descriptors (e.g., "partially dynamic" or "low mobility") unless relevant experimental data are provided to support viscoelastic or gel-like behavior.

Referee #4:

Manuscript number: EMBOR-2025-63242V1

This is an interesting manuscript exploring how αSyn liquid-liquid phase separation can be influence by lipid droplets. LLPS has become an important and emerging area of protein science, however, how condensate formation is triggered and the connection this has to neurodegeneration remain unclear. Furthermore, the contribution of lipid droplets towards phase separation processes is unknown. The authors explore this exciting topic with cell-based experiments. The experiments are robust and of high quality. My main concern is regarding whether the fluorescent protein is influencing the phase separation of αSyn. The authors are using αSyn-YFP. Given that αSyn is around half the size of YFP, I wonder what the effect of YFP is on the phase separation behaviour of αSyn. It is known that the size of biomolecules can strongly affect phase separation. I therefore recommend the authors perform experiments with another fluorescent system, to distinguish whether the YFP contributes to the phase separation of αSyn. This would really enhance the quality of the paper and help clarify things.

My other points are listed below:

1) In the Results section, the authors conduct FRAP experiments on the αSyn inclusions. They state that "Interestingly, 3K αSyn inclusions that formed spontaneously, without addition of oleic acid, were significantly more liquid than the more lipid droplet-rich 3K inclusions formed after treatment with oleic acid (fig.1F,G,H,I)." Could it be that the lipid rich inclusions are less liquid-like simply because they are denser, as there is more lipid present. The authors should elaborate on this and discuss whether they believe their observation is due to another factor.

2) How long after the αSyn inclusions were formed did the authors conduct the FRAP experiments? I suggest the authors conduct the FRAP experiments at different time points after the inclusions are formed. In this way, they can explore how the inclusions transition from liquid to solid.

3) Could the authors show magnified images of the Swiss cheese and compact condensates so that the reader can see the two different types. On that note, do the authors have any mechanistic insights into why one type is formed over the other? Some discussion on this would be helpful.

4) As the authors correctly mention, phosphorylation of αSyn is rather complicated and it is unclear how it contributes to pathology. In fact, it appears that phosphorylated αSyn aggregates slower than WT αSyn. Could the authors elaborate further on this and expand on the relevance of this in the context of their experiments. If phosphorylated αSyn is slower in aggregating, how are the data presented in this manuscript linked to pathology.

5) The authors state both that lipid droplets induce αSyn phase separation and that the αSyn condensates themselves entrap lipid droplets. This is a confusing mechanism. The authors should elaborate more on this point. This is quite important as it is a central aspect of their manuscript.

6) Start of the second paragraph of the Discussion; I suggest the authors rephrase this and don't make this sentence so absolute. More evidence needs to be accumulated before this statement can be confidently made. This comment also relates to Figure 5B-C, use of the word toxic may not be fully appropriate given the experiments so far. If the authors conducted further studies complementing their current mitochondrial experiments, then such terminology can be used.

Minor points:

7) At the end of the first paragraph of the Introduction, it should be noted that liquid-to-solid transitions of other neurodegenerative related proteins such as amyloid-beta have also been investigated (e.g. https://doi.org/10.1038/s41598-024-72265-7). This should be included.

8) In the second paragraph of the Introduction, the first sentence has no reference. A reference to back the claim should be added.

I recommend the authors revise their manuscript based on my comments above before the manuscript can be accepted for publication.

Cross-comments from referee 1:

Compared with the other two reviewers, our review may be somewhat stricter. However, after reading their reports in full, it is clear that our major concerns essentially align with those raised by Reviewers 2 and 3: the evidence for mitochondrial dysfunction and mitophagy is weak (Reviewer 2 - major comment; Reviewer 3 - comments 4 and 5), the causal relationship between oleic acid (lipid droplets) and α-synuclein condensates remains unclear (Reviewer 3 - comment 1), and the assessment of α-synuclein condensate properties, such as the FRAP assay, is insufficient (Reviewer 3 - comment 6). The major point of divergence is that Reviewers 2 and 3 did not address the differences in α-synuclein expression levels among the WT, 1K, and 3K cell lines. Since many of the main figures compare these mutant cell lines, we believe this issue cannot be overlooked.

Cross-comments from referee 2:

I agree with Reviewer #1's comments regarding mitophagy and concur that the authors need to provide additional evidence to support a direct link between LLPS and mitophagy. Overall, this is an interesting manuscript that is worthy of publication.

Cross-comments from referee 3:

In general, the concerns from Reviewer 1 are reasonable. However, I feel the story remains interesting and has novel aspects. If the authors can address these concerns by adding/replacing sufficient new experiments, I still recommend publication.

RESPONSE TO REVIEWERS

We thank the reviewers for their thoughtful comments. We have undertaken multiple additional experiments that have strengthened our observations and provided important mechanistic insight. As a result of those experiments, we have increased the total number of figures from 5 to 18 and included two new movies. We have also included 2 additional collaborators and 14 new co-authors that have undertaken part of those additional experiments and associated analyses: Nora Jaber, Yuzhou Xia, Jinying Wang, Huan Wang, Timothy Hsu, Jiya Mody, Benjamin Sacks, Anvi Narayan, Breanna Smith, Maia Wang, Meghana Gottapu, Heba Alnakhala, Zheng Shi, Wei Dai.

Changes in the text are highlighted in green.

Raw data and code are available at the following repositories:

- Code: https://github.com/eleannakara/Kara-Lab/tree/main/analyses_of_alpha-synuclein_biomolecular_condensates and https://github.com/sunghoojung/segment-classify-pipeline
- Tomograms: EMDB repository (EMDB: EMD-76979, EMD-76980, EMD-76981, and EMD-76983).
- Microscopy images and western blots, and associated quantifications: S-BIAD3320 (reviewer access link https://www.ebi.ac.uk/biostudies/bioimages/studies/S-BIAD3320?key=cd0c926e-7eb5-4af5-967e-842bbbb41713).

Referee 1:
The authors propose that α-synuclein undergoes LLPS on lipid droplets, impairing lipid droplet turnover and inducing mitochondrial dysfunction leading to mitophagy. However, their results and interpretations are too preliminary to be scientifically meaningful, owing to immature experimental design and insufficient supporting data. Most critically, the 3K, 1K, and WT cell lines show markedly different α-synuclein expression levels after doxycycline induction, confounding all mutation-dependent interpretations. Key controls are missing, and the evidence for mitochondrial dysfunction and mitophagy remains insufficient. More importantly, the lipid droplet-induced phase transition of α-synuclein into condensates is based on only very small differences in liquidity and phosphorylation, calling into question the central premise of the study-namely, the role of α-synuclein condensates.
Taken together, these issues indicate that the manuscript does not meet the standards required for a scientific publication. I therefore recommend rejecting the manuscript.

Comments to the Authors EMBOR-2025-63242V1
The manuscript investigates the role of lipid droplets in α-synuclein LLPS and proposes that such condensates impair lipid droplet turnover and induce mitochondrial dysfunction leading to mitophagy. While the study addresses an interesting hypothesis, several major concerns prevent the current version from supporting the authors' conclusions. These concerns are fundamental and would require extensive new experiments.
Major Concerns
1. Confounding differences in α-synuclein expression across cell lines
The Western blot in Fig. 2K shows that doxycycline-induced α-synuclein expression differs substantially among the 3K, 1K, and WT lines. It is unclear whether this reflects differences in protein stability caused by the mutations or inconsistencies in the generation of the cell lines. Quantification of α-synuclein mRNA levels would help clarify this.
More importantly, comparisons intended to reveal mutation-dependent phenotypes must use cell lines with matched expression levels. As presented, several key conclusions-such as the markedly increased inclusion numbers in 3K cells (Fig. 1B)-could simply result from higher α-synuclein expression rather than intrinsic properties of the mutation. This issue also affects the interpretation of S129 phosphorylation. These concerns undermine multiple central claims.

We agree with the reviewer that differences in αSyn expression levels across WT, 1K, and 3K cells could confound comparisons of inclusion frequency. To directly address this, we controlled for αSyn abundance at the single-cell level in all imaging-based analyses presented in Fig. 1. Specifically, for each independent experiment, we identified overlapping ranges of YFP fluorescence intensity (used as a proxy for αSyn levels) across the three cell lines and restricted the analysis to cells within these shared ranges. Thus, all comparisons of inclusion frequency were performed on cells with matched αSyn expression levels.

We note that *differences in bulk αSyn expression observed by western blot represent a limitation specifically for analyses that compare inclusion frequency across cell lines* (Fig. 1). In contrast, the remainder of the manuscript focuses on analyses at the level of individual condensates within cells, which are not dependent on comparisons of expression levels between lines.

Importantly, this concern does not affect our interpretation of S129 phosphorylation. In this study, pS129 was quantitatively analyzed within the 3K background (± OA), and therefore does not rely on comparisons across cell lines with differing expression levels. As noted in the revised manuscript, the pS129 signal detected in WT and 1K cells could be approaching the sensitivity limits of the assay, making it difficult to distinguish between absence of phosphorylation and levels below detection. This observation is consistent with prior reports indicating that S129 phosphorylation is enriched in the 3K variant (Imberdis *et al*, 2019; Ramalingam *et al*, 2023a) and in more aggregated or pathological αSyn states, as exemplified by the 3K variant in our system.

The following changes have been made to the manuscript:

The old plot comparing inclusion frequency between WT, 1K and 3K cells without matching expression levels has been replaced with the new panels below (figure 1D-F).

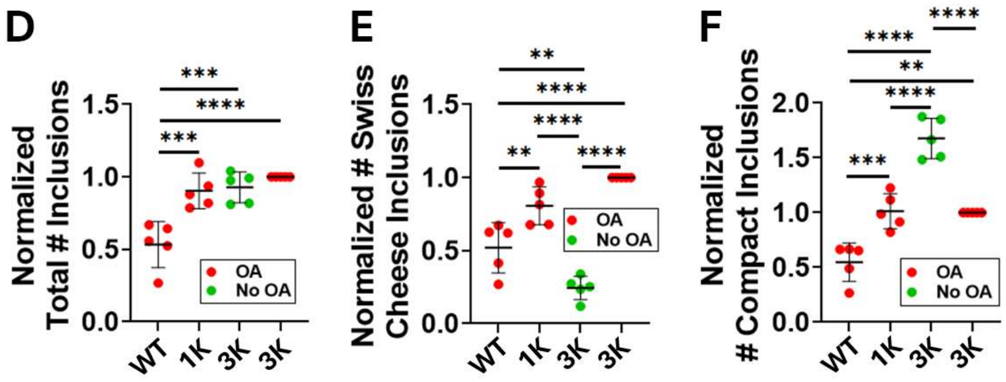

We have also added the following text to the manuscript:

"We next quantified inclusion formation across conditions (fig.1D,E,F). To ensure that the observed differences are due to the conditions being tested and not to unequal αSyn abundance between cells, we controlled for the differences in αSyn expression: we analyzed cells with matched protein levels, determined by the single-cell mean fluorescence intensity (MFI) of αSyn-YFP. Under these controlled conditions, 1K αSyn in the presence of oleic acid and 3K αSyn (with or without oleic acid) formed comparable numbers of inclusions per cell. In contrast, WT αSyn formed inclusions at approximately half this frequency (fig.1D).

We further distinguished between lipid droplet-rich ("Swiss cheese") and lipid droplet-poor ("compact") inclusions (fig.1E,F). In 3K-expressing cells, oleic acid treatment increased the frequency of Swiss cheese inclusions while reducing compact inclusions. WT αSyn formed significantly fewer inclusions of both types compared to 1K and 3K variants. Notably, under oleic acid treatment, 1K and 3K αSyn formed Swiss cheese inclusions at similar frequencies. However, oleic acid-treated 1K αSyn produced more Swiss cheese inclusions and fewer compact inclusions compared to untreated 3K αSyn (fig.1E,F). These findings suggest that the efficiency of inclusion formation is modulated by the increased positive charge of αSyn introduced by additional lysine residues."

"For the quantifications in figure 1D,E,F, the binned analysis was done for the following ranges of MFI of the YFP channel:

Experiment	YFP MFI range
1/12/26	1500-6000
1/19/26	1500-8000
1/27/26	2500-8500
02/03/26	2000-10000

**02/18/26**	**2000-9000**

“The pS129 αSyn signal detected in WT and 1K cells is probably approaching the sensitivity limits of the assay. While it is therefore difficult to definitively distinguish between absence of phosphorylation and levels below detection, this observation is consistent with prior reports indicating that S129 phosphorylation is enriched in the 3K variant (Imberdis *et al.*, 2019; Ramalingam *et al.*, 2023a) and in more aggregated or pathological αSyn states, which are exemplified by the 3K variant in this work.”

2. Lack of information on lipid droplet induction
Fig. 1B reports α-synuclein inclusions, yet it remains unclear whether oleic acid induces comparable numbers of lipid droplets across the three cell lines. Without this information, the interpretation of LD-associated α-synuclein condensates is incomplete.

We quantified the lipid droplets after oleic acid treatment and compared between cell lines. This experiment showed that lipid droplet formation was similar between cell lines but marginally more efficient in 3K cells.

The following changes have been made to the manuscript:

The new panel below (fig. 1G) has been added:

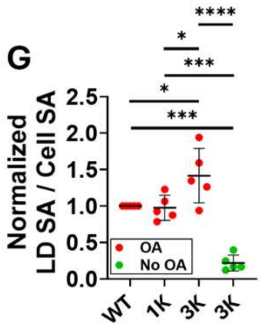

The following text has also been included:

“Of note, oleic acid treatment induced lipid droplet formation to a similar extent across WT, 1K, and 3K cell lines, and was marginally more efficient in 3K cells (fig.1G).”

3. Insufficient evidence supporting impaired lipid droplet turnover
The authors conclude that lipid droplets coated with α-synuclein exhibit reduced turnover. However, no direct metabolic analyses (e.g., β-oxidation measurements) are provided. Functional evidence is required to substantiate the proposed defect in lipid metabolism.

We thank the reviewer for this important point. While we did not directly measure β-oxidation, we addressed lipid droplet turnover using an expanded analysis of the C12 pulse-chase experiments. Please see our response to reviewer 2 (comment: “Figure 4: The pulse-chase experiment is elegant, but the binary classification (C12+/− with LipidTOX+) may miss more subtle changes in lipid turnover rates.”) where we address this matter.

Specifically, we moved beyond binary classification and quantified the amount of C12 turnover through its mean fluorescence intensity and integrated intensity. These results further confirm altered turnover patterns for lipid droplets entrapped in αSyn inclusions, as well as differences between the 3 cell lines.

We agree that direct assays of β-oxidation would provide complementary insight and have now acknowledged this as a limitation in the discussion. However, given that our study focuses on the interaction between αSyn condensates and lipid droplets, we prioritized imaging-based approaches that capture spatially resolved turnover dynamics at the organelle level.

The following changes have been made to the manuscript:

The following sentence has been added to the discussion:

“Future studies could focus on a more granular assessment and quantification of this impairment through β-oxidation measurements.”

4. Missing essential experimental controls
Several key controls are absent:
• Fig. 1A lacks an OA- condition.

We have included the 3K OA- condition as an additional column in figure 1A. The second column is new. All other columns remain unchanged.

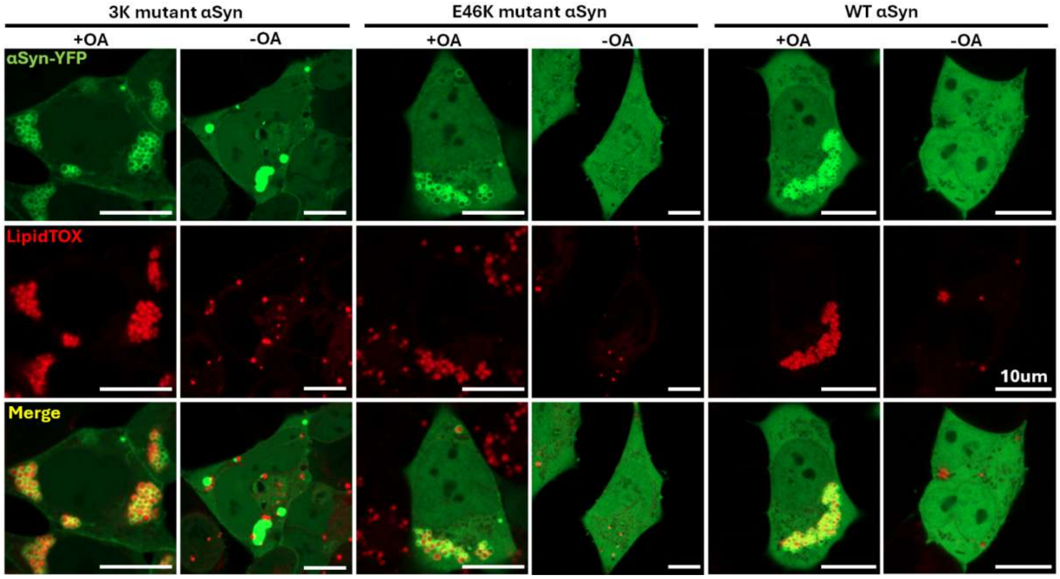

• In Fig. 1C, OA- and OA+ samples should be compared using identical doxycycline induction times, since α-synuclein levels are strongly time-dependent.

In the new figures 1D-G, all cell lines and conditions were induced with doxycycline for 48h. The only figure in which doxycycline induction times differed was figure 1F-J (formerly figure C-E). We have now clarified this in the legend of figure 1.

"D,E,F. The number of αSyn inclusions per cell were quantified in αSyn-YFP expression-matched cells after induction with doxycycline for 48h followed by treatment with 600uM of OA for 24h.

"All experiments in this figure and everywhere else in the manuscript were undertaken after 48h of doxycycline induction unless otherwise stated."

These omissions make it difficult to assess the specificity of the observed effects.

5. Interpretation of mitochondrial membrane potential and mitophagy (Fig. 3)
The TMRM decrease near α-synuclein condensates appears modest, and Fig. 3D shows mitophagy signals even in α-synuclein-negative regions. This suggests that the effect is not spatially restricted to α-synuclein condensates. A more direct reporter such as mito-Keima would be necessary to support the key claim that mitophagy occurs preferentially around α-synuclein condensates.

We agree that mitophagy is not exclusively spatially restricted to synuclein inclusions. We did not intend to imply spatial exclusivity, and indeed mitophagy events distal to inclusions are visible in Fig. 6 (which has replaced the old Fig. 3A). However, because the current quantifications are normalized, spatial heterogeneity is not readily apparent from the quantification plots.

To clarify this, we have added the following text to the manuscript:

"Mitophagy events were observed both in proximity to αSyn condensates and throughout the cytoplasm, indicating that mitophagy is not exclusively spatially restricted to inclusion-containing regions. However, quantitative analysis revealed a significant enrichment of mitophagy events in the vicinity of αSyn condensates compared to distal cytoplasmic regions."

To provide orthogonal validation of the LAMP1-RFP + mito-BFP colocalization experiments, we tested the mito-Keima reporter as suggested by the reviewer. However, in our system, the lower excitation channel of mito-Keima exhibited substantial bleedthrough from the YFP signal associated with αSyn inclusions, precluding reliable ratiometric measurements. As a result, mito-Keima was not compatible with our cell lines.

To address this, we performed an independent assay using Lysotracker Blue and Mitotracker Red to assess mitophagy. We quantified the colocalization of lysotracker and mitotracker in regions proximal to αSyn inclusions versus the remaining cytoplasm. This analysis revealed a significant enrichment of mitophagy-associated colocalization events in the vicinity of inclusions, thereby providing orthogonal support for our original findings.

To further validate the LAMP1-RFP and mito-BFP colocalization results, we performed a spatial sensitivity analysis by quantifying mitophagy events within progressively increasing radii surrounding αSyn condensates (1-30 pixels). This analysis revealed a sharp increase in mitophagy event frequency up to a radius of ~9 pixels, beyond which the signal reached a plateau, indicating that mitophagy is spatially enriched in the immediate vicinity of condensates.

We have made the following changes to the manuscript:

We have added a new supplementary figure 4 describing these findings.

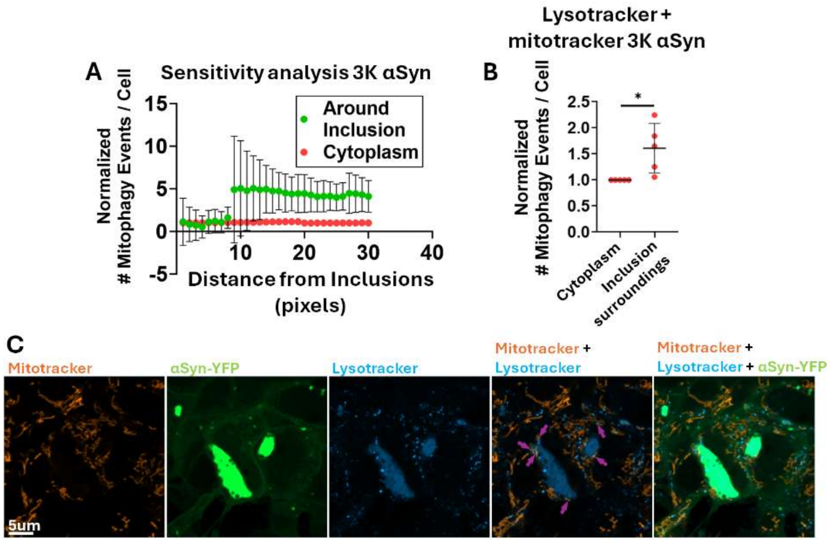

We have also added the text below to the manuscript:

"To validate our spatial analysis, we performed a sensitivity assessment of the radial cutoff. Quantification of mitophagy events around 3K αSyn condensates across radii ranging from 1 to 30 pixels revealed a plateau beginning at ~9 pixels, confirming that our 25-pixel radius provides a robust and inclusive measure of local effects (fig. EV2 A). As an independent validation, we used Lysotracker and Mitotracker staining and observed significantly increased colocalization around 3K αSyn condensates compared to the surrounding cytoplasm (fig. EV2 B,C), consistent with results obtained using the LAMP1-RFP/mito-BFP system."

6. Reduced liquidity of lipid droplet-associated αSyn condensates (Fig.1H)
The authors suggest that lipid droplet-associated α-synuclein condensates show reduced liquidity, based on the observation that spontaneously formed 3K αSyn inclusions (without oleic acid) are more liquid than the lipid droplet-rich 3K inclusions formed after oleic acid treatment (Fig. 1H). However, the difference observed in Fig. 1H between OA- and OA+ conditions is relatively small. It remains unclear whether this minor difference reflects the early stage of a maturation process that might progress to a more solid state over longer periods. To rigorously support the claim of a solid-state transition, a biochemical assay such as sarkosyl fractionation or similar approaches would be valuable. Evaluating the time-dependence of condensate maturation could provide important mechanistic insight.

We thank the reviewer for raising this point regarding the magnitude of the difference in liquidity observed in

Fig. 1H and the possibility of time-dependent maturation.

To directly and quantitatively assess the material properties of αSyn condensates, we employed micropipette aspiration and whole-cell patch-clamp (MAPAC), a sensitive approach for measuring condensate viscosity and elasticity developed by Dr. Zheng Shi (Wang et al, Science Advances, 2025 PMID 40249817). This method provides a more quantitative and mechanistic characterization of condensate material state than FRAP alone.

Using MAPAC, we found that 3K αSyn condensates formed under both OA- and OA+ conditions exhibit viscoelastic properties, with OA treatment consistently shifting condensates toward a more viscous and less fluid state. These results independently validate and extend our FRAP data, supporting the conclusion that lipid droplet-rich condensates adopt a less liquid material state. Thus, although the difference in immobile fraction measured by FRAP appears modest, it is robust and reproducible when assessed using an orthogonal and more sensitive biophysical technique.

To further address the question of time-dependent maturation, we performed FRAP time-course experiments over four days. As we previously reported for 3K αSyn (Eubanks et al., Cell Reports, 2025 PMID 40317721, fig. 2D), these condensates progressively increase in immobile fraction over time, consistent with maturation toward a more solid-like state. In the current study, we extended this analysis to WT and 1K αSyn and found that their immobile fractions remain largely stable, with a modest increasing trend for 1K. These data suggest a graded effect (WT < 1K < 3K) in which increasing positive charge correlates with progressive solidification.

Finally, we note that we focused on dynamic and quantitative measurements of condensate material properties (MAPAC and FRAP), as these are best suited to interrogate early, reversible αSyn assemblies. In contrast, detergent-insolubility assays such as sarkosyl fractionation are typically used to characterize end-stage, highly insoluble aggregates (e.g. Lewy bodies) and are less appropriate for capturing the properties of dynamic condensates formed over short timescales in our system.

We have made the following changes to the manuscript:

All FRAP and MAPAC data are now included in figure 2 instead of figure 1. The new panels are shown below:

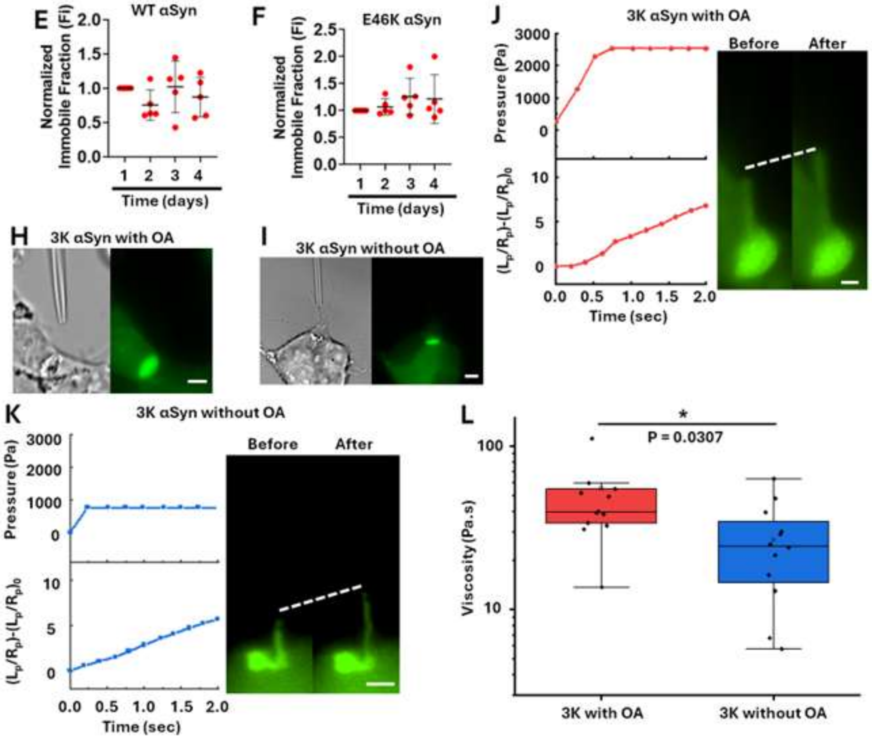

We have also added the following text to the manuscript:

"In order to characterize the maturation of αSyn condensates over time, we performed a 4-day timecourse experiment. FRAP measurements conducted every 24h showed that the immobile fractions

of WT and 1K αSyn condensates remain relatively stable, though the 1K variant shows a trend toward increased immobility over time (fig.2E,F). In our previous work, a similarly designed experiment for 3K αSyn showed a significant increase in Fi over 4 days (Eubanks *et al*, 2025).

To directly quantify the material properties of intracellular αSyn condensates, we performed micropipette aspiration and whole-cell patch clamp (MAPAC) measurements (Wang et al, 2025) on 3K αSyn condensates formed in M17D cells in the presence or absence of oleic acid. In this assay, a single condensate was gently drawn into the micropipette by stepwise aspiration pressure two to three times, and its deformation was quantified as the change in normalized aspiration length over time (fig.2H,I). Representative measurements showed progressive condensate entry into the pipette under applied pressure in both conditions, consistent with an apparent viscous response rather than purely elastic deformation (fig.2J,K).

Quantification of viscosity revealed that 3K αSyn condensates formed in the presence of oleic acid exhibited significantly higher viscosity than condensates formed without oleic acid ($p = 0.0307$) (fig.2L). Thus, oleic acid shifts 3K αSyn condensates toward a more viscous, less fluid material state. This result is consistent with the FRAP data showing that oleic acid treatment increases the immobile fraction of 3K αSyn condensates, indicating a transition toward a more solid-like or less dynamic condensate state. Together, these data support the conclusion that lipid-droplet-rich 3K αSyn condensates display increased resistance to flow under aspiration."

Overall Assessment
Due to these fundamental methodological and interpretational issues-particularly the confounding differences in α-synuclein expression levels-the main conclusions are not supported by the data. Resolving these concerns would require substantial re-design of core experiments, which goes beyond the scope of a revision. For these reasons, the manuscript is not suitable for publication in EMBO Reports in its current form.

Referee 2:
In this manuscript, the authors provide compelling evidence for a mechanism linking lipid droplets, aberrant liquid-liquid phase separation (LLPS) of α-synuclein, and mitochondrial dysfunction in Parkinson's disease pathogenesis. This paper can reach the level of publication after a major revision.

Major concerns
The mechanism underlying mitochondrial toxicity remains unclear. The manuscript proposes two non-mutually exclusive models-release of toxic oligomers versus "off-target" mitophagy-but does not adequately distinguish between them. More direct mechanistic evidence is required, for example by testing whether inhibition of oligomer formation rescues mitochondrial depolarization. In addition, the choice of a 7-pixel radius appears somewhat arbitrary; a sensitivity analysis would strengthen the robustness of the conclusions.

We agree that a sensitivity analysis across multiple radii would provide a systematic evaluation of this parameter. Rather than relying solely on radius-based colocalization metrics, we addressed this point using an orthogonal approach with higher spatial resolution. Specifically, we performed correlative light and electron microscopy (CLEM), which revealed αSyn oligomers coating the outer membrane of adjacent mitochondria, with clear evidence of membrane disruption at sites of contact.

These data provide direct ultrastructural evidence that the interaction between αSyn assemblies and mitochondria occurs at very close spatial proximity, supporting the validity of our original radius-based analysis and demonstrating that the observed mitochondrial repolarization around the condensates is not dependent on an arbitrary choice of radius.

We have made the following changes to the manuscript:

We included 4 new figures (fig. 8, fig. EV3 and fig. S4,5) and 2 new movies.

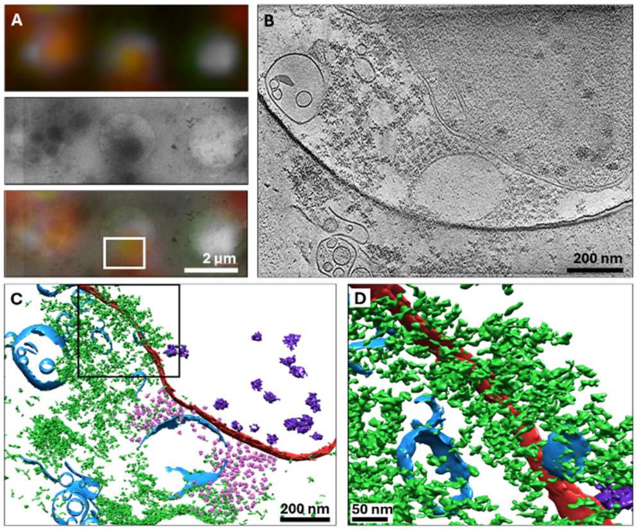

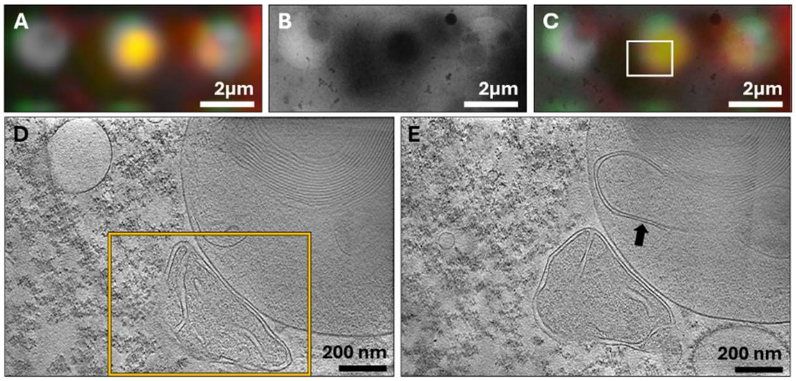

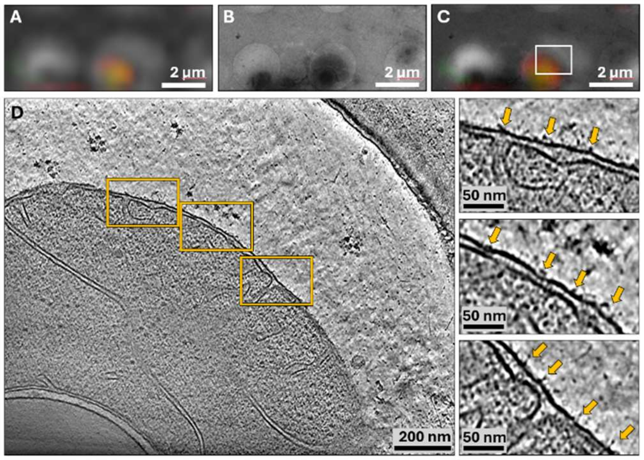

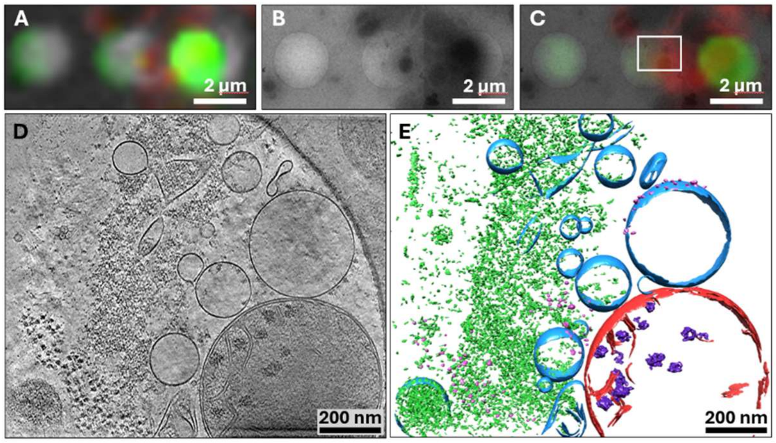

We also added the following to the text:

"To further probe this mechanism, we performed correlative light and electron microscopy (CLEM) and cryo-electron tomography (cryo-ET) on 3K αSyn samples as previously described (Eubanks et al., 2025). αSyn condensates were identified via their YFP signal, and mitochondria were labeled with Mitotracker for CLEM studies. Tilt series were collected from regions where the two signals were in close proximity or overlapped. In tomograms of cell lysate, αSyn appears as networks of amorphous structures, suggestive of higher-order assemblies of oligomers. These networks were often observed with ribosomes interspersed or entangled within them, suggesting potential direct interactions between the two. Mitochondria in direct contact with these large αSyn networks often exhibit clear morphological alterations, including irregular membrane contours and deviations from typical mitochondrial morphology (fig.8, fig.S4, movie EV1). Mitochondria were also observed enclosed within multivesicular body-like compartments. In some tomograms, mitochondria were observed with smaller, putative αSyn oligomeric species attached to the outer membrane. These attachments were often associated with pronounced membrane roughness, and in some cases, localized membrane disruption (fig. EV3). In contrast, mitochondria without direct interactions with αSyn condensates retained intact morphology (fig.S5, movie EV2)."

As an essential requirement for publication, the authors should more clearly differentiate between the proposed models of mitochondrial toxicity and provide experimental evidence supporting the dominant mechanism.

We thank the reviewer for this important point and have now provided supportive evidence for the dominant mechanism of mitochondrial toxicity. To address this, we performed two orthogonal experiments to determine whether mitophagy is a primary driver of toxicity or a secondary response.

First, we inhibited autophagic flux using Bafilomycin A1. If mitophagy were the primary event driving mitochondrial dysfunction, inhibition of autophagy would be expected to rescue both the increased mitophagy

events and the localized mitochondrial depolarization observed around αSyn condensates. Consistent with effective autophagy inhibition, Bafilomycin treatment rescued the spatial enrichment of mitophagy events (i.e. mitophagy levels around condensates were no longer elevated relative to the surrounding cytoplasm) and increased lysosomal surface area, confirming pathway inhibition. However, Bafilomycin treatment did not rescue the localized mitochondrial depolarization. These results indicate that mitophagy is not the primary driver of mitochondrial dysfunction, but rather a downstream response.

Second, we performed correlative light and electron microscopy (CLEM), which revealed oligomeric αSyn coating the outer mitochondrial membrane of mitochondria in close proximity to condensates, with clear evidence of membrane disruption at sites of contact.

Together, these data support a model in which αSyn condensates directly induce mitochondrial damage, leading to localized depolarization, followed by secondary activation of mitophagy to remove damaged mitochondria.

We have made the following changes to the manuscript:

We have included 6 new figures (fig.7,8, fig.S3,4,5, fig. EV3) and 2 new movies.

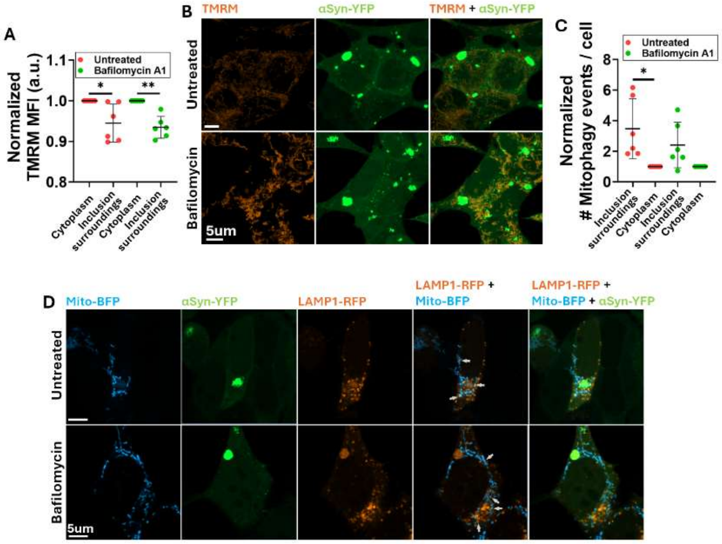

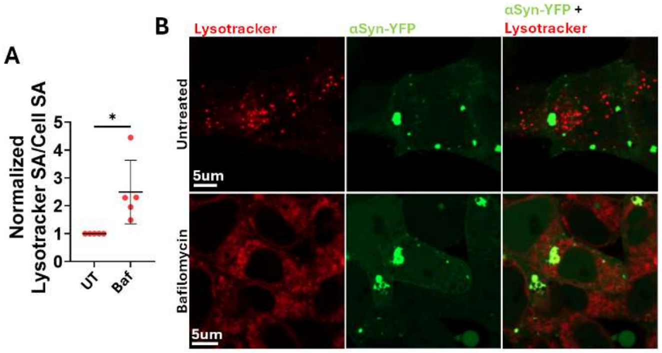

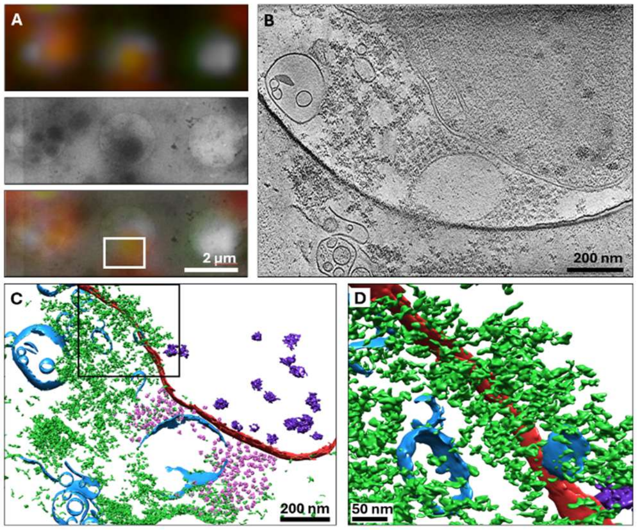

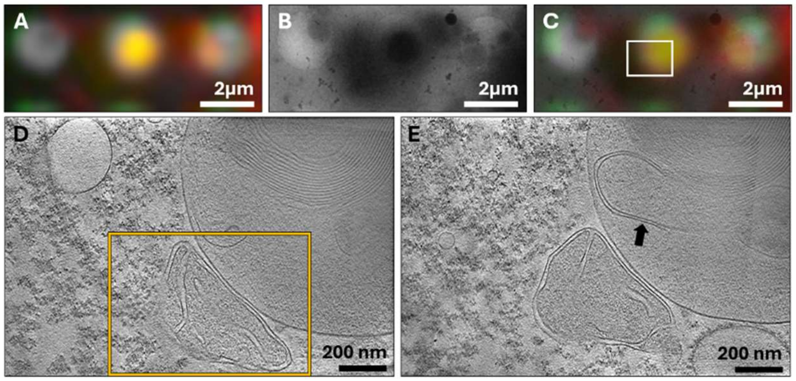

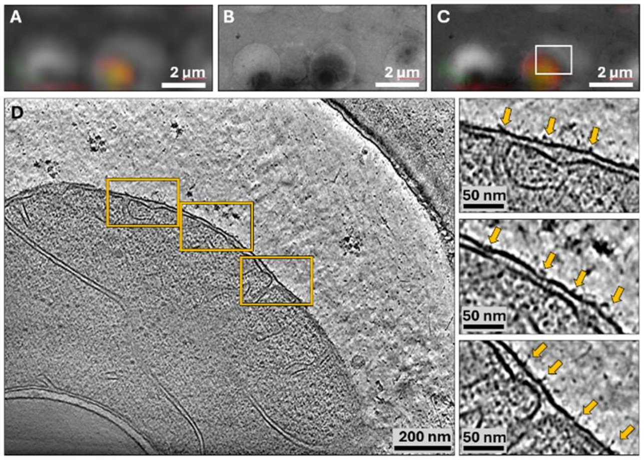

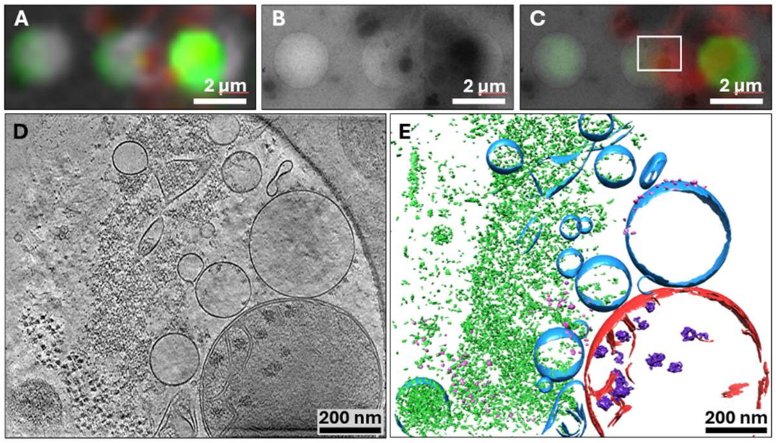

We also added the following to the text:

"<u>Mitochondrial damage precedes mitophagy</u>
Two mechanisms could explain the observed coupling between mitochondrial depolarization and increased mitophagy near αSyn condensates. In one scenario, αSyn condensates, by virtue of their dynamic, viscous nature, release toxic αSyn species that directly impair mitochondrial function, leading to depolarization and subsequent mitophagy. Alternatively, lysosomes may target αSyn condensates for degradation and, in doing so, nonspecifically engulf nearby mitochondria, resulting in secondary mitochondrial damage.

To distinguish between these possibilities, we treated 3K αSyn-expressing cells with Bafilomycin A1, an inhibitor of autophagic flux. Bafilomycin treatment led to a significant enlargement of lysosomal compartments, as quantified by Lysotracker staining, confirming effective pathway inhibition (fig.S3A,B). Under these conditions, mitochondria in proximity to αSyn condensates remained significantly depolarized (fig.7A,B). In contrast, the elevated mitophagy observed near condensates was abolished by Bafilomycin treatment (fig.7C,D).

These findings indicate that mitochondrial depolarization occurs independently of mitophagy and therefore precedes it. If lysosome-driven off-target degradation were the primary cause of mitochondrial damage, inhibition of autophagy would be expected to rescue both mitophagy and mitochondrial depolarization. Instead, the persistence of depolarization despite blocked mitophagy supports a model in which αSyn condensates directly induce mitochondrial damage, followed by secondary clearance through mitophagy.

To further probe this mechanism, we performed correlative light and electron microscopy (CLEM) and cryo-electron tomography (cryo-ET) on 3K αSyn samples as previously described (Eubanks et al., 2025). αSyn condensates were identified via their YFP signal, and mitochondria were labeled with Mitotracker for CLEM studies. Tilt series were collected from regions where the two signals were in close proximity or overlapped. In tomograms of cell lysate, αSyn appears as networks of amorphous structures, suggestive of higher-order assemblies of oligomers. These networks were often observed with ribosomes interspersed or entangled within them, suggesting potential direct interactions between the two. Mitochondria in direct contact with these large αSyn networks often exhibit clear morphological alterations, including irregular membrane contours and deviations from typical mitochondrial morphology (fig.8, fig.S4, movie EV1). Mitochondria were also observed enclosed within multivesicular body-like compartments. In some tomograms, mitochondria were observed with smaller, putative αSyn oligomeric species attached to the outer membrane. These attachments were often associated with pronounced membrane roughness, and in some cases, localized membrane disruption (fig. EV3). In contrast, mitochondria without direct interactions with αSyn condensates retained intact morphology (fig.S5, movie EV2).

Together, these data support a model in which αSyn condensates generate or concentrate toxic αSyn species that associate with mitochondrial membranes, causing structural damage and depolarization. This primary mitochondrial injury subsequently triggers mitophagy. Due to the higher abundance and brightness of condensates in 3K-expressing cells, CLEM analysis was feasible only in this condition."

Minor concerns

1. Statistical considerations: Some experiments normalize data within each replicate before pooling-while this controls for batch effects, it may obscure absolute effect sizes. It would benefit from showing both normalized and raw data more consistently. Multiple comparison corrections are mentioned but not always clearly specified.

We thank the reviewer for this important point. We agree that normalization within replicates, while useful for controlling batch effects, can obscure absolute effect sizes if presented alone. Therefore, we have included both normalized and unnormalized representations of the same dataset in Fig. 2B,C (formerly fig.1G,H).

Normalization is used to highlight relative differences in condensate dynamics across conditions, whereas the unnormalized data provide the absolute scale of the measurements and demonstrate that the average inclusion remains largely liquid. Presenting both views ensures that neither relative trends nor absolute magnitudes are obscured.

We have also revised the manuscript to more clearly specify, for each panel, the statistical tests used, significance thresholds, and corrections for multiple comparisons.

2. Temporal Dynamics: The time course of condensate formation → lipid droplet entrapment → mitochondrial dysfunction is unclear. Live-cell time-lapse imaging would help establish causality.

We agree that the temporal relationship between condensate formation, lipid droplet association, and mitochondrial dysfunction is an important question. However, we did not intend to imply a defined temporal sequence of events leading to condensate formation; rather, our central conclusion is that the presence of synuclein condensates, independently of the precise pathway by which they arise, is associated with impaired mitochondrial function.

The experiments that we undertook above (mitophagy inhibition, electron microscopy) helped resolve the mechanism with which condensates affect mitochondrial function.

3. Reversibility: Can condensates dissociate if oleic acid is removed for longer periods? Is the mitochondrial dysfunction reversible? These questions impact whether this represents an early, potentially targetable stage.

We agree that it is an important question to determine whether this phenomenon is reversible. To address this, we performed oleic acid (OA) pulse chase experiments in 3K, 1K, and WT cell lines. Cells were pulsed with OA for 24h to induce lipid droplet formation and condensate assembly, followed by replacement with OA-free medium and longitudinal imaging every 24h over 3 days to monitor condensate persistence or dissolution. In 1K and WT cells, the number of condensates progressively decreased over time, and this decrease was driven by the reduction in Swiss cheese condensates. A similar but less pronounced reduction was observed in the 3K cells.

At the end of the chase period, we assessed mitochondrial membrane potential using TMRM to determine whether OA withdrawal is associated with recovery of mitochondrial function. The cells that were chased in media without OA showed recovery of their mitochondrial membrane potential.

We have made the following changes to the manuscript:

A. **Pulse-chase experiment for quantification of condensates:**

We added 2 new figures (fig.4C-H, fig.S2C-E).

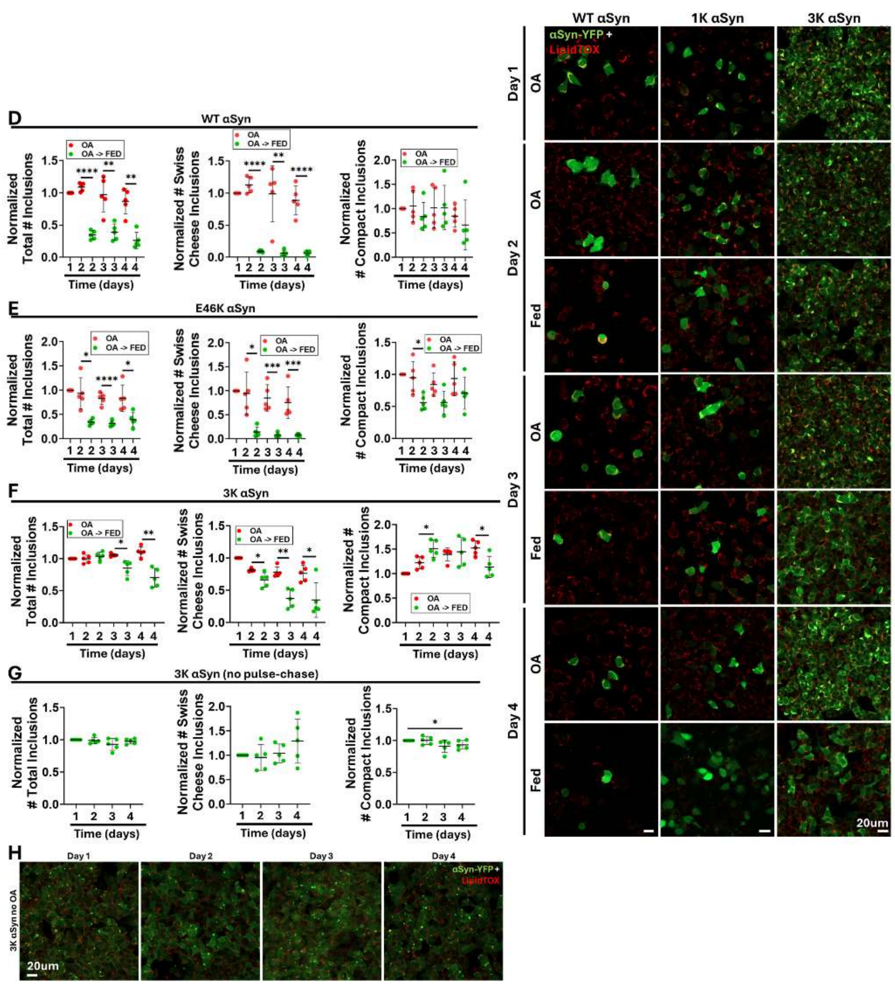

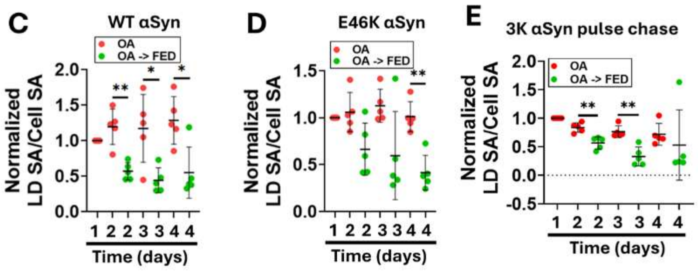

We also added the following to the text:

To test whether αSyn condensates are reversible, we performed a pulse-chase experiment. Cells were treated with oleic acid for 24h (pulse; day 1), followed by replacement with oleic acid-free media and imaging every 24h for an additional 3 days (chase; days 2-4). Parallel conditions included cells continuously maintained in oleic acid, as well as 3K αSyn-expressing cells without oleic acid treatment. Upon withdrawal of oleic acid, lipid droplet abundance progressively decreased over 4 days (fig.S2C,D,E).

Consistent with this reduction, the total number of αSyn condensates declined significantly over time in WT and 1K cells. This decrease was primarily driven by a reduction in Swiss cheese condensates, whereas compact condensates remained relatively stable (fig.4C,D,E). In 3K-expressing cells, the decrease in total condensates was more modest and delayed, becoming apparent mostly at days 3 and 4, and was similarly driven by loss of Swiss cheese condensates (fig.4C,F). In contrast, 3K αSyn in the absence of oleic acid remained largely stable over the 4-day period, with only a minor reduction in compact condensates observed at day 4 (fig.4G,H).

B. Pulse-chase experiments to measure mitochondrial membrane potential:

We added 1 new figure (fig.5C-F):

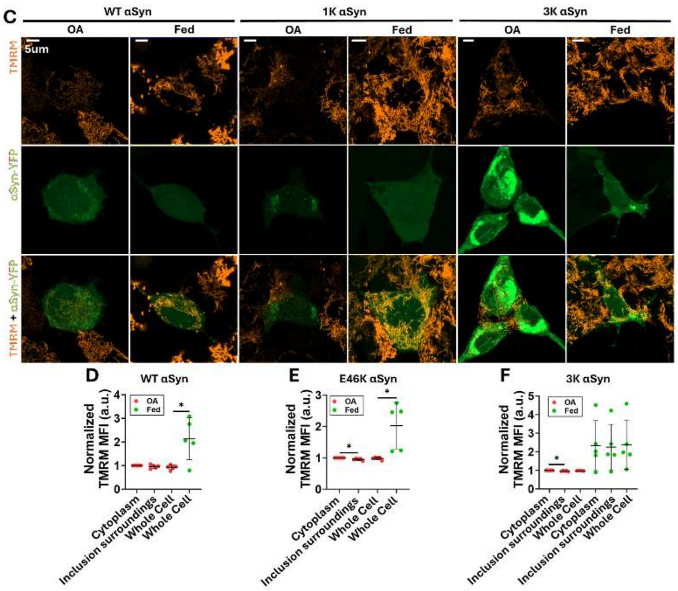

We also added the following to the text:

"We next extended this analysis to WT and 1K αSyn and compared them to 3K. Cells were induced with doxycycline for 48h, followed by oleic acid treatment for 24h, and Δψ was measured 96h (4 days) later. Parallel conditions included cells subjected to oleic acid withdrawal (pulse-chase; fig.4C-F). At the whole-cell level, withdrawal of oleic acid led to recovery of mitochondrial membrane potential across all three variants. In contrast, under continuous oleic acid treatment, mitochondria surrounding αSyn condensates in 1K and 3K cells were significantly depolarized compared to the surrounding cytoplasm. This localized depolarization was not observed in WT cells. Notably, in 3K cells subjected to oleic acid withdrawal, residual condensates persisted; however, mitochondria in their vicinity were no longer significantly depolarized (fig.5C-F)."

Specific technical points
Figure 2: The dose-response experiments are excellent, but it would be helpful to know the physiological range of oleic acid concentrations in neurons.

We agree that contextualizing the oleic acid (OA) concentrations used in our dose response experiments is important. In the current study, OA treatment is used as an established experimental approach to induce lipid droplet formation in cultured cells. It is not meant to precisely mimic free OA concentrations in neurons in vivo, which are difficult to define due to rapid metabolic buffering and compartmentalization (Kumar *et al*, 2025).

The OA concentrations used here fall within the range commonly employed in the literature to induce lipid droplets in neuronal and non-neuronal cell culture systems. We revised the methods section to explicitly state this rationale and to reference representative studies using comparable OA concentrations.

We added the following to the text:

WT, 1K and 3K cells were plated in 8-well coverglass bottom chamberslides. 24h later they were treated with 1ug/mL doxycycline hyclate for 48h, followed with treatment with 600uM of oleic acid for 24h ("pulsed"). This concentration falls within the range commonly used in the literature to induce lipid droplets (Papadopoulos *et al*, 2015).

Figure 3B: The mitochondrial depolarization around condensates is compelling, but TMRM in "redistribution mode" can be complex to interpret. Consider validating with alternative methods (e.g., Mito Tracker, JC-1).

We thank the reviewer for this important point. We agree that interpretation of TMRM in redistribution mode

can be complex and that orthogonal validation is essential.

To address this, we repeated the experiment in 3K cells using MitoTracker Red CMXRos, an independent probe of mitochondrial membrane potential (Alam et al, 2022). As expected, treatment with the mitochondrial uncoupler CCCP resulted in a significant reduction in MitoTracker signal, confirming assay sensitivity. Importantly, this analysis also revealed reduced mitochondrial membrane potential in mitochondria proximal to αSyn condensates, consistent with our observations using TMRM.

In addition, as a control for the TMRM assay, CCCP treatment similarly led to a marked decrease in TMRM signal, validating the reliability of the redistribution-based measurements.

Together, these results demonstrate that the observed mitochondrial depolarization is reproducible across independent probes and is not an artifact of TMRM redistribution.

We have made the following changes to the manuscript:

We added a new figure (fig. EV1):

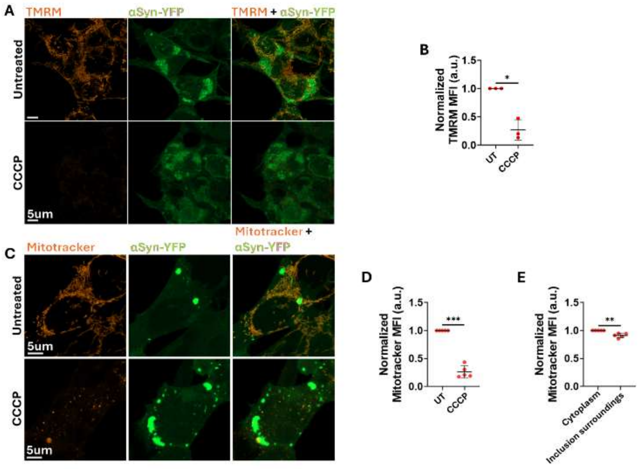

We also added the following to the text:

"To validate the robustness of our measurements, we used carbonyl cyanide m-chlorophenyl hydrazone (CCCP), a mitochondrial uncoupler, as a positive control. CCCP treatment led to a marked reduction in Δψ, confirming the sensitivity of the TMRM assay (fig. EV1 A,B). As a secondary validation of our membrane potential measurements, we employed an independent approach using MitoTracker CMXRos (Alam et al., 2022). This dye similarly demonstrated CCCP-induced depolarization and confirmed that mitochondria adjacent to 3K αSyn condensates exhibit reduced membrane potential (fig. EV1 C,D,E). Together, these controls establish the reliability of our mitochondrial measurements."

Figure 4: The pulse-chase experiment is elegant, but the binary classification (C12+/− with LipidTOX+) may miss more subtle changes in lipid turnover rates.

We thank the reviewer for this insightful comment. We agree that binary classification of C12 incorporation in LipidTOX-positive droplets may not fully capture more subtle differences in lipid turnover dynamics.

To address this, we expanded our analysis in the revised manuscript to include quantitative measurements of C12 signal within lipid droplets. Specifically, we measured the mean fluorescence intensity (MFI) of C12 within droplets located inside and outside αSyn condensates, and calculated integrated C12 signal (MFI × droplet area, as defined by LipidTOX staining) to account for both signal intensity and droplet size.

In our original binary analysis, we found that lipid droplets within condensates retain C12 labeling more frequently than droplets in the surrounding cytoplasm. The quantitative analysis further revealed that, while absolute C12 intensity measures vary across cell lines and conditions, lipid droplets associated with condensates exhibit distinct C12 signal profiles compared to cytoplasmic droplets.

Together, these results support the conclusion that lipid droplet turnover dynamics are altered inside αSyn

condensates, and demonstrate that this effect is detectable using both binary and continuous metrics.

We have made the following changes to the manuscript:

We added a new figure (fig.9):

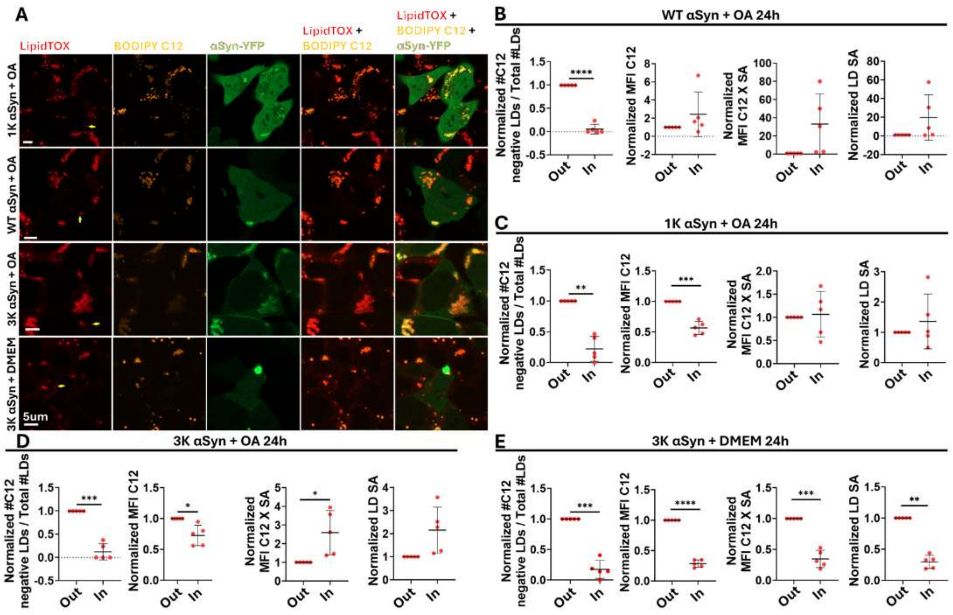

We also added the following to the text:

"Lipid droplets within αSyn condensates were consistently double-positive for C12 and LipidTOX, whereas cytoplasmic lipid droplets frequently lost C12 labeling over time. This pattern was observed across WT, 1K, and 3K αSyn. Notably, the mean fluorescence intensity (MFI) of C12 in lipid droplets entrapped within condensates was significantly reduced compared to cytoplasmic droplets in 1K and 3K cells, but not in WT. This indicates that droplets within condensates are relatively homogeneous, retaining low but uniform C12 signal, whereas cytoplasmic droplets are more heterogeneous, with subsets showing either strong retention or complete loss of C12 (fig.9A,B,C,D,E). These findings are consistent with active turnover of cytoplasmic lipid droplets, in contrast to impaired turnover of those sequestered within condensates.

Consistent with this, in 3K cells maintained in oleic acid, the integrated C12 signal (MFI × droplet surface area) was significantly higher in lipid droplets within condensates compared to those in the cytoplasm, reflecting preferential accumulation and retention. This effect was less pronounced in 1K and WT cells, suggesting a stronger sequestration phenotype in the more highly charged 3K variant. In contrast, 3K cells chased in the absence of oleic acid showed a reversal of this pattern, consistent with reduced lipid droplet availability and altered condensate association dynamics (fig.9A,B,C,D,E).

Overall, across all conditions examined, lipid droplets entrapped within αSyn condensates consistently differed from those in the cytoplasm in both C12 retention and fluorescence intensity. These results support a model in which αSyn condensates sequester lipid droplets and impair their normal turnover, potentially limiting lipid mobilization for cellular energy needs. WT and 1K cells were not analyzed under oleic acid-free pulse conditions, as they do not form inclusions in the absence of lipid droplet induction."

Western blot (Fig 2J,K): The pS129 signal for WT and 1K appears very faint-are these truly negative or just below detection? Longer exposures or more sensitive detection might help.

We note that the pS129 αSyn signal detected in WT and 1K cells is barely detectable, approaching the sensitivity limits of the assay. While it is therefore difficult to definitively distinguish between absence of phosphorylation and levels below detection, this observation is consistent with prior reports indicating that S129 phosphorylation is enriched in the 3K variant (Imberdis *et al.*, 2019; Ramalingam *et al.*, 2023a) and in more aggregated or pathological αSyn states. Importantly, our conclusions do not rely on the absence of

pS129 signal in WT or 1K cells, but rather on the relative increase observed in 3K cells. We acknowledge that more sensitive detection methods or longer exposure times could further refine these comparisons, and this represents a limitation of the current analysis.

We have included the following in the text:

"The pS129 αSyn signal detected in WT and 1K cells is probably approaching the sensitivity limits of the assay. While it is therefore difficult to definitively distinguish between absence of phosphorylation and levels below detection, this observation is consistent with prior reports indicating that S129 phosphorylation is enriched in the 3K variant (Imberdis *et al.*, 2019; Ramalingam *et al.*, 2023a) and in more aggregated or pathological αSyn states, which are exemplified by the 3K variant in this work."

Referee 3:

This study systematically investigates the role of lipid droplets in the aberrant liquid-liquid phase separation of α-synuclein and further reveals their impact on mitochondrial function and energy metabolism. Through cellular models, imaging techniques, and biochemical analysis, the authors propose that lipid droplets can serve as "nucleation sites" for abnormal αSyn aggregation, forming cytotoxic biomolecular condensates. This mechanism offers new insights into the early neuronal degeneration associated with Parkinson's disease (PD). The research is well-designed with comprehensive data and high-quality images, demonstrating strong innovation and mechanistic depth. However, certain experimental details, data interpretation, and logical coherence should be further refined.

1. All phase separation experiments in this study were conducted in cells, where the co-localization of lipid droplets with αSyn does not necessarily indicate that lipid droplets induce phase separation. It is also possible that αSyn aggregates "recruit" lipid droplets. To eliminate interference from the cellular environment, the authors should supplement their findings with relevant in vitro phase separation experiments.

We thank the reviewer for this important point. We agree that, in a cellular context, co-localization between lipid droplets and αSyn does not by itself establish directionality, and that *in vitro* reconstitution would be required to definitively dissect the underlying mechanism.

To address this question within the cellular system, we performed temporal analyses of αSyn assembly formation at early timepoints following oleic acid treatment (2-30h). We observed a progressive increase in lipid droplet abundance over time, accompanied by a corresponding increase in lipid droplet-associated αSyn assemblies, particularly in 1K cells, with a similar trend in WT cells.

While we did not directly assess the material state of these early assemblies, our data indicate that αSyn structures preferentially emerge in association with lipid droplets as lipid droplet abundance increases. This supports a model in which lipid droplets influence the nucleation and spatial organization of αSyn assemblies, rather than being passively recruited to pre-existing structures.

We note that, at later timepoints, these assemblies exhibit condensate-like properties based on FRAP and MAPAC measurements (fig.2). We have clarified this distinction in the revised manuscript. Definitive dissection of directionality and phase behavior using in vitro reconstitution will be an important direction for future work.

We have made the following changes to the manuscript:

We added 2 new figures (fig.4A,B, fig.S2A,B):

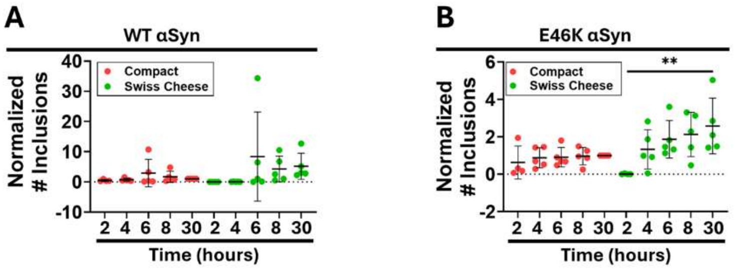

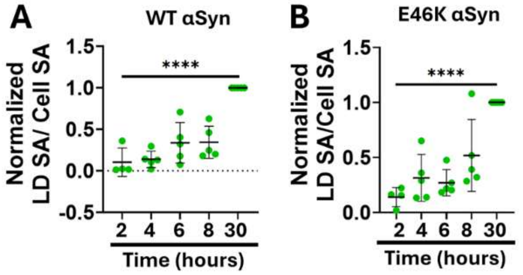

We also added the following to the text:

"To examine the relationship between lipid droplets and αSyn inclusions at early stages of formation, we performed a time-course experiment over 30h following doxycycline induction. WT and 1K αSyn-expressing cells were imaged at 2, 4, 6, 8, and 30h. Both cell lines exhibited a progressive increase in lipid droplets over time (fig.S2A,B). In parallel, 1K αSyn showed a significant increase in lipid droplet-rich ("Swiss cheese") inclusions, whereas WT αSyn showed a similar trend that did not reach significance. In contrast, lipid droplet-poor ("compact") inclusions remained unchanged over time (fig.4A,B). These findings indicate that increasing lipid droplet abundance is associated with the preferential formation of lipid droplet-rich αSyn inclusions, with a stronger effect observed for the more positively charged 1K variant. While we did not directly assess the material state of these early inclusions, at later timepoints, these assemblies exhibit condensate-like properties based on FRAP and MAPAC measurements (fig.2)."

2. In Fig. 1A, although the model is claimed to be more physiologically relevant, it lacks direct comparative validation with primary neurons or human brain tissues. It is recommended to supplement validation experiments in iPSC-differentiated dopaminergic neurons to enhance the generalizability of the results.

We agree that validation in primary neurons or iPSc-derived dopaminergic neurons would be valuable for assessing physiological relevance. The goal of the present study, however, is to establish and characterize synuclein condensate behaviour in a cell line-based system.

Accordingly, we did not extend the current study to additional cellular models. Instead, we revised the manuscript to clarify the scope and limitations of the model used here and to avoid overgeneralizing the findings beyond cell-based systems.

We added the following to the text:

"We note that the present study is conducted in a cell line-based system, which enables controlled interrogation of αSyn condensate formation and material properties. While this model provides experimental tractability, it does not fully recapitulate the cellular complexity of primary neurons or human brain tissue. In particular, neuronal-specific factors such as lipid composition, and synaptic activity may influence αSyn behavior. Validation in iPSC-derived dopaminergic neurons or primary neuronal systems will therefore be important to assess the generalizability of these findings. However, the mechanistic insights described here, particularly the relationships between lipid droplets, condensate dynamics, and mitochondrial perturbations, provide a framework that can be tested in more physiologically relevant models in future studies."

Meanwhile, Fig. 1A needs to show a 3K confocal image without OA.

We have added this column to figure 1A.

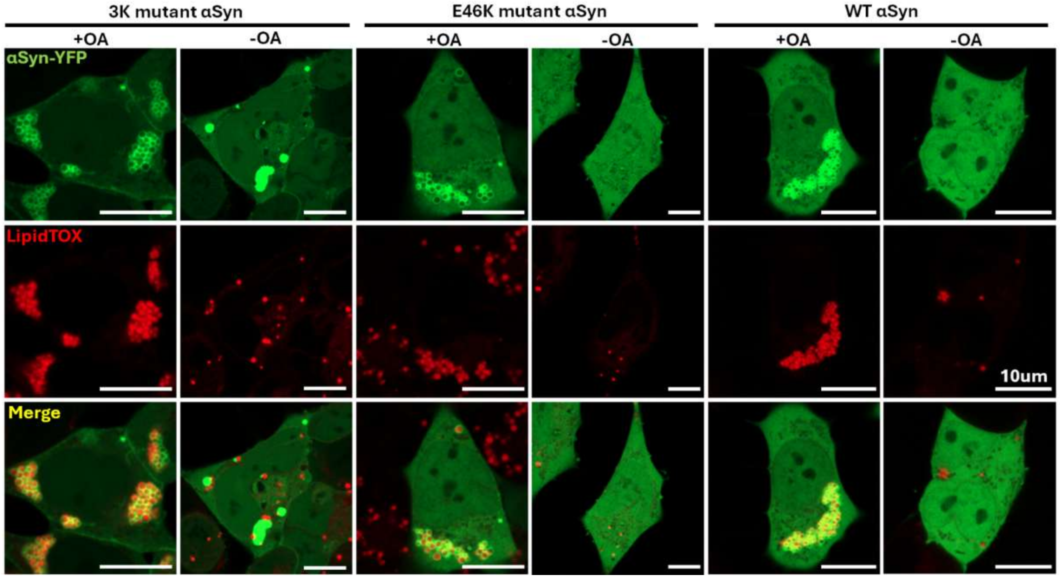

3. In Fig. 3, it is necessary to determine whether the same conclusion holds for 1K and WT α-Syn under OA treatment, especially since WT or E46K mutations are genuinely present in cells from both normal individuals and PD patients.

We thank the reviewer for this important point. To assess whether the observed mitochondrial phenotypes extend beyond the 3K variant, we performed TMRM and mitophagy analyses in WT and 1K αSyn cells under OA treatment. We found that 1K cells exhibited mitochondrial depolarization and increased mitophagy similar to 3K cells, whereas WT cells did not show significant changes under the same conditions.

These results suggest that the observed effects correlate with the positive charge of the αSyn variants, with the more highly charged 1K and 3K forms displaying more pronounced mitochondrial changes compared to WT.

In addition, as requested by Reviewer 2, we performed pulse-chase experiments in which OA was withdrawn and mitochondrial membrane potential was assessed three days later. These experiments showed recovery of mitochondrial membrane potential, indicating that the observed changes are reversible.

Together, these findings demonstrate that the mitochondrial phenotypes are not restricted to the 3K variant, but are shared by the E46K αSyn variant (also including an extra positive charge), and that these effects are dynamic rather than permanent.

We have made the following changes to the manuscript:

We added 2 new figures (fig.5C-F, fig.6):

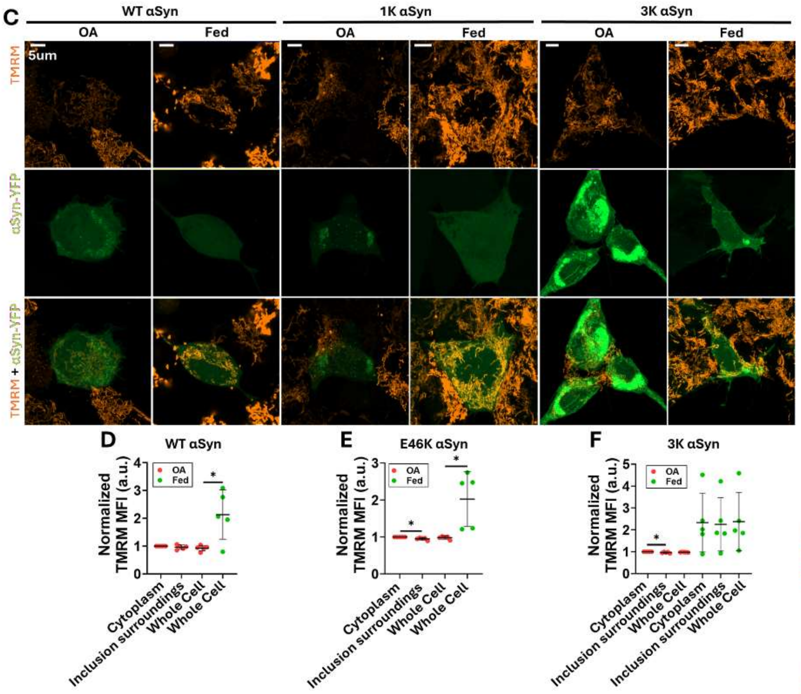

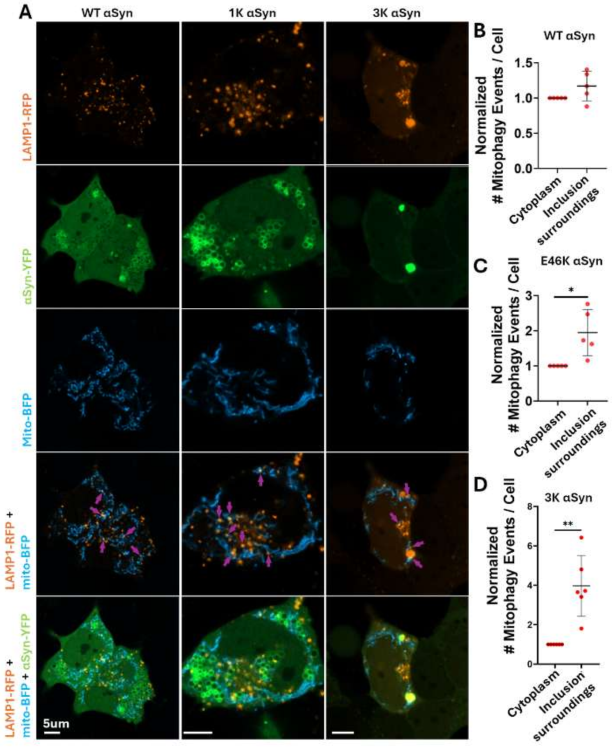

We also added the following to the text:

"We next extended this analysis to WT and 1K αSyn and compared them to 3K. Cells were induced with doxycycline for 48h, followed by oleic acid treatment for 24h, and Δψ was measured 96 h later. Parallel conditions included cells subjected to oleic acid withdrawal (pulse-chase; fig.4C-F). At the whole-cell level, withdrawal of oleic acid led to recovery of mitochondrial membrane potential across all three variants. In contrast, under continuous oleic acid treatment, mitochondria surrounding αSyn condensates in 1K and 3K cells were significantly depolarized compared to the surrounding cytoplasm. This localized depolarization was not observed in WT cells. Notably, in 3K cells subjected to oleic acid withdrawal, residual condensates persisted; however, mitochondria in their vicinity were no longer significantly depolarized (fig.5C-F).

These findings indicate that αSyn condensates can induce localized mitochondrial depolarization, with a stronger effect observed for the more positively charged 1K and 3K variants. This suggests a direct interaction between condensates and mitochondria, potentially mediated by the release of toxic αSyn species. While WT condensates do not produce a comparable local effect, all three variants exhibit some degree of global mitochondrial depolarization at the cellular level. Importantly, both condensate burden and mitochondrial dysfunction are reversible upon lipid droplet depletion. Due to the near-complete loss of condensates in WT and 1K cells following oleic acid withdrawal, recovery of local mitochondrial polarization could only be assessed in 3K cells."

"Mitochondria proximal to 1K and 3K αSyn condensates exhibited significantly higher levels of mitophagy compared to distal mitochondria, whereas no such difference was observed for WT αSyn condensates (fig.6A,B,C,D). Mitophagy events were observed both in proximity to αSyn condensates and throughout the cytoplasm, indicating that mitophagy is not exclusively spatially restricted to inclusion-containing regions. However, quantitative analysis revealed a significant enrichment of mitophagy events in the vicinity of αSyn condensates compared to distal cytoplasmic regions.

These findings are consistent with our TMRM measurements, which showed localized mitochondrial depolarization around 1K and 3K but not WT αSyn condensates (fig.5C,D,E,F), supporting the conclusion that 1K and 3K condensates induce mitochondrial damage that triggers mitophagy."

4. In Fig. 3A and B, why do α-Syn condensates cause depolarization of surrounding mitochondria? The underlying molecular mechanism requires further experimental investigation, and Fig 3D requires quantitative

analysis.

We thank the reviewer for this important question regarding the mechanism underlying mitochondrial depolarization. To address this, we performed two additional experiments (see response to Reviewer 2, main concern).

First, we inhibited autophagic flux using Bafilomycin A1. While this treatment normalized the elevated mitophagy observed around αSyn condensates, it did not rescue the localized mitochondrial depolarization. This indicates that mitophagy is not the primary cause of depolarization, but rather a downstream response.

Second, we performed correlative light and electron microscopy (CLEM), which revealed αSyn oligomers coating the outer mitochondrial membrane of mitochondria adjacent to condensates, with associated membrane disruption at sites of contact.

Together, these results support a model in which αSyn condensates directly induce mitochondrial damage, leading to depolarization, followed by secondary activation of mitophagy.

Regarding quantification, the data shown in Fig. 3D (now Fig. 6A) were previously quantified in Fig. 3C. In the revised manuscript, we have reorganized these data such that Fig. 6B-D now provide quantitative analyses corresponding to Fig. 6A.

5. In Fig. 3, the number of αsyn condensates is relatively low and they are clustered together, which differs from the condensates mentioned earlier in the text. Was this intentionally selected to investigate the impact of condensates on mitochondria? A description is required to explain this discrepancy.

We agree that clarification is helpful. Condensates in Fig. 3 (now figures 5 and 6) were not selected based on number, size, or spatial arrangement; rather, all detectable synuclein condensates present in the acquired images were included in the quantifications. The images shown were chosen as representative examples illustrating the phenotypes observed across experiments.

We have now replaced these images as these experiments were expanded to also include WT and 1K cells.

6. The authors repeatedly describe αSyn condensates as "viscoelastic" or "gel-like" without providing any rheological measurements or mechanical characterization. These terms have precise physical meanings and should not be used based solely on FRAP immobile fractions or morphological appearance. Please remove or soften these physical descriptors (e.g., "partially dynamic" or "low mobility") unless relevant experimental data are provided to support viscoelastic or gel-like behavior.

Please see response to comment #6 by reviewer 1. We undertook micropipette aspiration with patch clamp (MAPAC), which is a novel technique used to quantify the viscoelastic properties of condensates (Wang et al., 2025). Using MAPAC, we found that 3K αSyn condensates formed under both OA- and OA+ conditions exhibit viscoelastic properties, with OA treatment consistently shifting condensates toward a more viscous and less fluid state.

Referee 4:
Manuscript number: EMBOR-2025-63242V1

This is an interesting manuscript exploring how αSyn liquid-liquid phase separation can be influence by lipid droplets. LLPS has become an important and emerging area of protein science, however, how condensate formation is triggered and the connection this has to neurodegeneration remain unclear. Furthermore, the contribution of lipid droplets towards phase separation processes is unknown. The authors explore this exciting topic with cell-based experiments. The experiments are robust and of high quality. My main concern is regarding whether the fluorescent protein is influencing the phase separation of αSyn. The authors are using αSyn-YFP. Given that αSyn is around half the size of YFP, I wonder what the effect of YFP is on the phase separation behaviour of αSyn. It is known that the size of biomolecules can strongly affect phase separation. I therefore recommend the authors perform experiments with another fluorescent system, to distinguish whether the YFP contributes to the phase separation of αSyn. This would really enhance the quality of the paper and help clarify things.

We thank the reviewer for this important and thoughtful point. We agree that the use of a YFP tag could, in principle, influence the biophysical behavior of αSyn, particularly given the relative size of the fluorescent protein and the known sensitivity of LLPS.

However, multiple independent studies have demonstrated that αSyn undergoes phase separation across a range of labeling strategies, including GFP/YFP-tagged constructs as well as minimally labeled or untagged protein *in vitro*, *in vivo*, and in cultured cells (Agarwal et al, 2024; Hardenberg et al, 2021; Wang et al, 2016), supporting that LLPS is an intrinsic property of αSyn rather than an artifact of fluorescent tagging.

Importantly, in our system, all constructs are uniformly tagged, and the key conclusions are based on relative differences between conditions. We observe that condensate formation is strongly modulated by lipid droplet abundance and by the charge state of αSyn (WT vs 1K vs 3K), indicating that these intrinsic and cellular factors, rather than the fluorescent tag, are the primary drivers of the observed behavior.

While we cannot fully exclude quantitative effects of the YFP tag on condensate properties, the consistency of our findings with prior studies and the internal modulation of the phenotype support the robustness of our conclusions. We have now clarified this point in the Discussion and highlighted the use of alternative labeling strategies as an important direction for future work.

We added the following to the text:

“A limitation of our study is the use of a YFP tag on αSyn, which could in principle influence its biophysical behavior, including LLPS. This is particularly relevant given that αSyn is substantially smaller than YFP, and protein size and valency can modulate LLPS. However, multiple independent studies have demonstrated that αSyn undergoes phase separation across a range of labeling strategies, including GFP/YFP-tagged constructs as well as minimally labeled or untagged protein *in vitro*, *in vivo*, and in cultured cells (Agarwal *et al.*, 2024; Hardenberg *et al.*, 2021; Wang *et al.*, 2016). These findings support that LLPS is an intrinsic property of αSyn rather than an artifact of fluorescent tagging. Importantly, in our system, condensate formation is strongly modulated by lipid droplet abundance and αSyn mutation/charge state, arguing against a dominant contribution from the fluorescent tag. While we cannot fully exclude effects of the YFP tag on condensate properties, the consistency of our observations with prior studies and the internal modulation of the phenotype support the robustness of our conclusions.”

My other points are listed below:

1) In the Results section, the authors conduct FRAP experiments on the αSyn inclusions. They state that "Interestingly, 3K αSyn inclusions that formed spontaneously, without addition of oleic acid, were significantly more liquid than the more lipid droplet-rich 3K inclusions formed after treatment with oleic acid (fig.1F,G,H,I)." Could it be that the lipid rich inclusions are less liquid-like simply because they are denser, as there is more lipid present. The authors should elaborate on this and discuss whether they believe their observation is due to another factor.

We have added the following discussion to address this point:

“Interestingly, lipid droplet-rich 3K αSyn condensates exhibited reduced fluorescence recovery compared to those formed in the absence of lipid-rich enrichment. While this could in part reflect increased density due to the incorporation of lipids, we favor a model in which lipid droplets actively modulate condensate material properties. Lipid interfaces provide a scaffold that enhances multivalent αSyn-lipid and αSyn-αSyn interactions, thereby increasing viscosity and promoting a transition toward a more gel-like state. Thus, the observed reduction in liquidity likely reflects an interaction-driven change in material state.”

2) How long after the αSyn inclusions were formed did the authors conduct the FRAP experiments? I suggest the authors conduct the FRAP experiments at different time points after the inclusions are formed. In this way, they can explore how the inclusions transition from liquid to solid.

In figure 2B,C (formerly figure 1G,H), we did the FRAP measurements at 72h post doxycycline induction (48h of doxycycline induction followed by 24h of OA incubation).

We undertook additional timecourse FRAP measurements starting at 48h post-doxycycline induction; we measured FRAP every 24h over 4 days. This data is included in the response to reviewer 1 comment #6. Briefly, we found that WT and 1K condensates don't show a change in immobile fraction over time, as opposed to 3K condensates that become more solid (Eubanks *et al.*, 2025).

3) Could the authors show magnified images of the Swiss cheese and compact condensates so that the reader can see the two different types. On that note, do the authors have any mechanistic insights into why one type is formed over the other? Some discussion on this would be helpful.

We have included magnified images of Swiss cheese and compact condensates in figure 1B,C.

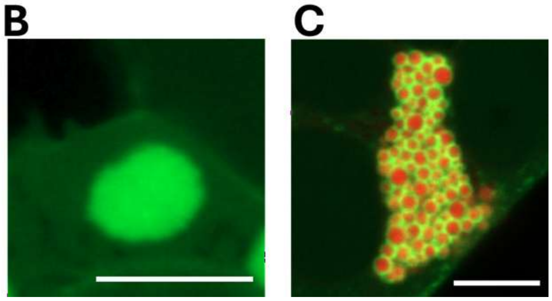

We have also added the following text to the discussion:

"We observed two morphologically distinct types of αSyn condensates: lipid droplet-rich "Swiss cheese" condensates and more compact condensates lacking visible lipid inclusions. We propose that these represent different types of αSyn-lipid interaction. Swiss cheese condensates likely arise in lipid-rich environments where αSyn binds to and stabilizes lipid droplets, leading to their incorporation into the condensate. In contrast, compact condensates may form under conditions of lower lipid availability or altered interaction strength, where protein-protein interactions dominate. This interpretation is supported by our dose-response data, where increasing lipid droplet abundance selectively expands the Swiss cheese population without significantly affecting compact condensates."

4) As the authors correctly mention, phosphorylation of αSyn is rather complicated and it is unclear how it contributes to pathology. In fact, it appears that phosphorylated αSyn aggregates slower than WT αSyn. Could the authors elaborate further on this and expand on the relevance of this in the context of their experiments. If phosphorylated αSyn is slower in aggregating, how are the data presented in this manuscript linked to pathology.

We have added the following to the discussion:

"The role of S129 phosphorylation in αSyn biology remains complex and context-dependent. While phosphorylation at S129 has been reported to reduce fibrillization kinetics in some systems, it is also strongly associated with pathological inclusions *in vivo* (Ghanem *et al*, 2022; Oueslati, 2016). In our model, lipid droplet-rich 3K αSyn condensates exhibit increased S129 phosphorylation. There are several explanations for this observation. It is possible that the lipid droplets act as a nidus to which αSyn binds and phase separates, which in turn facilitates aggregation and subsequent S129 phosphorylation. Alternatively, αSyn associated with lipid droplets may be more accessible to kinases, leading to preferential phosphorylation within these condensates (Ramalingam et al, 2023b). This increase in S129 phosphorylation may contribute to the observed shift toward a more viscous, less dynamic material state in lipid droplet-rich condensates. However, these assemblies remain in a semi-solid regime rather than progressing to fully solid aggregates (Hardenberg et al., 2021)."

5) The authors state both that lipid droplets induce αSyn phase separation and that the αSyn condensates themselves entrap lipid droplets. This is a confusing mechanism. The authors should elaborate more on this point. This is quite important as it is a central aspect of their manuscript.

We agree that clearer explanation of this point is important. We propose that lipid droplets nucleate the formation of synuclein condensates and get entrapped within them during the process.

We have added the following to the discussion:

"At first glance, the observation that lipid droplets both promote αSyn phase separation and are subsequently incorporated into αSyn condensates may appear paradoxical. However, we propose a bidirectional model in which lipid droplets initially act as nucleation platforms that locally concentrates αSyn and promotes phase separation. Once condensates are formed, they in turn sequester the lipid droplets through sustained protein-lipid interactions. This creates a self-reinforcing cycle whereby lipid droplets facilitate condensate formation, and condensates progressively trap lipid droplets, ultimately impairing their turnover."

We have included the graphical abstract of our proposed model below as figure 10.

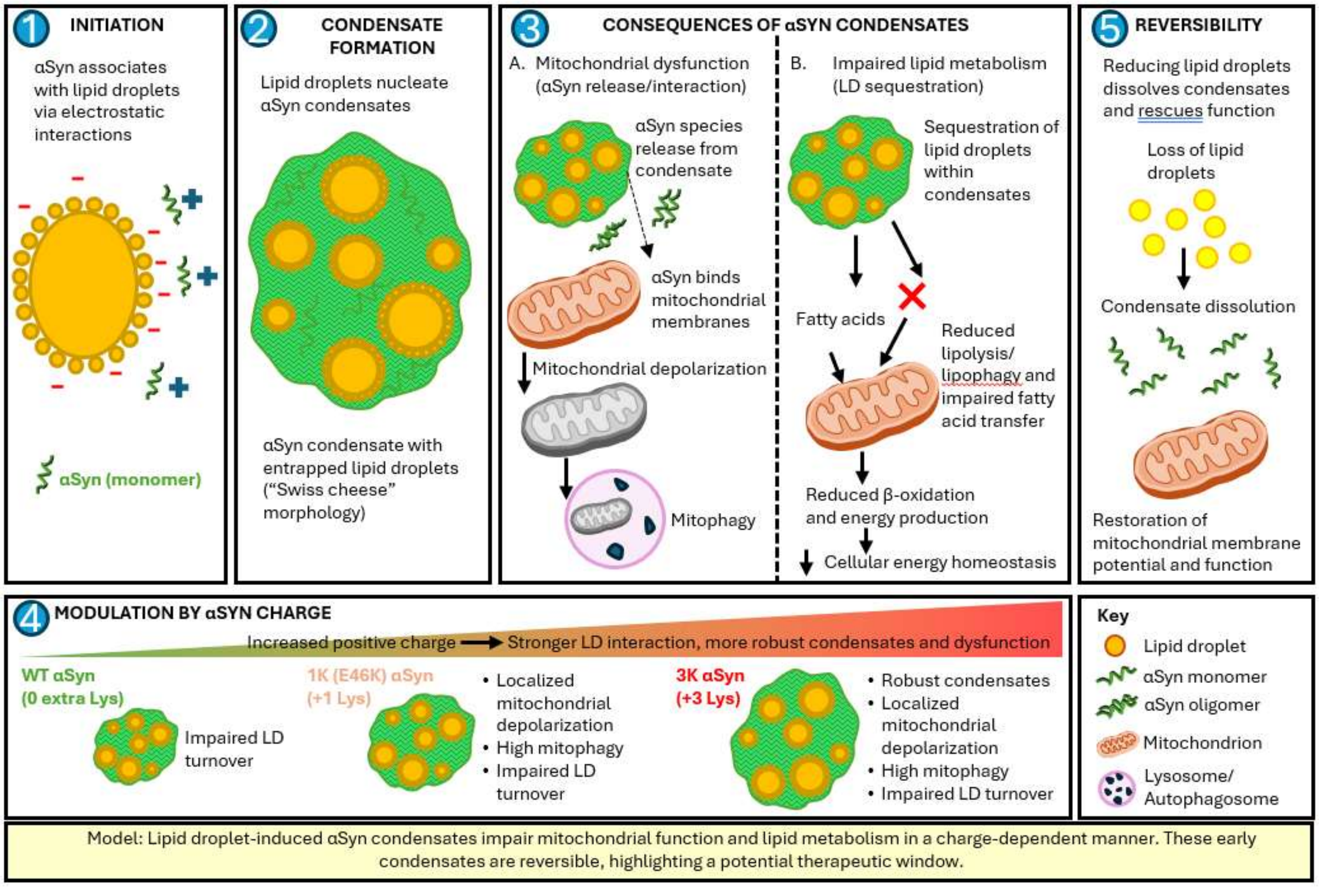

6) Start of the second paragraph of the Discussion; I suggest the authors rephrase this and don't make this sentence so absolute. More evidence needs to be accumulated before this statement can be confidently made. This comment also relates to Figure 5B-C, use of the word toxic may not be fully appropriate given the experiments so far. If the authors conducted further studies complementing their current mitochondrial experiments, then such terminology can be used.

We have toned down the discussion and removed the word toxic from figure 10 (formerly figure 5).

Minor points:

7) At the end of the first paragraph of the Introduction, it should be noted that liquid-to-solid transitions of other neurodegenerative related proteins such as amyloid-beta have also been investigated (e.g. https://doi.org/10.1038/s41598-024-72265-7). This should be included.

We cited this paper and included the relevant information in the introduction.

"This phenomenon has been well studied for tau (Boyko et al, 2019; Hochmair et al, 2022; Wegmann et al, 2018) and TAR DNA-binding protein 43 (TDP-43) (Schmidt & Rohatgi, 2016; Yan et al, 2024) and also observed for amyloid-beta (Morris et al, 2024)."

8) In the second paragraph of the Introduction, the first sentence has no reference. A reference to back the claim should be added.

We added references for this sentence.

"There is recent evidence suggesting that aberrant LLPS of alpha-synuclein (αSyn) is involved in its dysregulation in Parkinson's disease (PD) and Dementia with Lewy bodies (DLB), eventually leading to the formation of Lewy bodies (Agarwal *et al.*, 2024; Dada *et al*, 2023; Eubanks *et al.*, 2025; Hardenberg *et al.*, 2021; Mukherjee *et al*, 2023; Ray *et al*, 2020; Wang *et al*, 2024)."

I recommend the authors revise their manuscript based on my comments above before the manuscript can be accepted for publication.

Cross-comments from referee 1:

Compared with the other two reviewers, our review may be somewhat stricter. However, after reading their reports in full, it is clear that our major concerns essentially align with those raised by Reviewers 2 and 3: the evidence for mitochondrial dysfunction and mitophagy is weak (Reviewer 2 - major comment; Reviewer 3 - comments 4 and 5), the causal relationship between oleic acid (lipid droplets) and α-synuclein condensates remains unclear (Reviewer 3 - comment 1), and the assessment of α-synuclein condensate properties, such as the FRAP assay, is insufficient (Reviewer 3 - comment 6). The major point of divergence is that Reviewers 2 and 3 did not address the differences in α-synuclein expression levels among the WT, 1K, and 3K cell lines. Since many of the main figures compare these mutant cell lines, we believe this issue cannot be overlooked.

Cross-comments from referee 2:

I agree with Reviewer #1's comments regarding mitophagy and concur that the authors need to provide additional evidence to support a direct link between LLPS and mitophagy. Overall, this is an interesting manuscript that is worthy of publication.

Cross-comments from referee 3:

In general, the concerns from Reviewer 1 are reasonable. However, I feel the story remains interesting and has novel aspects. If the authors can address these concerns by adding/replacing sufficient new experiments, I still recommend publication.

Additional changes:

1) In the initially submitted version of the manuscript, figure 1J (now figure 2G) (hexanediol treatment) included the data analyzed on a per image basis. To ensure consistency with all the other data in the paper, we have replaced this with a new graph showing the data analyzed on a single cell basis. The difference between untreated and 1.5% hexanediol treatment is significant for both cell lines, therefore the conclusions remain unchanged.

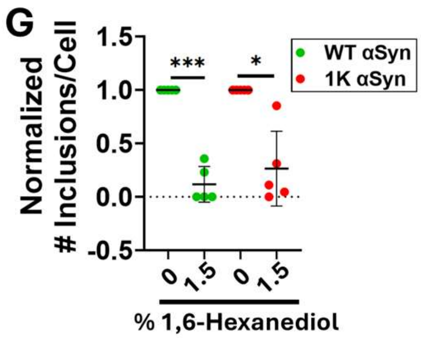

2) After we submitted our paper in November 2025, we became aware of a preprint from a different group on a similar topic. This group used *C. elegans* and lipidomics to show that αSyn condensates are linked to alterations in lipid metabolism. We have now cited this paper in the discussion and discussed how it complements our findings.

We have added the following section to the discussion:

“Recent work has also linked αSyn condensates to alterations in lipid metabolism, including changes in triglyceride homeostasis (Zhang et al, 2025). While conceptually related, our findings identify lipid droplets as an early driver of αSyn condensate formation and demonstrate that these condensates exert direct, localized effects on organelle function, including impaired lipid droplet turnover and mitochondrial depolarization. Thus, our work provides a cellular and organelle-level mechanistic framework that complements and extends these observations.”

References

Agarwal A, Chandran A, Raza F, Ungureanu IM, Hilcenko C, Stott K, Bright NA, Morone N, Warren AJ, Lautenschlager J (2024) VAMP2 regulates phase separation of alpha-synuclein. *Nat Cell Biol*
Alam SR, Wallrabe H, Christopher KG, Siller KH, Periasamy A (2022) Characterization of mitochondrial dysfunction due to laser damage by 2-photon FLIM microscopy. *Sci Rep* 12: 11938
Boyko S, Qi X, Chen TH, Surewicz K, Surewicz WK (2019) Liquid-liquid phase separation of tau protein: The crucial role of electrostatic interactions. *J Biol Chem* 294: 11054-11059
Dada ST, Hardenberg MC, Toprakcioglu Z, Mrugalla LK, Cali MP, McKeon MO, Klimont E, Michaels TCT, Knowles TPJ, Vendruscolo M (2023) Spontaneous nucleation and fast aggregate-dependent proliferation of alpha-synuclein aggregates within liquid condensates at neutral pH. *Proc Natl Acad Sci U S A* 120: e2208792120
Eubanks E, VanderSleen K, Mody J, Patel N, Sacks B, Farahani MD, Wang J, Elliott J, Jaber N, Akcimen F *et al* (2025) Increased burden of rare risk variants across gene expression networks predisposes to sporadic Parkinson's disease. *Cell Rep* 44: 115636
Ghanem SS, Majbour NK, Vaikath NN, Ardah MT, Erskine D, Jensen NM, Fayyad M, Sudhakaran IP, Vasili E, Melachroinou K *et al* (2022) alpha-Synuclein phosphorylation at serine 129 occurs after initial protein deposition and inhibits seeded fibril formation and toxicity. *Proc Natl Acad Sci U S A* 119: e2109617119
Hardenberg MC, Sinnige T, Casford S, Dada ST, Poudel C, Robinson EA, Fuxreiter M, Kaminksi CF, Kaminski Schierle GS, Nollen EAA *et al* (2021) Observation of an alpha-synuclein liquid droplet state and its maturation into Lewy body-like assemblies. *J Mol Cell Biol* 13: 282-294
Hochmair J, Exner C, Franck M, Dominguez-Baquero A, Diez L, Brognaro H, Kraushar ML, Mielke T, Radbruch H, Kaniyappan S *et al* (2022) Molecular crowding and RNA synergize to promote phase separation, microtubule interaction, and seeding of Tau condensates. *EMBO J* 41: e108882
Imberdis T, Negri J, Ramalingam N, Terry-Kantor E, Ho GPH, Fanning S, Stirtz G, Kim TE, Levy OA, Young-Pearse TL *et al* (2019) Cell models of lipid-rich alpha-synuclein aggregation validate known modifiers of alpha-synuclein biology and identify stearoyl-CoA desaturase. *Proc Natl Acad Sci U S A* 116: 20760-20769

Kumar M, Wu Y, Knapp J, Pontius CL, Park D, Witte RE, McAllister R, Gupta K, Rajagopalan KN, De Camilli P, Ryan TA (2025) Triglycerides are an important fuel reserve for synapse function in the brain. *Nat Metab* 7: 1392-1403

Morris OM, Toprakcioglu Z, Rontgen A, Cali M, Knowles TPJ, Vendruscolo M (2024) Aggregation of the amyloid-beta peptide (Abeta40) within condensates generated through liquid-liquid phase separation. *Sci Rep* 14: 22633

Mukherjee S, Sakunthala A, Gadhe L, Poudyal M, Sawner AS, Kadu P, Maji SK (2023) Liquid-liquid Phase Separation of alpha-Synuclein: A New Mechanistic Insight for alpha-Synuclein Aggregation Associated with Parkinson's Disease Pathogenesis. *J Mol Biol* 435: 167713

Oueslati A (2016) Implication of Alpha-Synuclein Phosphorylation at S129 in Synucleinopathies: What Have We Learned in the Last Decade? *J Parkinsons Dis* 6: 39-51

Papadopoulos C, Orso G, Mancuso G, Herholz M, Gumeni S, Tadepalle N, Jungst C, Tzschichholz A, Schauss A, Honing S *et al* (2015) Spastin binds to lipid droplets and affects lipid metabolism. *PLoS Genet* 11: e1005149

Ramalingam N, Brontesi L, Jin SX, Selkoe DJ, Dettmer U (2023a) Dynamic reversibility of alpha-synuclein serine-129 phosphorylation is impaired in synucleinopathy models. *EMBO Rep* 24: e57145

Ramalingam N, Jin SX, Moors TE, Fonseca-Ornelas L, Shimanaka K, Lei S, Cam HP, Watson AH, Brontesi L, Ding L *et al* (2023b) Dynamic physiological alpha-synuclein S129 phosphorylation is driven by neuronal activity. *NPJ Parkinsons Dis* 9: 4

Ray S, Singh N, Kumar R, Patel K, Pandey S, Datta D, Mahato J, Panigrahi R, Navalkar A, Mehra S *et al* (2020) alpha-Synuclein aggregation nucleates through liquid-liquid phase separation. *Nat Chem* 12: 705-716

Schmidt HB, Rohatgi R (2016) In Vivo Formation of Vacuolated Multi-phase Compartments Lacking Membranes. *Cell Rep* 16: 1228-1236

Wang C, Zhang K, Cai B, Haller JE, Carnazza KE, Hu J, Zhao C, Tian Z, Hu X, Hall D *et al* (2024) VAMP2 chaperones alpha-synuclein in synaptic vesicle co-condensates. *Nat Cell Biol*

Wang H, Becuwe M, Housden BE, Chitraju C, Porras AJ, Graham MM, Liu XN, Thiam AR, Savage DB, Agarwal AK *et al* (2016) Seipin is required for converting nascent to mature lipid droplets. *Elife* 5

Wang H, Hoffmann C, Tromm JV, Su X, Elliott J, Wang H, Deng M, McClenaghan C, Baum J, Pang ZP *et al* (2025) Live-cell quantification reveals viscoelastic regulation of synapsin condensates by alpha-synuclein. *Sci Adv* 11: eads7627

Wegmann S, Eftekharzadeh B, Tepper K, Zoltowska KM, Bennett RE, Dujardin S, Laskowski PR, MacKenzie D, Kamath T, Commins C *et al* (2018) Tau protein liquid-liquid phase separation can initiate tau aggregation. *EMBO J* 37

Yan X, Kuster D, Mohanty P, Nijssen J, Pombo-Garcia K, Rizuan A, Franzmann TM, Sergeeva A, Passos PM, George L *et al* (2024) Intra-condensate demixing of TDP-43 inside stress granules generates pathological aggregates. *bioRxiv*

Zhang T, Goya ME, Herron-Bedoya A, van der Weerd JC, Tsagkari D, Couzijn S, Gungordu L, Seinstra RI, Heiner Fokkema MR, Chang M *et al* (2025) Condensate-Driven Triglyceride Depletion Links alpha-Synuclein to Mitochondrial Dysfunction. *bioRxiv*

Dear Eleanna,

Thank you for the submission of your revised manuscript. We have now received the enclosed reports from all referees and I am happy to say that all support its publication now. Referee 1 still has some more comments and suggestions that I would like you to address and incorporate before we can proceed with the official acceptance of your manuscript.

Please let me know if you have any questions regarding referee 1's comments and we can discuss this further, also in a video chat, if you wish. Please also submit a point-by-point response to all comments with your final ms file.

A few editorial requests will also need to be addressed:

- Please correct the conflict of interest subheading to "Disclosure and Competing Interests Statement"

- The author credits need to be removed from the ms file. All credits need to be entered during online ms submission.

- The FUNDING INFO needs to be congruent in the ms file and in our online submission system. The following funders are missing in our online system: Kuckein Student Research Fellowship, Aresty summer science research fellowship, Rutgers Health Center for Biomedical Informatics & Health Artificial Intelligence (BMIHAI) summer research internship, & Health Artificial Intelligence (BMIHAI) summer research internship (SJ, MG), Rutgers start-up funding. Please add all funding info with your next online ms submission.

- The callouts for the individual panels of Figure 8 (A-D) are missing. The callouts for the EV figures are not correct: it needs to be Figure EV1, instead EV figure 1, etc.

- The Appendix file can be deleted, and you can have 7 EV figures instead, which should make it easier. The first Appendix figure seems to be source data and can therefore be deleted (if this is also uploaded to BioStudies. If not, it should be a Source Data file instead).

- The Movie legends need to be removed from the ms file and each should be provided in a text file; then each legend should be zipped up with its movie so that we have two zip files: Movie EV1 and Movie EV2; the correct nomenclature in all places should be Movie EV1, Movie EV2 - there are some instances of EV movie 1, EV movie 2 - this needs to be corrected.

- Please remove the Reagents & Tools table from the ms file and upload it as a separate file.

- The manuscript sections should be in the following order: Title page - Abstract - Introduction - Results - Discussion - Methods - Data Availability - Acknowledgments - Disclosure Statement & Competing Interests - References - Figure Legends - (Main Tables with legends if applicable) - Expanded View Figure Legends

- Our routine image analysis detected a possible splice site in Appendix Fig S5B that is not mentioned in the figure legend. Can you please clarify?

* Figure Legends - Comments *

- Please note that the exact p values are not provided in the legends of figures 1D, E, F, G, I; 2B, C, G; 3G-I; 4A, B, D, E, F, G; 5B, D, E; 6C, D; 7A, C; 9B, C, D, E; EV1 B, D, E; EV2 A-C. Please provide exact values as reasonable.
- Please note that the box plots need to be defined in terms of minima, maxima, centre, bounds of box and whiskers, and percentile in the legend of figure 2L
- Please note that information related to n is missing in the legend of figure 2C
- Please note that the dashed line is not defined in the legends of figurse 2J, K. This needs to be rectified.

I would like to suggest some minor changes to the title and abstract that needs to be written in present tense. Do you agree with this:

Lipid droplets promote aberrant liquid-liquid phase separation of alpha-synuclein impairing energy homeostasis

... In this study, we show that wild type (WT) and the PD-associated E46K mutant αSyn phase separate into condensates in the presence of lipid droplets. WT and E46K mutant lipid droplet-rich αSyn condensates prevent turnover of entrapped lipid droplets. E46K mutant condensates damage neighbouring mitochondria which are depolarized and undergo increased mitophagy [Is this a direct effect or a correlation? I think this needs to be re-written to better reflect the data]. These findings underscore the importance of lipid droplets in neurodegenerative diseases, and PD in particular. Lipid droplets are enriched within neuromelanin

in midbrain dopaminergic neurons in the substantia nigra and could therefore facilitate αSyn-associated neurodegeneration in this region in PD. Our findings reveal a novel pathway implicated in αSyn dysregulation linking aberrant liquid-liquid phase separation, lipid droplets and mitochondrial damage.

EMBO press papers are accompanied online by A) a short (1-2 sentences) summary of the findings and their significance, B) 2-3 bullet points highlighting key results and C) a synopsis image that is exactly 550 pixels wide and 300 pixels high. The synopsis image should provide a sketch of the major findings, like a graphical abstract. Please note that text needs to be readable at the final size. Please send us this information along with the final manuscript.

I look forward to seeing a final version of your manuscript as soon as possible.

Best regards,
Esther

Esther Schnapp, PhD
Senior Editor
EMBO reports

Referee #1:

The reviewer greatly appreciates the extensive revisions and the substantial amount of new experimental data provided by the authors. The manuscript has been significantly improved, and the authors have thoroughly addressed the major mechanistic concerns raised in the previous round. To further enhance the readability and technical rigor of this study, the following final points are kindly suggested for consideration.

1. Enhancing Readability and Figure Referencing
With the addition of several new experimental panels, the manuscript has become substantially more data-rich. To further improve readability and ease of navigation, it would be helpful if the authors could clearly specify the corresponding figure and panel number for each result described in the text, rather than referring to multiple figures or panels collectively.

2. Optimizing Western Blot for Total aSyn (Fig. 3K)
The analysis in Figure 1, using cells whose YFP fluorescence levels were matched, is excellent and highly commendable. However, there remains a concern regarding Figure 3K: both the total and phosphorylated aSyn signals in WT and 1K cells appear quite faint and almost undetectable. To completely clear any ambiguity regarding the baseline expression levels, it would be beneficial if the authors could optimize this Western blot-perhaps by increasing the exposure time-so that the total aSyn signals in WT and 1K cells becomes visible. Providing a clear panel showcasing total aSyn with an appropriate loading control would further support the presence of the protein.

3. Refining Methodology and Logical Consistency in Figure EV2A
Figure EV2A provides an insightful spatial analysis, but it would benefit from refinement to ensure it is fully informative and clear for the readership. First, the current X-axis label "distance from inclusion" might be slightly misleading. Since the figure legend indicates that the analysis defines two distinct domains-the expanded inclusion rim and the remaining cytoplasm-by step-by-step expansion, the X-axis label would be more accurate if revised to reflect the actual parameter, such as Expansion distance from inclusion boundary (µm). Additionally, please convert the "pixels" into standard physical units (µm) to provide better biological context. Second, since the cell is divided into these two regions at each step, the sum of the mitophagy events from both regions would be expected to remain relatively constant across all X values, as their total must always equal the whole-cell mitophagy events. However, this does not appear to be the case in the current graph. In particular, since cells often contain multiple inclusions, how did the analysis handle potential spatial overlaps when the boundaries of neighboring inclusions expanded and intersected? Clarification of these points, including the apparent non-constant sum of mitophagy events and the handling of overlapping regions, would help readers better understand the methodology.
Furthermore, the authors conclude that a 25-pixel distance is appropriate, yet the graph shows a complete plateau from 9 to 30 pixels. To strengthen the rationale, it would be helpful if the authors could further explain why 25 pixels was selected, as the observed effect already appears to have reached saturation at 9 pixels.

4. Clarifying the Conceptual Model (Figure 10)
To avoid any potential confusion for the readership, we suggest that the conceptual model in Figure 10 clearly distinguish between experimentally supported and speculative mechanisms. Since some specific events were not biochemically measured in this study, these unverified events should either be removed from the diagram or clearly illustrated as speculative, for example by using dashed arrows or labeling them as hypothetical.

Referee #2:

My concerns have been addressed.

Referee #3:

The authors addressed all my points. Thus I recommend this manuscript for publication.

Referee #4:

The authors have addressed all my concerns. The manuscript is well written, presents exciting data, and is quite interesting. I recommend this paper be published.

POINT BY POINT RESPONSE

Please find below our responses to the editorial comments and the additional comments of reviewer #1. Changes in the text are highlighted in green.

EDITORIAL COMMENTS

- Please correct the conflict of interest subheading to "Disclosure and Competing Interests Statement"

Done.

- The author credits need to be removed from the ms file. All credits need to be entered during online ms submission.

Done.

- The FUNDING INFO needs to be congruent in the ms file and in our online submission system. The following funders are missing in our online system: Kuckein Student Research Fellowship, Aresty summer science research fellowship, Rutgers Health Center for Biomedical Informatics & Health Artificial Intelligence (BMIHAI) summer research internship, & Health Artificial Intelligence (BMIHAI) summer research internship (SJ, MG), Rutgers start-up funding. Please add all funding info with your next online ms submission.

The Kuckein Student Research Fellowship was already included as Alpha Omega Alpha Honor Medical Society in the online submission system. We have now included the full name of the funder in the manuscript. We have added the rest of the funders to the online submission system.

- The callouts for the individual panels of Figure 8 (A-D) are missing. The callouts for the EV figures are not correct: it needs to be Figure EV1, instead EV figure 1, etc.

We have corrected this.

- The Appendix file can be deleted, and you can have 7 EV figures instead, which should make it easier. The first Appendix figure seems to be source data and can therefore be deleted (if this is also uploaded to BioStudies. If not, it should be a Source Data file instead).

We have converted all figures to EV figures. We have uploaded the western blot figures to BioImage Archive, but we decided to keep in the first appendix figure because reviewer 1 requested an additional exposure image for that western blot, which we included as additional panels. We have also added titles to all EV figures.

- The Movie legends need to be removed from the ms file and each should be provided in a text file; then each legend should be zipped up with its movie so that we have two zip files: Movie EV1 and Movie EV2; the correct nomenclature in all places should be Movie EV1, Movie EV2 - there are some instances of EV movie 1, EV movie 2 - this needs to be corrected.

Done.

- Please remove the Reagents & Tools table from the ms file and upload it as a separate file.

Done.

- The manuscript sections should be in the following order: Title page - Abstract - Introduction - Results -

Discussion - Methods - Data Availability - Acknowledgments - Disclosure Statement & Competing Interests - References - Figure Legends - (Main Tables with legends if applicable) - Expanded View Figure Legends

Done.

- Our routine image analysis detected a possible splice site in Appendix Fig S5B that is not mentioned in the figure legend. Can you please clarify?

We thank the editor for this observation. The apparent splice site in Appendix Fig. S5B (now figure EV 8) corresponds to the boundary between two adjacent low-magnification electron microscopy tiling images that were stitched together to generate the final mosaic. It does not reflect image manipulation of the underlying data. We have clarified this point in the figure legend.

We have made the following change to the manuscript:

Figure EV 8

The image in Panel B is a stitched tiles of low-magnification EM images. The vertical line represents the boundary between the two images.

* Figure Legends - Comments *

- Please note that the exact p values are not provided in the legends of figures 1D, E, F, G, I; 2B, C, G; 3G-I; 4A, B, D, E, F, G; 5B, D, E; 6C, D; 7A, C; 9B, C, D, E; EV1 B, D, E; EV2 A-C. Please provide exact values as reasonable.

We have added the exact p-values to the corresponding figures. They were already included in the GraphPad prism files provided with the source data. Some p-values that were <0.0001 are still designated as ****, which is elaborated upon in the figure legend. The name of the statistical test used was missing for some of the panels and we also added this to the legend.

- Please note that the box plots need to be defined in terms of minima, maxima, centre, bounds of box and whiskers, and percentile in the legend of figure 2L.

We have added the following to the legend of figure 2L:

"The center line indicates the median; the box bounds represent the 25th and 75th percentiles; the whiskers extend to the most extreme data points within 1.5 × the interquartile range; and each point represents an independent measurement."

- Please note that information related to n is missing in the legend of figure 2C

We have added the following to the legend of figure 2C:

"(N: 3K no OA = 87; 3K OA = 75; 1K OA = 81; WT OA = 63)"

- Please note that the dashed line is not defined in the legends of figures 2J, K. This needs to be rectified.

We have added the following to the legend of figure 2J,K:

"The dashed line indicates the position of aspirated condensate inside the micropipette after pressure application. It is used to visually mark the extent of condensate deformation/climbing into the pipette under the applied aspiration pressure."

I would like to suggest some minor changes to the title and abstract that needs to be written in present tense.

Do you agree with this:

Lipid droplets promote aberrant liquid-liquid phase separation of alpha-synuclein impairing energy homeostasis

We agree with the change in title.

... In this study, we show that wild type (WT) and the PD-associated E46K mutant αSyn phase separate into condensates in the presence of lipid droplets. WT and E46K mutant lipid droplet-rich αSyn condensates prevent turnover of entrapped lipid droplets. E46K mutant condensates damage neighbouring mitochondria which are depolarized and undergo increased mitophagy [Is this a direct effect or a correlation? I think this needs to be re-written to better reflect the data]. These findings underscore the importance of lipid droplets in neurodegenerative diseases, and PD in particular. Lipid droplets are enriched within neuromelanin in midbrain dopaminergic neurons in the substantia nigra and could therefore facilitate αSyn-associated neurodegeneration in this region in PD. Our findings reveal a novel pathway implicated in αSyn dysregulation linking aberrant liquid-liquid phase separation, lipid droplets and mitochondrial damage.

We have included the new abstract below:

Alpha-synuclein (αSyn) inclusions are a defining neuropathological feature of Parkinson's disease, but the cellular events that initiate their formation and promote neurotoxicity remain incompletely understood. Aberrant liquid-liquid phase separation has emerged as a potential early step in αSyn dysregulation, yet the physiological triggers and functional consequences of this process are unclear. Here, we show that lipid droplets promote the spontaneous phase separation of wild-type and E46K mutant αSyn into condensates. These condensates sequester lipid droplets and impair their turnover, indicating disruption of cellular lipid homeostasis. Mitochondria in close proximity to αSyn condensates exhibit reduced membrane potential and increased mitophagy. Correlative light and electron microscopy further reveals αSyn oligomers associated with mitochondrial membranes displaying structural abnormalities. Together, these findings identify lipid droplets as drivers of aberrant αSyn phase separation and suggest that lipid droplet-rich condensates contribute to mitochondrial dysfunction and impaired energy homeostasis. Given the enrichment of lipid droplets within neuromelanin-containing dopaminergic neurons of the substantia nigra, this mechanism may be particularly relevant to the selective neuronal vulnerability observed in Parkinson's disease.

EMBO press papers are accompanied online by A) a short (1-2 sentences) summary of the findings and their significance, B) 2-3 bullet points highlighting key results and C) a synopsis image that is exactly 550 pixels wide and 300 pixels high. The synopsis image should provide a sketch of the major findings, like a graphical abstract. Please note that text needs to be readable at the final size. Please send us this information along with the final manuscript.

We have included these new items as attachments and are also copying them below.

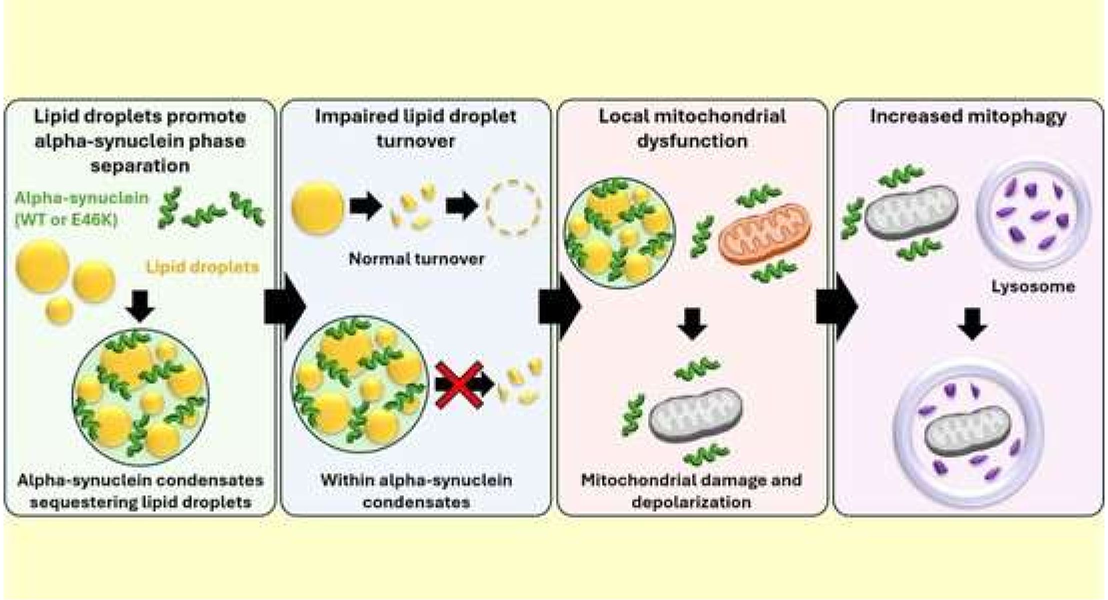

Summary
Lipid droplets promote the aberrant phase separation of alpha-synuclein into biomolecular condensates that disrupt lipid metabolism and mitochondrial homeostasis. These findings identify a mechanistic link between lipid droplets, alpha-synuclein dysregulation, and cellular dysfunction in Parkinson's disease.

Key findings

• Lipid droplets induce the formation of alpha-synuclein condensates by wild-type and E46K mutant alpha-synuclein, supporting a role for lipid metabolism in aberrant phase separation.

• Alpha-synuclein condensates sequester lipid droplets and impair their turnover, suggesting disruption of cellular energy homeostasis.

• Mitochondria adjacent to alpha-synuclein condensates are depolarized and undergo increased mitophagy, while correlative light and electron microscopy reveals alpha-synuclein oligomers associated with structurally abnormal mitochondrial membranes.

REVIEWER COMMENTS

Referee #1:

The reviewer greatly appreciates the extensive revisions and the substantial amount of new experimental data provided by the authors. The manuscript has been significantly improved, and the authors have thoroughly addressed the major mechanistic concerns raised in the previous round. To further enhance the readability and technical rigor of this study, the following final points are kindly suggested for consideration.

1. Enhancing Readability and Figure Referencing
With the addition of several new experimental panels, the manuscript has become substantially more data-rich. To further improve readability and ease of navigation, it would be helpful if the authors could clearly specify the corresponding figure and panel number for each result described in the text, rather than referring to multiple figures or panels collectively.

We have specified the panel number for each figure in the text where appropriate.

2. Optimizing Western Blot for Total aSyn (Fig. 3K)

The analysis in Figure 1, using cells whose YFP fluorescence levels were matched, is excellent and highly commendable. However, there remains a concern regarding Figure 3K: both the total and phosphorylated aSyn signals in WT and 1K cells appear quite faint and almost undetectable. To completely clear any ambiguity regarding the baseline expression levels, it would be beneficial if the authors could optimize this Western blot-perhaps by increasing the exposure time-so that the total aSyn signals in WT and 1K cells becomes visible. Providing a clear panel showcasing total aSyn with an appropriate loading control would further support the presence of the protein.

To improve visualization of αSyn expression levels, we have included higher exposure versions of the Western blots shown in Figure 3K as additional panels in Figure EV 1. These images clearly demonstrate the presence of total αSyn bands in WT and 1K cells and confirm the absence of a detectable αSyn-YFP band in uninduced 3K cells. The figure and legend are shown below.

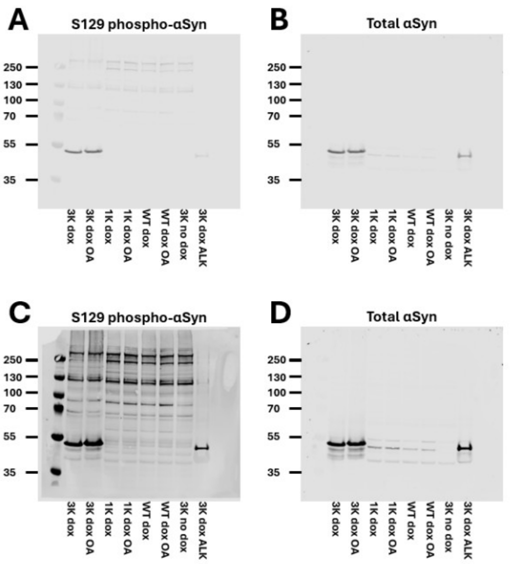

Figure EV 1: Oleic acid increases S129 phosphorylation of αSyn. A,B. Uncropped versions of the Western blots in figure 3K. C,D. Higher-exposure versions of the same Western blots, demonstrating the presence of total αSyn bands in WT and 1K cells and confirming the absence of a detectable αSyn-YFP band in 3K cells without doxycycline induction. WT and 1K αSyn do not show a detectable band corresponding to S129 phosphorylation under these experimental conditions, although phosphorylation levels may be below the detection limit of the assay. The 3K αSyn bands exhibit reduced electrophoretic mobility, consistent with the extensive S129 phosphorylation previously reported for this variant (Eubanks et al., 2025; Imberdis et al., 2019; Ramalingam et al., 2023a). Total αSyn protein levels are higher in 3K cells than in WT and 1K cells.

3. Refining Methodology and Logical Consistency in Figure EV2A
Figure EV2A provides an insightful spatial analysis, but it would benefit from refinement to ensure it is fully informative and clear for the readership. First, the current X-axis label "distance from inclusion" might be slightly misleading. Since the figure legend indicates that the analysis defines two distinct domains-the expanded inclusion rim and the remaining cytoplasm-by step-by-step expansion, the X-axis label would be more accurate if revised to reflect the actual parameter, such as Expansion distance from inclusion boundary (μm).

We modified the x axis label to Expansion distance from inclusion boundary, as suggested.

Additionally, please convert the "pixels" into standard physical units (μm) to provide better biological context.

We have added the pixel-to-distance conversion factor to the figure legend (0.071 µm/pixel), allowing all distances to be readily converted into standard physical units (µm). We retained the original axis labeling to preserve figure readability.

Second, since the cell is divided into these two regions at each step, the sum of the mitophagy events from both regions would be expected to remain relatively constant across all X values, as their total must always equal the whole-cell mitophagy events. However, this does not appear to be the case in the current graph. In particular, since cells often contain multiple inclusions, how did the analysis handle potential spatial overlaps when the boundaries of neighboring inclusions expanded and intersected? Clarification of these points, including the apparent non-constant sum of mitophagy events and the handling of overlapping regions, would help readers better understand the methodology.

The concern arose from an insufficiently descriptive y-axis label in the original figure. We have now corrected the label to "Normalized Density of Mitophagy Events / Cell". The plotted values represent the density of mitophagy events, in other words the number of mitophagy events normalized to the surface area of each region rather than the absolute number of mitophagy events. Specifically, the number of mitophagy events was divided by the surface area of the expanded inclusion region or the remaining cytoplasmic region to account for the substantially larger area of the cytoplasm and the correspondingly greater number of mitochondria present in that compartment. Because the plotted values are area-normalized densities, the sum of the two curves is not expected to remain constant across different pixel radii.

To further clarify this point, we have added a new panel (**Fig. EV 4 B**) showing the absolute number of mitophagy events in each region without area normalization. As expected, the total number of mitophagy events is substantially higher in the cytoplasm than near inclusions, and the number of cytoplasmic events decreases as the inclusion boundary is progressively expanded.

Regarding overlapping regions, the analysis pipeline generated binary masks corresponding to the expanded inclusion region and the remaining cytoplasmic region for each pixel radius. Mitophagy events were assigned based on their intersection with these masks and were counted only once. Consequently, overlapping expanded boundaries did not result in double counting of mitophagy events.

The new and modified panels in that figure (now labelled figure EV 4) are shown below.

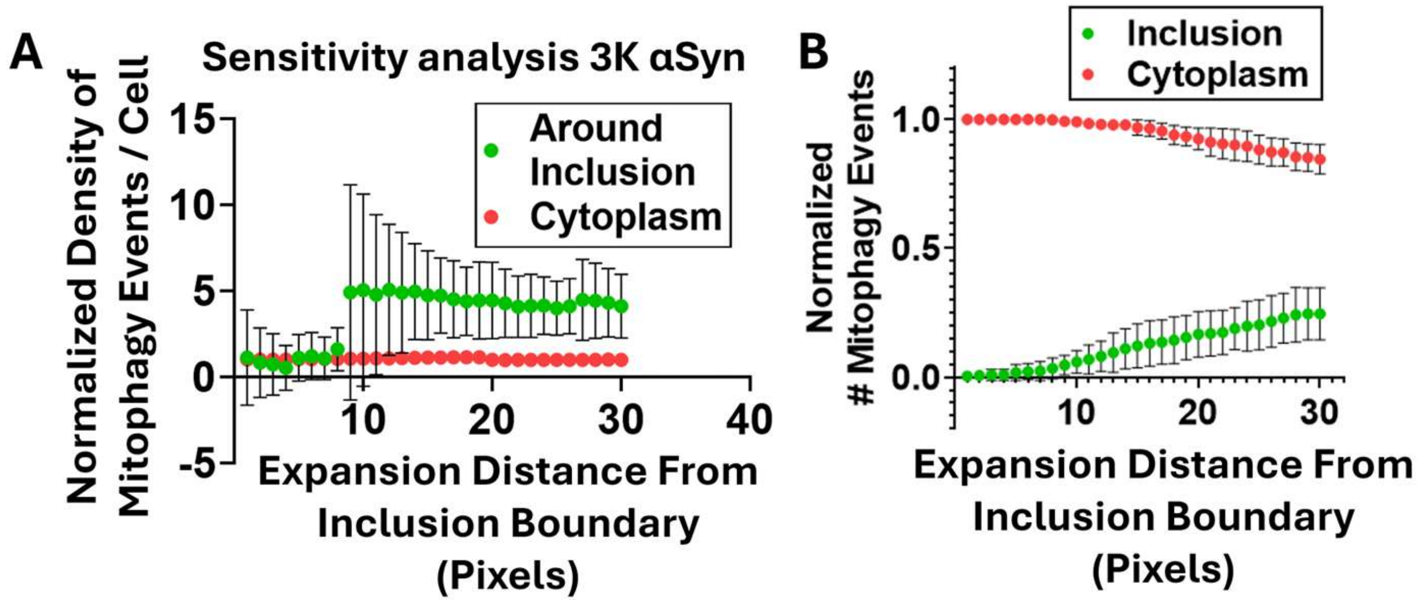

Furthermore, the authors conclude that a 25-pixel distance is appropriate, yet the graph shows a complete plateau from 9 to 30 pixels. To strengthen the rationale, it would be helpful if the authors could further explain why 25 pixels was selected, as the observed effect already appears to have reached saturation at 9 pixels.

The purpose of the sensitivity analysis was to determine whether the conclusions depended on the specific radius selected for the analysis. As shown in the revised plot, the observed effect reaches a plateau at approximately 9 pixels and remains stable across the range of 9-30 pixels. Thus, the conclusions are not sensitive to the precise radius chosen within this interval. We retained the original value of 25 pixels for consistency with the analyses presented in the initial submission, but any radius within the plateau region yields essentially identical results.

4. Clarifying the Conceptual Model (Figure 10)
To avoid any potential confusion for the readership, we suggest that the conceptual model in Figure 10 clearly distinguish between experimentally supported and speculative mechanisms. Since some specific events were not biochemically measured in this study, these unverified events should either be removed from the diagram or clearly illustrated as speculative, for example by using dashed arrows or labeling them as hypothetical.

In the revised Figure 10, relationships that were not directly measured or experimentally established in this study are now indicated by dashed arrows.

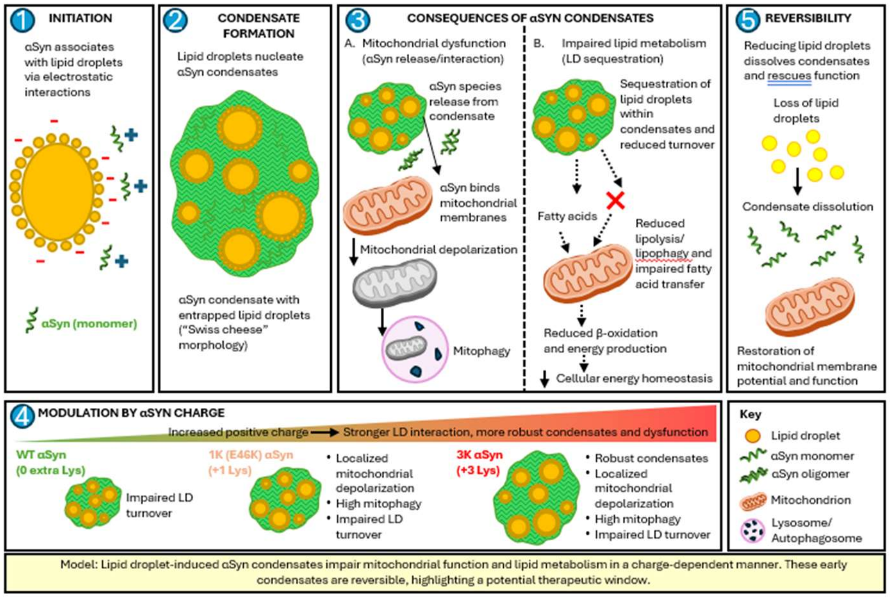

Referee #2:

My concerns have been addressed.

Referee #3:

The authors addressed all my points. Thus I recommend this manuscript for publication.

Referee #4:

The authors have addressed all my concerns. The manuscript is well written, presents exciting data, and is quite interesting. I recommend this paper be published.

Dr. Eleanna Kara
Rutgers University
Neurology
United States

Dear Eleanna,

I am very pleased to accept your manuscript for publication in the next available issue of EMBO reports. Thank you for your contribution to our journal.

Your manuscript will be processed for publication by EMBO Press. It will be copy edited and you will receive page proofs prior to publication. Please note that you will be contacted by Springer Nature Author Services to complete licensing and payment information.

You may qualify for financial assistance for your publication charges - either via a Springer Nature fully open access agreement or an EMBO initiative. Check your eligibility: https://link.springer.com/journal/44319/how-to-publish-with-us

Should you be planning a Press Release on your article, please notify the production team via this form in order to coordinate publication and release dates: https://forms.cloud.microsoft/e/eH4A8JVP6g

If you have any questions, please do not hesitate to contact the Editorial Office. Thank you for your contribution to EMBO Reports.

Best regards,
Esther

Esther Schnapp, PhD
Senior Editor
EMBO reports

>>> Please note that it is EMBO Reports policy for the transcript of the editorial process (containing referee reports and your response letter) to be published as an online supplement to each paper. If you do NOT want this, you will need to inform the Editorial Office via email immediately. More information is available here: https://link.springer.com/partners/embo-press/editorial-policies#Peer%20review

EMBO Press Author Checklist

Corresponding Author Name: Eleanna Kara	
Journal Submitted to: EMBO Reports	
Manuscript Number: EMBOR-2025-63242V1	

USEFUL LINKS FOR COMPLETING THIS FORM
The EMBO Journal - Author Guidelines
EMBO Reports - Author Guidelines
Molecular Systems Biology - Author Guidelines
EMBO Molecular Medicine - Author Guidelines

Reporting Checklist for Life Science Articles (updated January
This checklist is adapted from Materials Design Analysis Reporting (MDAR) Checklist for Authors. MDAR establishes a minimum set of requirements in transparent reporting in the life sciences (see Statement of Task: 10.31222/osf.io/9sm4x). Please follow the journal's guidelines in preparing your
Please note that a copy of this checklist will be published alongside your article.

Abridged guidelines for figures
1. Data
The data shown in figures should satisfy the following conditions:
- ➞ the data were obtained and processed according to the field's best practice and are presented to reflect the results of the experiments in an accurate and unbiased manner.
- ➞ ideally, figure panels should include only measurements that are directly comparable to each other and obtained with the same assay.
- ➞ plots include clearly labeled error bars for independent experiments and sample sizes. Unless justified, error bars should not be shown for technical
- ➞ if n<5, the individual data points from each experiment should be plotted. Any statistical test employed should be justified.
- ➞ Source Data should be included to report the data underlying figures according to the guidelines set out in the authorship guidelines on Data

2. Captions
Each figure caption should contain the following information, for each panel where they are relevant:
- ➞ a specification of the experimental system investigated (eg cell line, species name).
- ➞ the assay(s) and method(s) used to carry out the reported observations and measurements.
- ➞ an explicit mention of the biological and chemical entity(ies) that are being measured.
- ➞ an explicit mention of the biological and chemical entity(ies) that are altered/varied/perturbed in a controlled manner.
- ➞ the exact sample size (n) for each experimental group/condition, given as a number, not a range;
- ➞ a description of the sample collection allowing the reader to understand whether the samples represent technical or biological replicates (including how many animals, litters, cultures, etc.).
- ➞ a statement of how many times the experiment shown was independently replicated in the laboratory.
- ➞ definitions of statistical methods and measures:
  - common tests, such as t-test (please specify whether paired vs. unpaired), simple $\chi 2$ tests, Wilcoxon and Mann-Whitney tests, can be unambiguously identified by name only, but more complex techniques should be described in the methods section;
  - are tests one-sided or two-sided?
  - are there adjustments for multiple comparisons?
  - exact statistical test results, e.g., P values = x but not P values < x;
  - definition of 'center values' as median or average;
  - definition of error bars as s.d. or s.e.m.

Please complete ALL of the questions below.
Select "Not Applicable" only when the requested information is not relevant for your study.

Materials

Newly Created Materials	Information included in the manuscript?	In which section is the information available? (Reagents and Tools Table, Materials and Methods, Figures, Data Availability Section)
New materials and reagents need to be available; do any restrictions apply?	Yes	Reagents and Tools Table, Materials and Methods. 3K, 1K and WT synuclein cell lines are available through Prof. Ulf Dettmer.

Antibodies	Information included in the manuscript?	In which section is the information available? (Reagents and Tools Table, Materials and Methods, Figures, Data Availability Section)
For **antibodies** provide the following information:<br>- Commercial antibodies: RRID (if possible) or supplier name, catalogue number and or/clone number<br>- Non-commercial: RRID or citation	Yes	Reagents and Tools Table

DNA and RNA sequences	Information included in the manuscript?	In which section is the information available? (Reagents and Tools Table, Materials and Methods, Figures, Data Availability Section)
**Short novel DNA or RNA including primers, probes**: provide the sequences.	Not Applicable	

Cell materials	Information included in the manuscript?	In which section is the information available? (Reagents and Tools Table, Materials and Methods, Figures, Data Availability Section)
**Cell lines:** Provide species information, strain. Provide accession number in repository **OR** supplier name, catalog number, clone number, and/**OR** RRID.	Yes	Reagents and Tools Table, Materials and Methods
**Primary cultures:** Provide species, strain, sex of origin, genetic modification status.	Not Applicable	
Report if the cell lines were recently **authenticated** (e.g., by STR profiling) and tested for mycoplasma contamination.	Yes	Materials and Methods

Experimental animals	Information included in the manuscript?	In which section is the information available? (Reagents and Tools Table, Materials and Methods, Figures, Data Availability Section)
**Laboratory animals or Model organisms:** Provide species, strain, sex, age, genetic modification status. Provide accession number in repository **OR** supplier name, catalog number, clone number, **OR** RRID.	Not Applicable	
**Animal observed in or captured from the field:** Provide species, sex, and age where possible.	Not Applicable	
Please detail **housing and husbandry conditions**.	Not Applicable	

Plants and microbes	Information included in the manuscript?	In which section is the information available? (Reagents and Tools Table, Materials and Methods, Figures, Data Availability Section)
**Plants:** provide species and strain, ecotype and cultivar where relevant, unique accession number if available, and source (including location for collected wild specimens).	Not Applicable	
**Microbes:** provide species and strain, unique accession number if available, and source.	Yes	Reagents and Tools Table. One Shot™ MAX Efficiency™ DH5α-T1R Competent Cells

Human research participants	Information included in the manuscript?	In which section is the information available? (Reagents and Tools Table, Materials and Methods, Figures, Data Availability Section)
If collected and within the bounds of privacy constraints report on age, sex and gender or ethnicity for all study participants.	Not Applicable	

Core facilities	Information included in the manuscript?	In which section is the information available? (Reagents and Tools Table, Materials and Methods, Figures, Data Availability Section)
If your work benefited from core facilities, was their service mentioned in the acknowledgments section?	Yes	Acknowledgments

Design

Study protocol	Information included in the manuscript?	In which section is the information available? (Reagents and Tools Table, Materials and Methods, Figures, Data Availability Section)
If study protocol has been **pre-registered, provide DOI in the manuscript**. For clinical trials, provide the trial registration number **OR** cite DOI.	Not Applicable	
Report the **clinical trial registration number** (at ClinicalTrials.gov or equivalent), where applicable.	Not Applicable	

Laboratory protocol	Information included in the manuscript?	In which section is the information available? (Reagents and Tools Table, Materials and Methods, Figures, Data Availability Section)
Provide DOI OR other citation details if **external detailed step-by-step protocols** are available.	Not Applicable	

Experimental study design and statistics	Information included in the manuscript?	In which section is the information available? (Reagents and Tools Table, Materials and Methods, Figures, Data Availability Section)
Include a statement about **sample size** estimate even if no statistical methods were used.	Yes	Materials and Methods.
Were any steps taken to minimize the effects of subjective bias when allocating animals/samples to treatment (e.g. **randomization procedure**)? If yes, have they been described?	Not Applicable	
Include a statement about **blinding** even if no blinding was done.	Yes	Materials and Methods.
Describe **inclusion/exclusion criteria** if samples or animals were excluded from the analysis. Were the criteria pre-established?<br><br>If sample or data points were omitted from analysis, report if this was due to attrition or intentional exclusion and provide justification.	Not Applicable	
For every figure, are **statistical tests** justified as appropriate? Do the data meet the assumptions of the tests (e.g., normal distribution)? Describe any methods used to assess it. Is there an estimate of variation within each group of data? Is the variance similar between the groups that are being statistically compared?	Yes	Materials and Methods.

Sample definition and in-laboratory replication	Information included in the manuscript?	In which section is the information available? (Reagents and Tools Table, Materials and Methods, Figures, Data Availability Section)
In the figure legends: state number of times the experiment was **replicated** in laboratory.	Yes	Figures
In the figure legends: define whether data describe **technical or biological replicates**.	Yes	Figures

Ethics

Ethics	Information included in the manuscript?	In which section is the information available? (Reagents and Tools Table, Materials and Methods, Figures, Data Availability Section)
Studies involving **human participants**: State details of **authority granting ethics approval** (IRB or equivalent committee(s), provide reference number for approval.	Not Applicable	
Studies involving **human participants**: Include a statement confirming that **informed consent** was obtained from all subjects and that the experiments conformed to the principles set out in the WMA Declaration of Helsinki and the Department of Health and Human Services Belmont Report.	Not Applicable	
Studies involving **human participants:** For publication of **patient photos**, include a statement confirming that consent to publish was obtained.	Not Applicable	
Studies involving experimental **animals**: State details of **authority granting ethics approval** (IRB or equivalent committee(s), provide reference number for approval. Include a statement of compliance with ethical regulations.	Not Applicable	
Studies involving **specimen and field samples:** State if relevant **permits** obtained, provide details of authority approving study; if none were required, explain why.	Not Applicable	

Dual Use Research of Concern (DURC)	Information included in the manuscript?	In which section is the information available? (Reagents and Tools Table, Materials and Methods, Figures, Data Availability Section)
Could your study fall under dual use research restrictions? Please check biosecurity documents and list of **select agents and toxins** (CDC): https://www.selectagents.gov/sat/list.htm	Not Applicable	
If you used a select agent, is the security level of the lab appropriate and reported in the manuscript?	Not Applicable	
If a study is subject to dual use research of concern regulations, is the name of the **authority granting approval and reference number** for the regulatory approval provided in the manuscript?	Not Applicable	

Reporting

The MDAR framework recommends adoption of discipline-specific guidelines, established and endorsed through community initiatives. Journals have their own policy about requiring specific guidelines and recommendations to complement MDAR.

Adherence to community standards	Information included in the manuscript?	In which section is the information available? (Reagents and Tools Table, Materials and Methods, Figures, Data Availability Section)
State if relevant guidelines or checklists (e.g., **ICMJE, MIBBI, ARRIVE, PRISMA**) have been followed or provided.	Not Applicable	
For **tumor marker prognostic studies,** we recommend that you follow the **REMARK** reporting guidelines (see link list at top right). See author guidelines, under 'Reporting Guidelines'. Please confirm you have followed these guidelines.	Not Applicable	
For **phase II and III randomized controlled trials**, please refer to the **CONSORT** flow diagram (see link list at top right) and submit the CONSORT checklist (see link list at top right) with your submission. See author guidelines, under 'Reporting Guidelines'. Please confirm you have submitted this list.	Not Applicable	

Data Availability

Data availability	Information included in the manuscript?	In which section is the information available? (Reagents and Tools Table, Materials and Methods, Figures, Data Availability Section)
Have **primary datasets** been deposited according to the journal's guidelines (see 'Data Deposition' section) and the respective accession numbers provided in the Data Availability Section?	Yes	Data availability section.
Were **human clinical and genomic datasets** deposited in a public access-controlled repository in accordance to ethical obligations to the patients and to the applicable consent agreement?	Not Applicable	
Are **computational models** that are central and integral to a study available without restrictions in a machine-readable form? Were the relevant accession numbers or links provided?	Yes	Data availability section.
If publicly available data were reused, provide the respective **data citations in the reference list**.	Not Applicable	
