# Supplementary material for: Lipid droplets promote aberrant liquid-liquid phase separation of alpha-synuclein impairing energy homeostasis
Source: EMBO Rep. 2026 Jul 2;27(15):4299–331. doi: 10.1038/s44319-026-00856-8 (PMC13458734; doi:10.1038/s44319-026-00856-8)
Supplement: Supplementary file 2 [file 44319_2026_856_MOESM4_ESM.pdf]

Expanded View Figures

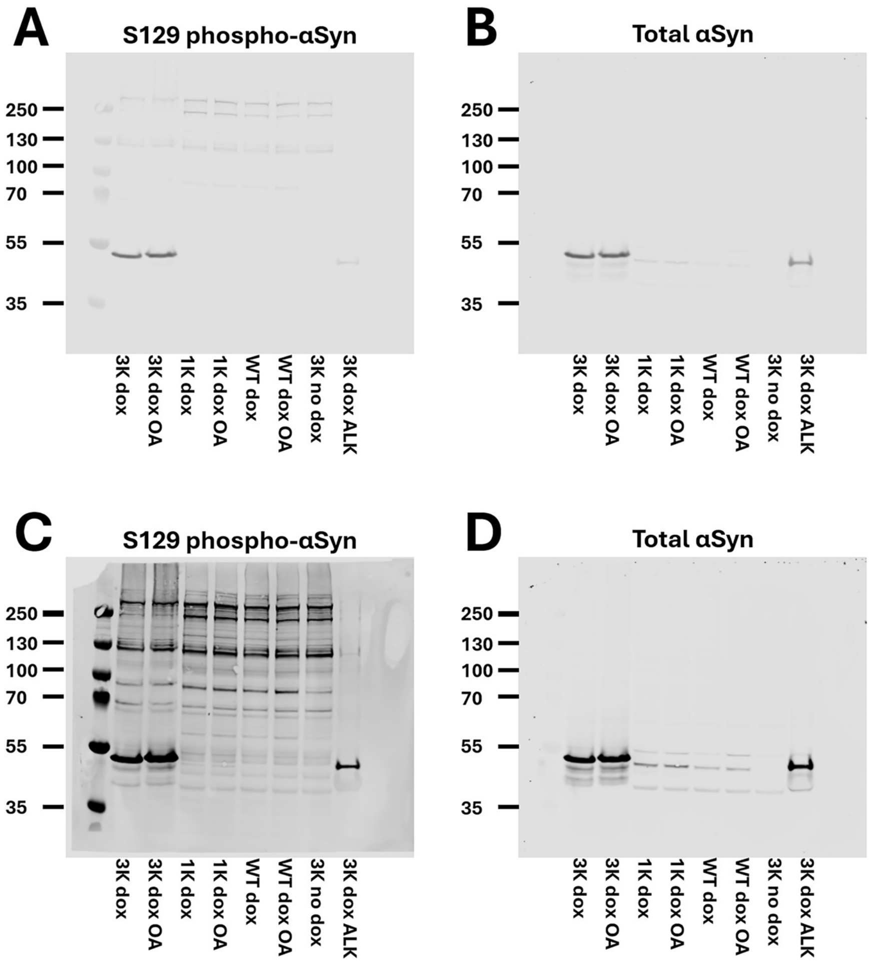

Figure EV1. Oleic acid increases S129 phosphorylation of αSyn.

(**A**, **B**) Uncropped versions of the Western blots in Fig. 3K. (**C**, **D**) Higher-exposure versions of the same western blots, demonstrating the presence of total αSyn bands in WT and 1K cells and confirming the absence of a detectable αSyn-YFP band in 3K cells without doxycycline (dox) induction. WT and 1K αSyn do not show a detectable band corresponding to S129 phosphorylation under these experimental conditions, although phosphorylation levels may be below the detection limit of the assay. The 3K αSyn bands exhibit reduced electrophoretic mobility, consistent with the extensive S129 phosphorylation previously reported for this variant (Eubanks et al, 2025; Imberdis et al, 2019; Ramalingam et al, 2023a). Total αSyn protein levels are higher in 3K cells than in WT and 1K cells. OA, oleic acid. (Supplementary Fig. 1 in BioImage Archive repository).

 © The Author(s)

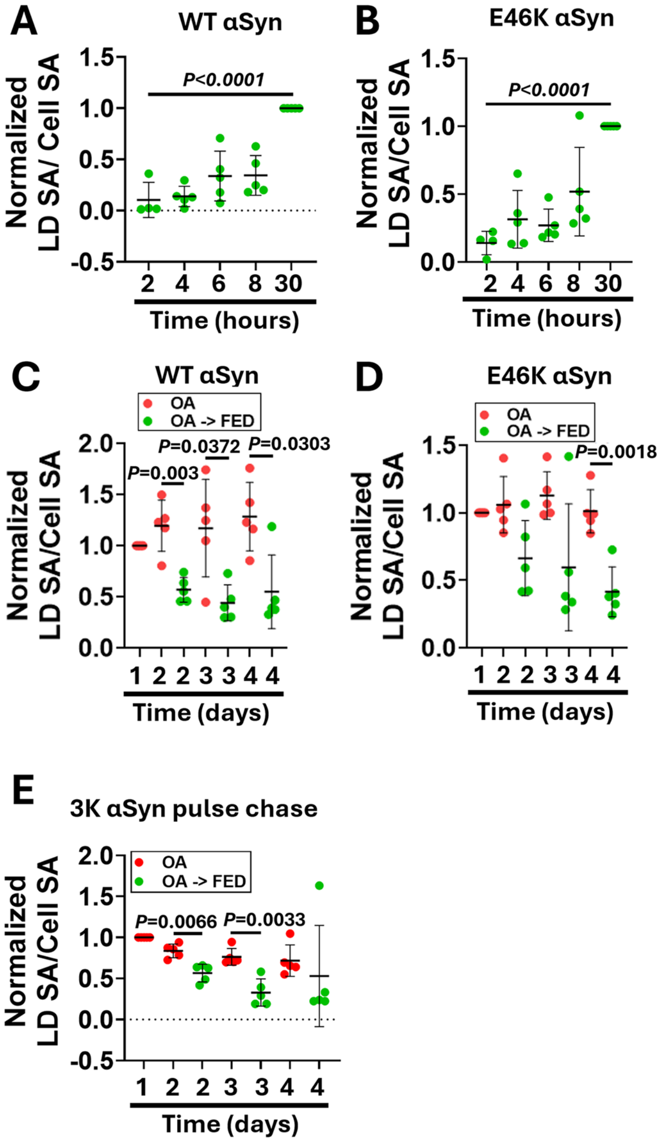

Figure EV2. Lipid droplets reduce over time in an oleic acid pulse-chase experiment.

(**A**, **B**) WT and 1K cells were induced with doxycycline and imaged at 2, 4, 6, 8 and 30 h post-induction to quantify the surface area (SA) of lipid droplets in relation to the surface area of the cell. One-way ANOVA with test for linear trend. (**C–E**) WT, 1K and 3K cells were induced with doxycycline for 48 h, treated with oleic acid (OA) for 24 h and imaged (day 1); subsequently, their media was replaced with media without OA and imaged every 24 h over 3 days. The surface area of lipid droplets was quantified in relation to the surface area of the cytoplasm. Unpaired *t* test with Bonferroni correction. All experiments were repeated independently five times, and the normalized values are shown in each plot. Data are represented as mean +/− SD. (Supplementary Fig. 2 in BioImage Archive repository).

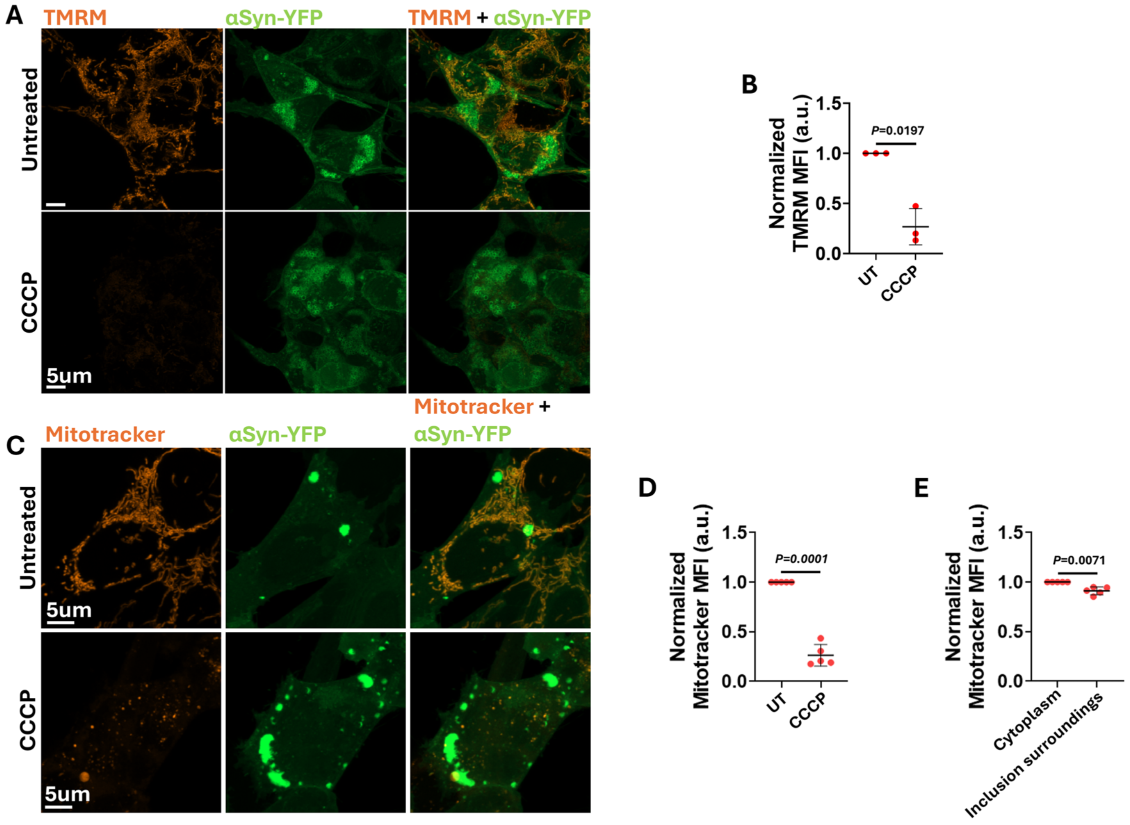

Figure EV3. Validation of mitochondrial experiments.

(**A**, **B**) Treatment of 3K cells with carbonyl cyanide m-chlorophenyl hydrazone (CCCP) results in mitochondrial depolarization, as quantified by the reduction of the mean fluorescence intensity (MFI) of tetramethylrhodamine methyl ester (TMRM). One-sample *t* test. (**C**, **D**) MFI of MitoTracker CMXRos was used to measure the mitochondrial membrane potential. Treatment with CCCP resulted in a significant reduction in MitoTracker CMXRos MFI. One-sample *t* test. (**E**) MitoTracker CMXRos showed reduced MFI in a 7-pixel radius around 3K αSyn condensates. One-sample *t* test. All experiments were repeated independently five times, and the normalized values are shown in each plot. Data are represented as mean +/− SD. a.u. arbitrary units. (Supplementary Fig. 3 in BioImage Archive repository).

 © The Author(s)

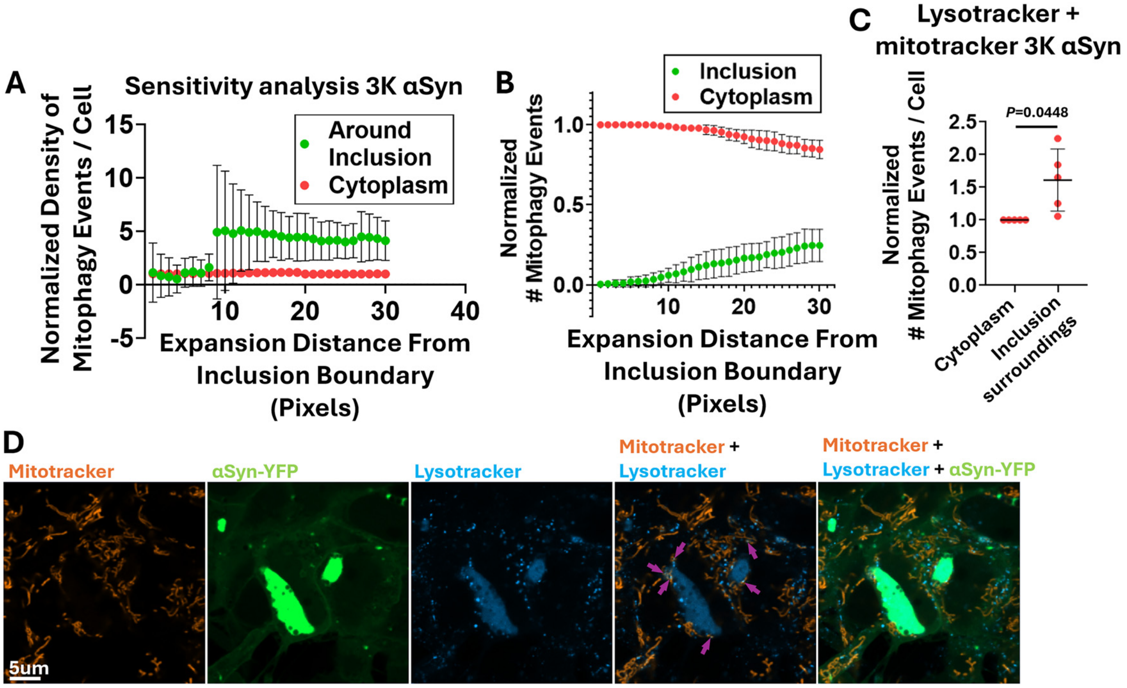

Figure EV4. Validation of mitophagy experiments.

(**A**) The mitophagy images from Fig. 6C (3K cells) were re-analyzed in sensitivity format. αSyn condensates were dilated by 1-pixel radius at a time. Mitophagy events were quantified within the dilated rim surrounding the condensates as well as in the remaining cytoplasm. The number of mitophagy events was normalized to the surface area (SA) of the dilated rim or the cytoplasm, respectively. No statistical analysis was undertaken. 0.071 µm/pixel. (**B**) The data from (**A**) are shown without normalization to surface area. (**C**, **D**) Live 3K cells were stained with Lysotracker Blue and Mitotracker red, followed by colocalization analysis to identify mitophagy events using the same workflow as in Fig. 6. Bleedthrough was present from the green channel (αSyn-YFP) to the blue channel (Lysotracker), which was subtracted through Python code during analysis. Mitophagy events are shown in purple arrows. One-sample *t* test. All experiments were repeated independently six times for the sensitivity analysis on the 3K cells and five times for lysotracker+mitotracker experiment, and the normalized values are shown in each plot. Data are represented as mean +/− SD. (Supplementary Fig. 4 in BioImage Archive repository).

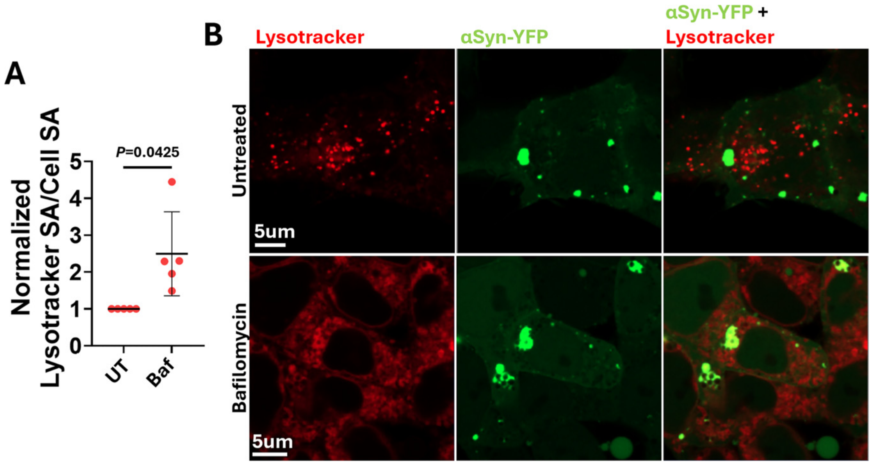

Figure EV5. Bafilomycin A1 treatment increases the lysosomal area.

(**A**, **B**) 3K cells were treated with 100 nM of Bafilomycin A1 for 24 h and stained with Lysotracker Deep Red to visualize the lysosomes. The surface area (SA) of the lysosomes was divided with the SA of the cells on a cell-by-cell basis. Comparison was made between Bafilomycin-treated and untreated cells. One-sample *t* test. Data are represented as mean +/− SD. Baf=Bafilomycin A1. (Supplementary Fig. 5 in BioImage Archive repository).

 © The Author(s)

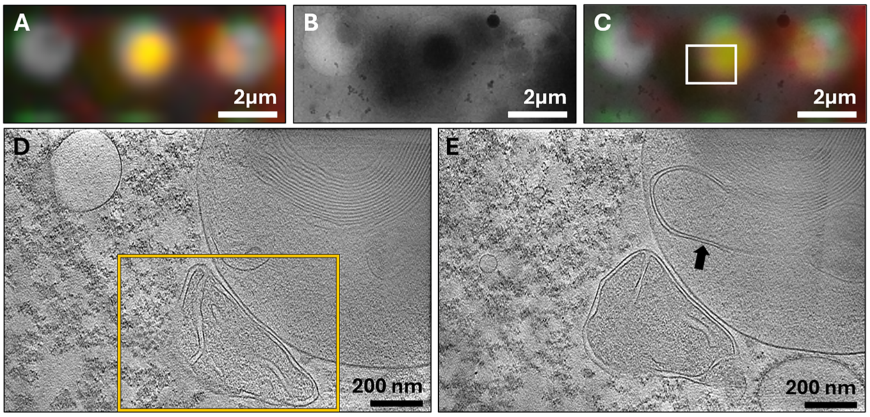

Figure EV6. Correlative light and electron microscopy (CLEM) and cryo-electron tomography (cryo-ET) reveal intensive direct interactions between αSyn structures and the mitochondrial outer membrane in cell lysates from 3K M17D cells.

(**A–C**) CLEM analysis showing cryo-fluorescence (**A**), low-magnification cryo-EM (**B**), and correlated overlay (**C**) of αSyn (green) and mitochondria (red) in cell lysates from 3K M17D cells. (**D**) Slice view of a tomogram from the boxed area in (**C**), depicting αSyn association on the outer membrane of a mitochondrion and inducing pronounced morphological alterations (orange box). (**E**) A different slice from the same tomogram, highlighting a multivesicular body enclosing a mitochondrion (black arrow).

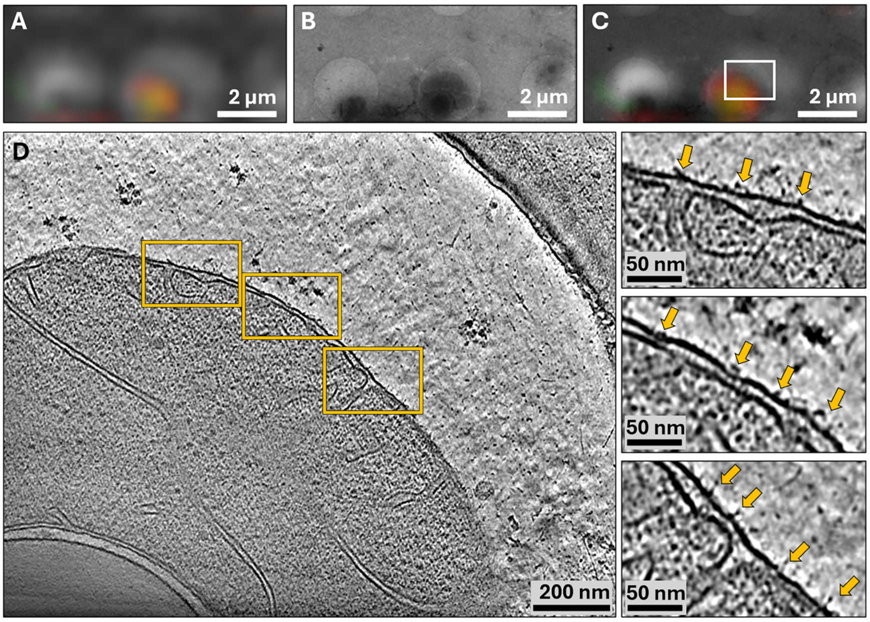

Figure EV7. CLEM and cryo-ET shows association of small putative αSyn species on the outer mitochondrial membrane.

(**A–C**) CLEM analysis showing cryo-fluorescence (**A**), low-magnification cryo-EM (**B**), and correlated overlay (**C**) of αSyn (green) and mitochondria (red) in cell lysates from 3K M17D cells. (**D**) Slice view of a tomogram from the boxed area in (**C**), showing smaller αSyn species, suggestive of oligomers, associated on the outer membrane of a mitochondrion. Boxed insets highlight zoomed-in regions with αSyn species and areas of mitochondrial membrane roughness (orange arrows).

 © The Author(s)

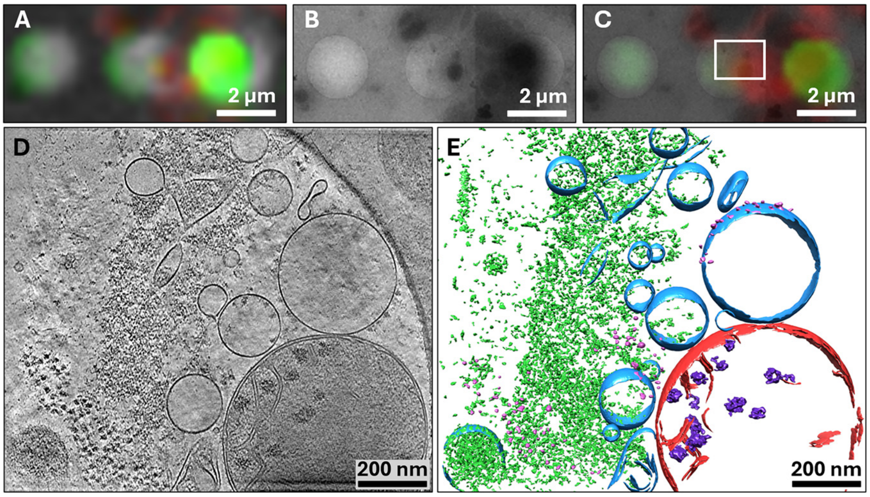

Figure EV8. CLEM and cryo-ET reveal αSyn species and mitochondrial structures in cell lysates from 3K M17D cells.

(**A–C**) CLEM analysis showing cryo-fluorescence (**A**), low-magnification cryo-EM (**B**), and correlated overlay (**C**) of αSyn (green) and mitochondria (red) in cell lysates from 3K M17D cells. The image in (**B**) is a stitched tiles of low-magnification EM images. The vertical line represents the boundary between the two images. (**D**, **E**) Tomographic slice (**D**) and 3D annotated view (**E**) of a tomogram from the boxed region in (**C**), showing an αSyn network in proximity to a mitochondrion. The αSyn network does not directly interact with the mitochondrial membrane. The mitochondrion exhibits a representative smooth, spherical morphology. Cellular vesicular structures embedded within the αSyn network display signs of membrane deformation. The mitochondrial membrane is shown in red, αSyn in green, intramitochondrial granules in purple, other vesicular structures in blue, and ribosomes in pink.

© The Author(s)
